# Supplementary material for: A real-world multi-center RNA-seq benchmarking study using the Quartet and MAQC reference materials
Source: Nat Commun. 2024 Jul 22;15:6167. doi: 10.1038/s41467-024-50420-y (PMC11263697; doi:10.1038/s41467-024-50420-y)
Supplement: Supplementary file 1 — Supplementary Information File [file 41467_2024_50420_MOESM1_ESM.pdf]

Supplementary information for "A Real-World Multi-Center RNA-seq Benchmarking Study Using the Quartet and MAQC Reference Materials"

Table of Contents

1. Supplementary Figures **1**

Supplementary Figure 1. Comparison of SNR values for different combinations of reference material. 1

Supplementary Figure 2. The agreement of gene expression among laboratories for different gene types. 2

Supplementary Figure 3. The gene lengths and expression levels for five gene types...... 3

Supplementary Figure 4. The influence of gene lengths and gene expression on inter-laboratory reproducibility. 4

Supplementary Figure 5. PCA by all pooling RNA-seq data for different sample combinations........ 5

Supplementary Figure 6. Relative expression measurements were more accurate than absolute expression measurements. 6

Supplementary Figure 7. Comparison between laboratories with good and bad recovery of mixture ratios of sample T1 and T2. 7

Supplementary Figure 8. Presentation of genes in RNA-seq data with poor recovery of mixed ratios. 8

Supplementary Figure 9. The number of reads of RNA-seq data. 9

Supplementary Figure 10. The influence of different lanes on the sequencing depth..... 10

Supplementary Figure 11. Quality values of sequencing reads of RNA-seq data...........11

Supplementary Figure 12. Base quality values of representative sequencing read for the Quartet samples. 12

Supplementary Figure 13. Base quality values of representative sequencing read for the MAQC samples. 13

Supplementary Figure 14. GC distribution of RNA-seq data for Quartet samples. 14

Supplementary Figure 15. GC distribution of RNA-seq data for MAQC samples. 15

Supplementary Figure 16. Impact of GC content on inter-laboratory agreement of gene expression. 16

Supplementary Figure 17. The duplication rate of RNA-seq data. 17

Supplementary Figure 18. The correlation between duplication rates and sequencing depth and the mRNA enrichment methods. 18

Supplementary Figure 19. The duplication rate of reads mapped to coding genes. 19

Supplementary Figure 20. Distribution of abnormal duplication rates across laboratories and samples. 20

Supplementary Figure 21. The mapping statistic for all RNA-seq data. 21

Supplementary Figure 22. The influence of mRNA enrichment methods on multi-mapping rate. 21

Supplementary Figure 23. Percentage of mapped reads in exonic, intronic, and intergenic regions. 22
Supplementary Figure 24. Percentage of the exonic reads. 23
Supplementary Figure 25. SNR after applying the fixed data analysis pipeline. 24
Supplementary Figure 26. SNP-based sample-identity check across laboratories. 25
Supplementary Figure 27. Cross-contamination assessment based on ERCC controls. . 26
Supplementary Figure 28. Data quality (SNR values) after applying fixed analysis pipelines. 27
Supplementary Figure 29. The accuracy of absolute and relative expression after applying the fixed analysis pipeline (Ensembl-STAR-StringTie). 28
Supplementary Figure 30. The accuracy of absolute and relative expression after applying the fixed analysis pipeline (RefSeq-Salmon). 29
Supplementary Figure 31. PVCA of variations in absolute and relative based on different pipeline (RefSeq-Salmon). 30
Supplementary Figure 32. Calculation of relative expression could eliminate the variations from experimental processes. 31
Supplementary Figure 33. The benchmark analysis workflow. 32
Supplementary Figure 34. Calculating relative expression could correct the influences of different bioinformatics tools. 33
Supplementary Figure 35. Low accuracy for low-quality RNA-seq data. 34
Supplementary Figure 36. Comparisons of SNR between two mRNA enrichment methods for different gene types. 35
Supplementary Figure 37. The influence of read depths on RNA-seq performance. 36
Supplementary Figure 38. The impact of read length on the RNA-seq performance. 37
Supplementary Figure 39. The influence of experimental factors under different performance metrics 38
Supplementary Figure 40. The number of junctions detected with different sequencing depth. 39
Supplementary Figure 41. The number of junctions detected with different read lengths. 40
Supplementary Figure 42. Performance of different alignment schemes. 41
Supplementary Figure 43. Comparison of junctions detected by four alignment schemes. 42
Supplementary Figure 44. The proportion of reliable and unreliable junctions detected by the four alignment schemes. 43
Supplementary Figure 45. Scatterplots of PCA on RNA-seq data from the 28 quantification pipelines 44

Supplementary Figure 46. The impact of gene annotation on accuracy of relative expression. 45
Supplementary Figure 47. The impact of alignment tools on the accuracy of relative expression. 46
Supplementary Figure 48. The performance ranking of 28 quantification pipelines at relative expression levels. 47
Supplementary Figure 49. Comparison of the SNR of RNA-seq data from different normalization methods. 48
Supplementary Figure 50. Distribution of gene expression using different normalization methods. 49
Supplementary Figure 51. Quantitative assessment of low-expression gene filtering methods. 50
Supplementary Figure 52. Quantitative assessment of low-expression gene filtering for different differential analysis tools. 51
Supplementary Figure 53. Comparison of the maximal number of DEGs using different filtering methods. 52
Supplementary Figure 54. Comparison of the maximal sensitivity using different filtering methods. 53
Supplementary Figure 55. Comparison of the optimal threshold values determined by maximum number of DEGs and highest TPR. 54
Supplementary Figure 56. Comparison of the TPR corresponding to thresholds determined by maximum total number of DEGs and highest TPR. 55
Supplementary Figure 57. The assessment of five differential analysis tools using AUC values. 55
Supplementary Figure 58. Validation of ratio-based Quartet reference dataset. 56
2. Supplementary Tables 57
3. Supplementary Notes 60
3.1 The comprehensive performance assessment framework for RNA-seq data 60
3.2 RNA-seq assessment based on ERCC spike-in controls. 62
3.3 The number of detected genes in Quartet and MAQC samples. 64
Reference 67

1. Supplementary Figures

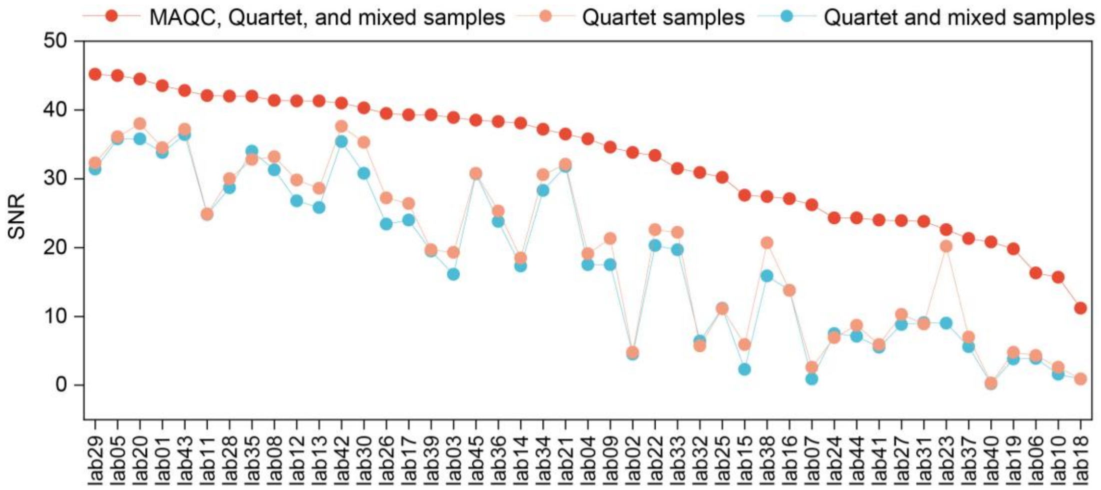

Supplementary Figure 1. Comparison of SNR values for different combinations of reference material. The red squares indicate the SNR values from all 24 MAQC and Quartet samples. The cyan triangles indicate the SNR values from M8, F7, D5, D6, T1, and T2, while blue circles indicate the SNR values from M8, F7, D5, and D6. SNR, signal-to-noise ratio.

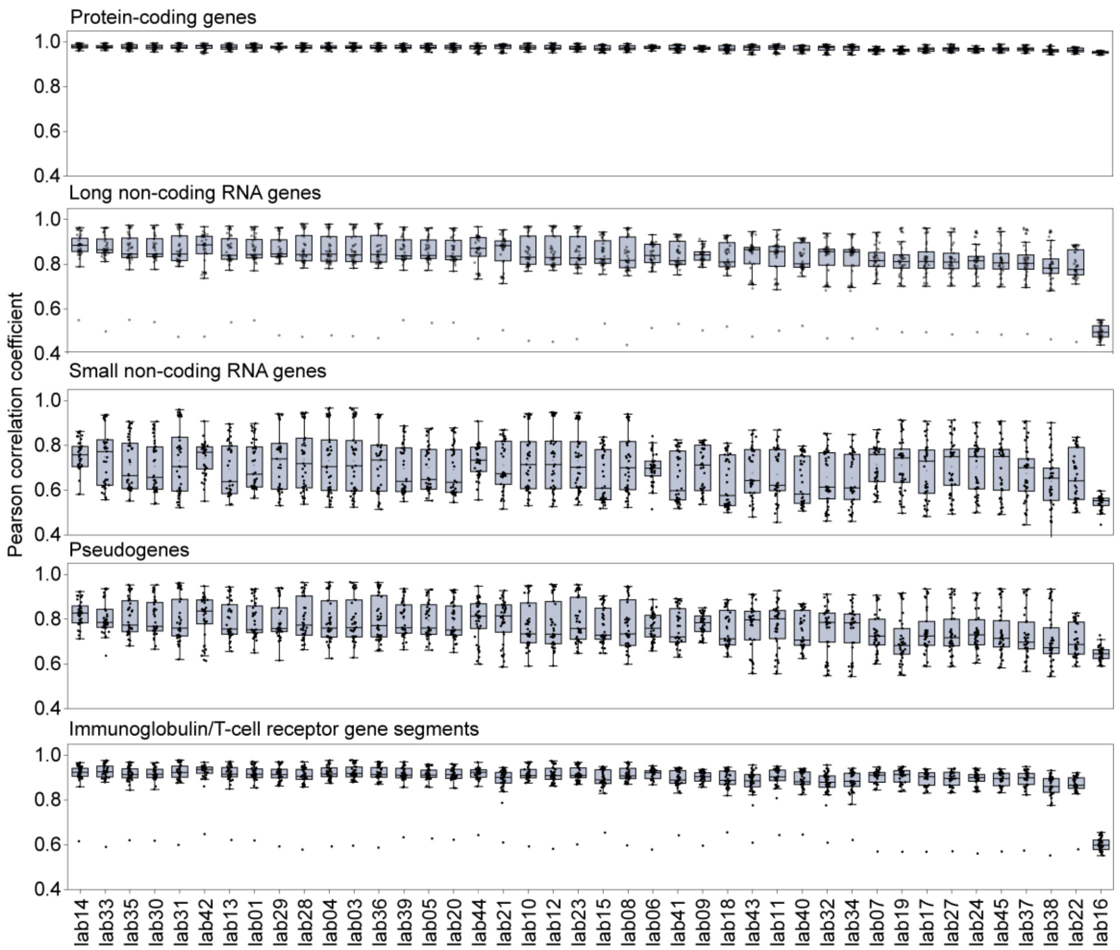

Supplementary Figure 2. The agreement of gene expression among laboratories for different gene types. After applying fixed analysis pipeline to RNA-seq data from 42 laboratories, the agreement among laboratories was notably high for protein-coding genes but comparatively lower for small non-coding genes and pseudogenes.

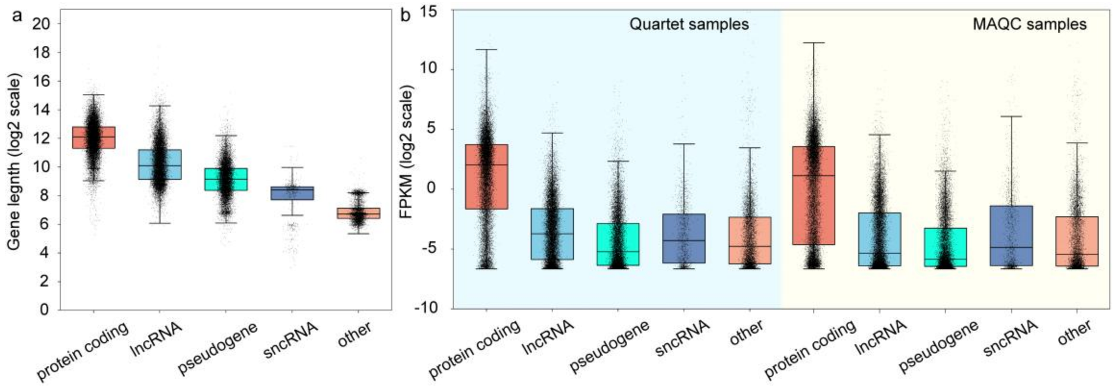

Supplementary Figure 3. The gene lengths and expression levels for five gene types. Five gene types include protein-coding genes (n = 20,048), long non-coding RNA (lncRNA) (n = 18,859), pseudogenes (n = 14,497), small non-coding RNA (sncRNA) (n = 7,578), and others (such as immunoglobulin/T-cell receptor gene segments) (n = 1,701). **(a)** Pseudogenes, sncRNA, and immunoglobulin/T-cell receptor gene segments are generally shorter in length, corresponding to lower inter-laboratory reproducibility **(Supplementary Figure 2)**. **(b)** In both the Quartet and MAQC samples, the average gene expression from all RNA-seq data from 42 laboratories is calculated. The lncRNA, sncRNA, immunoglobulin/T-cell receptor gene segments, and pseudogene exhibit lower expression levels compared to protein-coding genes, which could also explain the lower inter-laboratory reproducibility for these gene types **(Supplementary Figure 2)**.

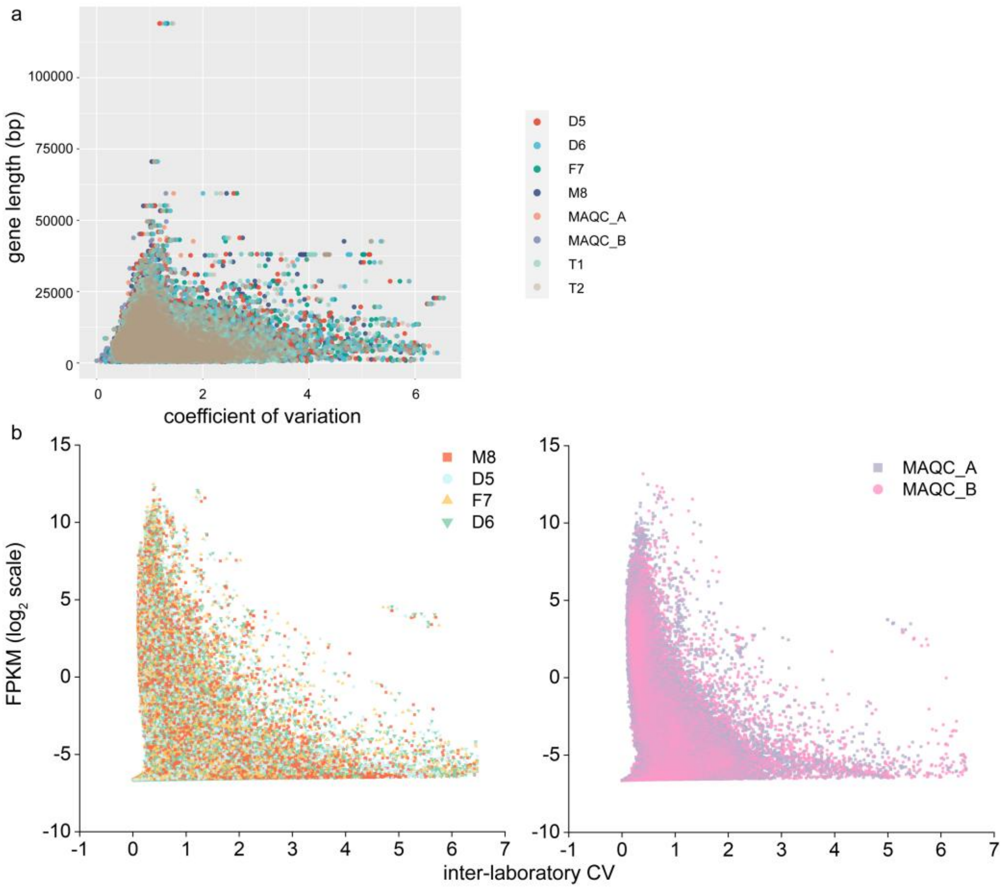

Supplementary Figure 4. The influence of gene lengths and gene expression on inter-laboratory reproducibility. (a) Genes with low gene length tended to have a high coefficient of variances (CV) across laboratories. (b) Low-expression genes showed a lower coefficient of variances (CV) across laboratories.

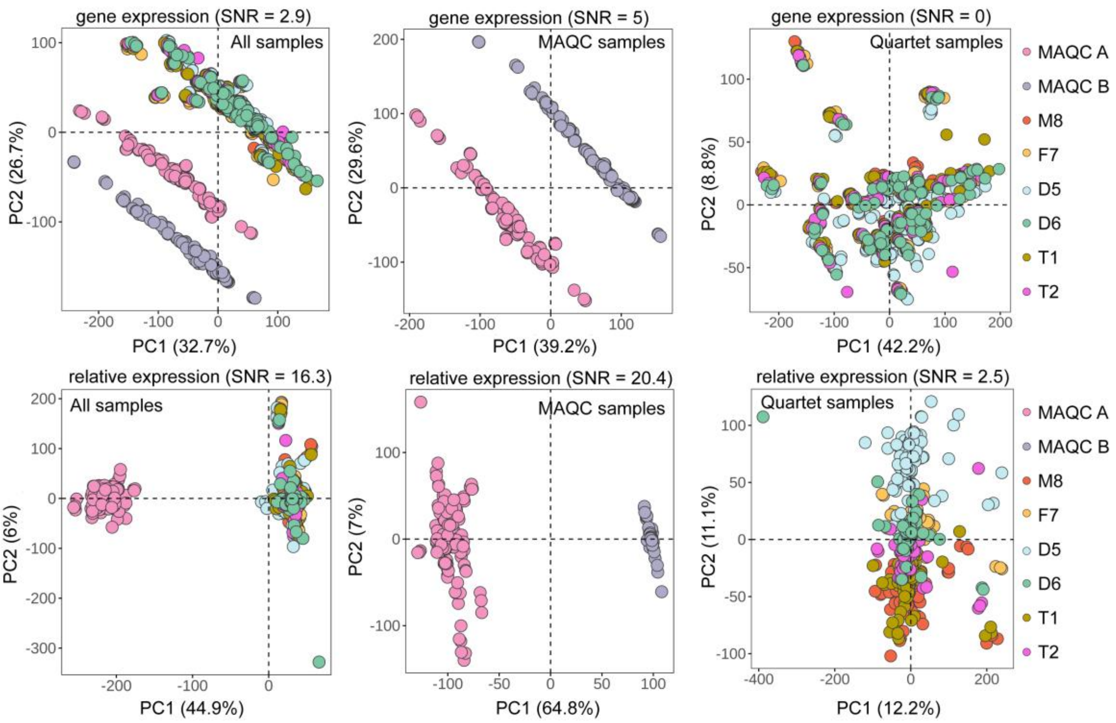

Supplementary Figure 5. PCA by all pooling RNA-seq data for different sample combinations. The MAQC samples exhibit huge biological differences, and performing PCA with the inclusion of MAQC samples can lead to an overestimation of the data quality. Relative expression, as opposed to absolute expression, help distinguish samples from inter-laboratory variations. SNR, signal-to-noise ratio. PCA, Principal component analysis.

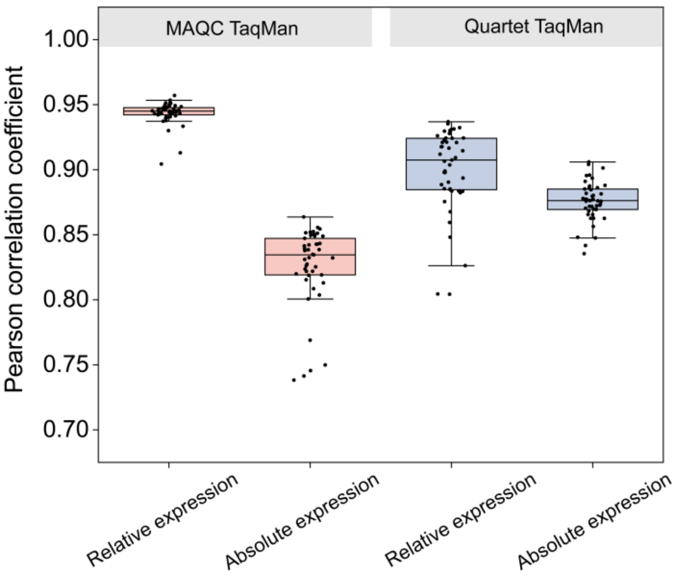

Supplementary Figure 6. Relative expression measurements were more accurate than absolute expression measurements. Based on the TaqMan datasets for both Quartet and MAQC samples, relative expression consistently exhibited higher Pearson correlation coefficients when compared to absolute expression. Box plots present Pearson correlation coefficients for all 45 laboratories, and data are presented as median values (center lines) and the upper and lower quartiles (box limits).

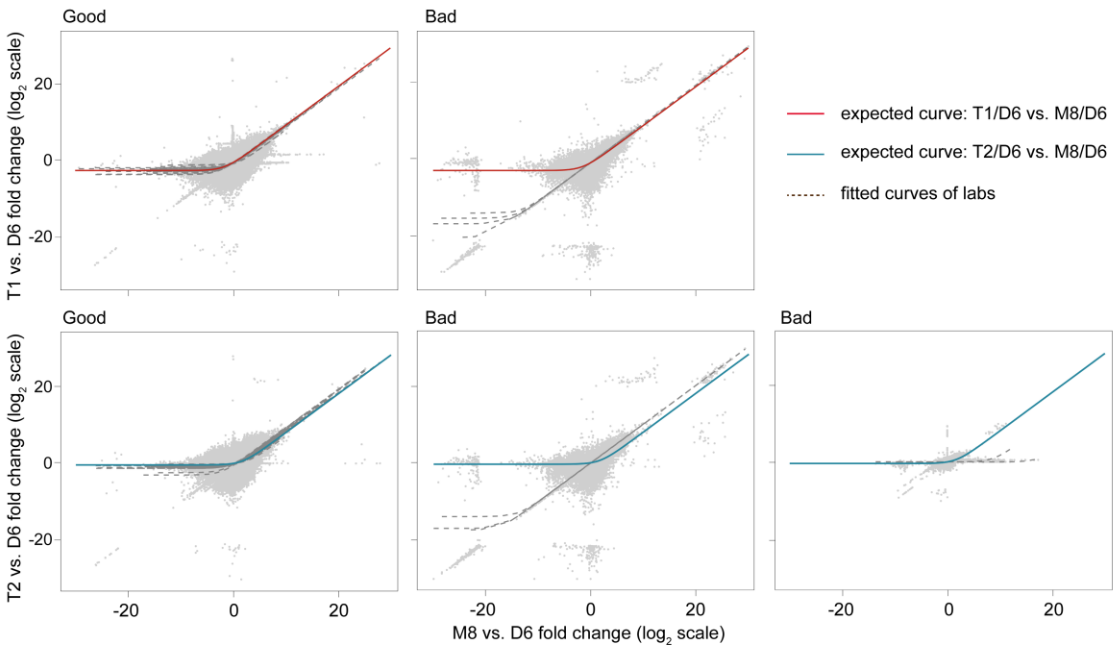

Supplementary Figure 7. Comparison between laboratories with good and bad recovery of mixture ratios of sample T1 and T2. The red and cyan solid line traces the expected curve after mRNA/total-RNA shift correction. The grey dashed lines indicate the fitted curves from data of laboratories. The genes reported by all laboratories are shown in grey. Primarily, the presence of genes with extremely large fold differences resulted in the deviation of the fitting curve. These records were attributed to erroneous calculation of low-expressed genes in individual samples.

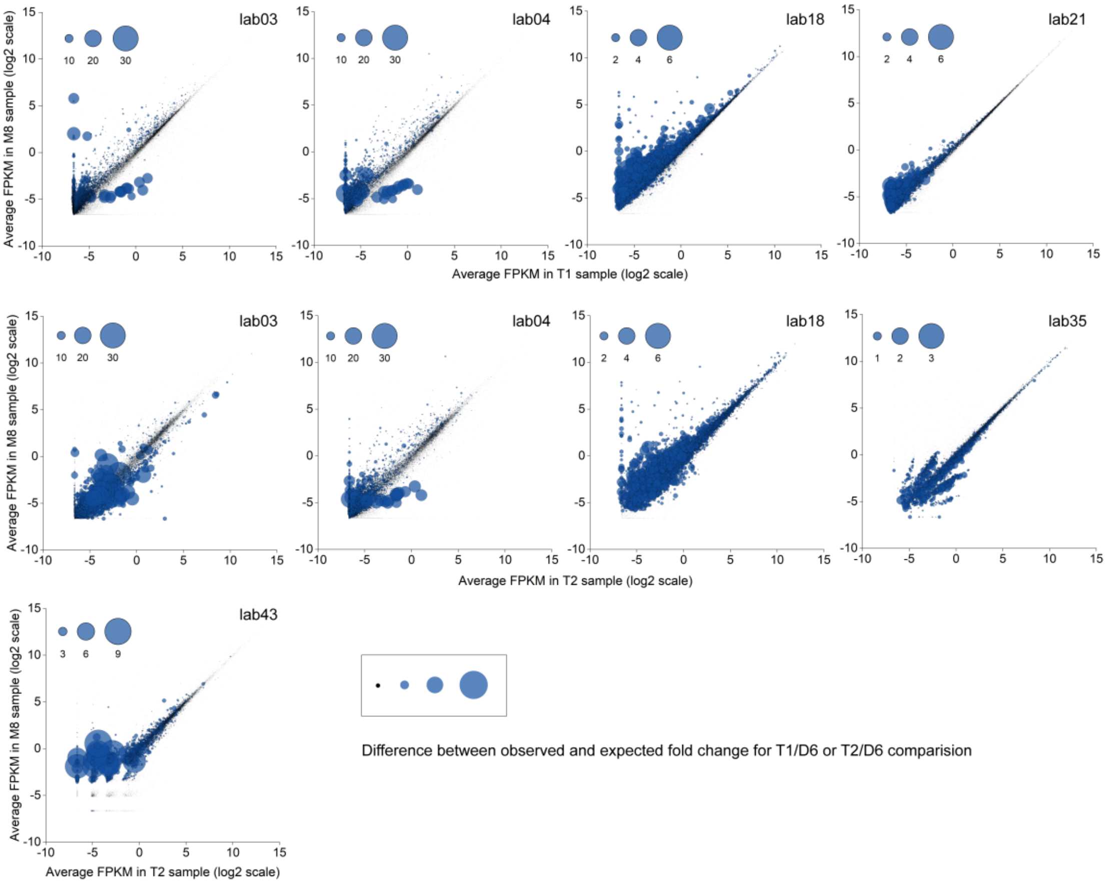

Supplementary Figure 8. Presentation of genes in RNA-seq data with poor recovery of mixed ratios. We focused on four laboratories exhibiting poor recovery of mixing proportions for T1 samples and five laboratories with poor recovery for T2 samples **(Supplementary Figure 7)**. The deviations between the expected and observed relative expression for T1/D6 or T2/D6 comparisons were calculated for all genes, and the impact of gene expression levels on these deviations was analyzed. In the plot, the deviations are represented by the size of circles, where larger circles indicate greater deviations between the expected and observed relative expression for the respective genes. These results indicate that genes with poor recovery of expected mixing ratios (larger circles) tend to exhibit lower expression levels.

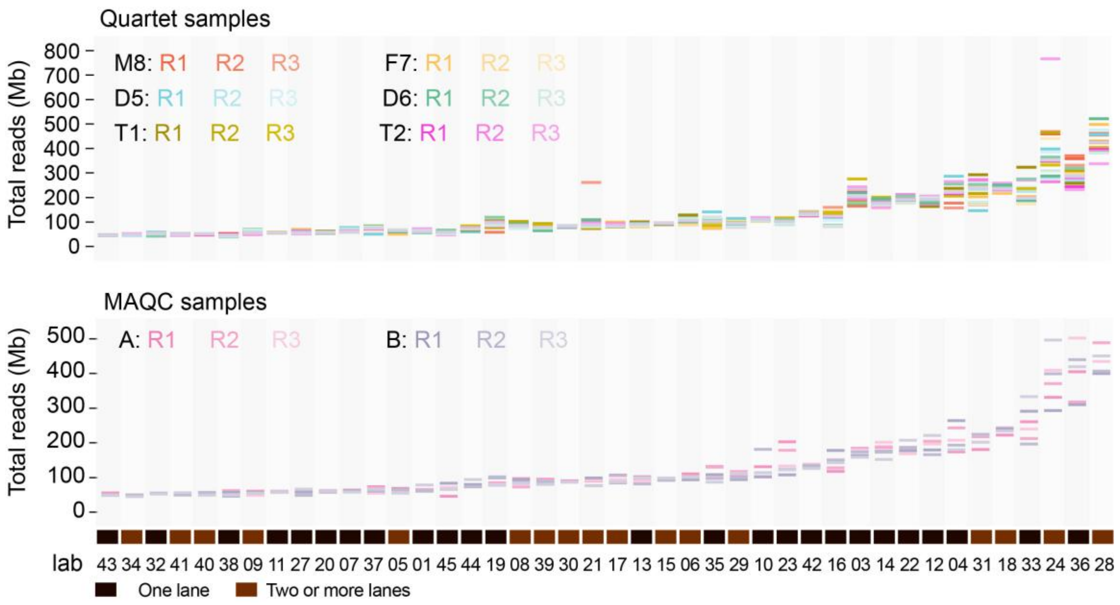

Supplementary Figure 9. The number of reads of RNA-seq data. For all 42 laboratories that submitted complete raw sequencing data, the total number of reads for Quartet (up) and MAQC (down) samples was displayed, where higher sequencing depth is associated with greater inter-sample variations.

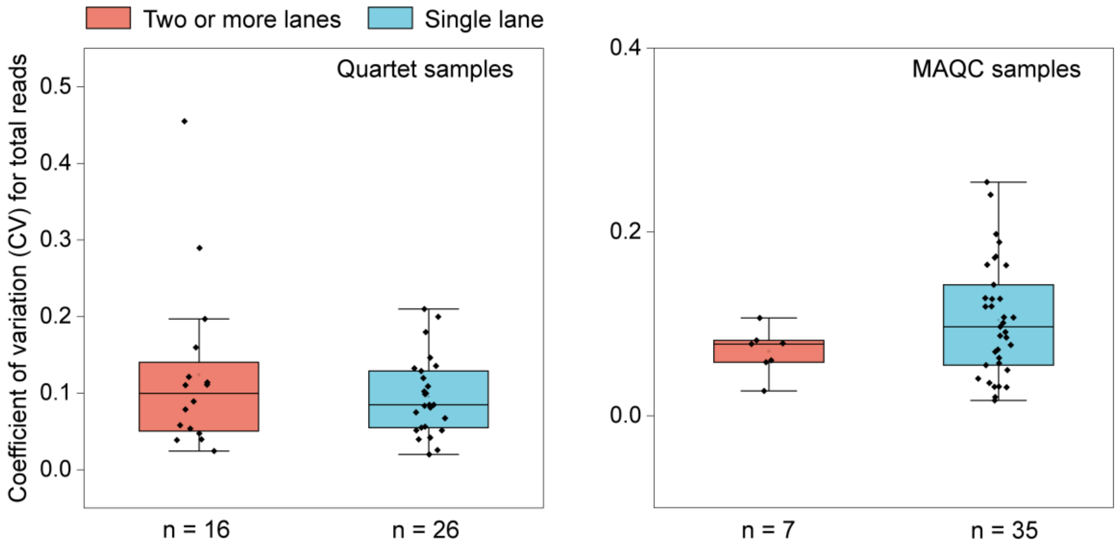

Supplementary Figure 10. The influence of different lanes on the sequencing depth. A total of 16 laboratories assigned the 24 libraries into different lanes, including 16 and 7 laboratories assigning the 18 Quartet and 6 MAQC libraries into different lanes. Assigning into different lanes did not lead to a higher coefficient of variation (CV) for total reads when compared to assigning the libraries into a single lane across the Quartet (left) and MAQC (right) samples. Box plots in red and blue present CV values of sequencing depth for laboratories assigning libraries into different and single lanes, respectively, and data are presented as median values (center lines) and the upper and lower quartiles (box limits).

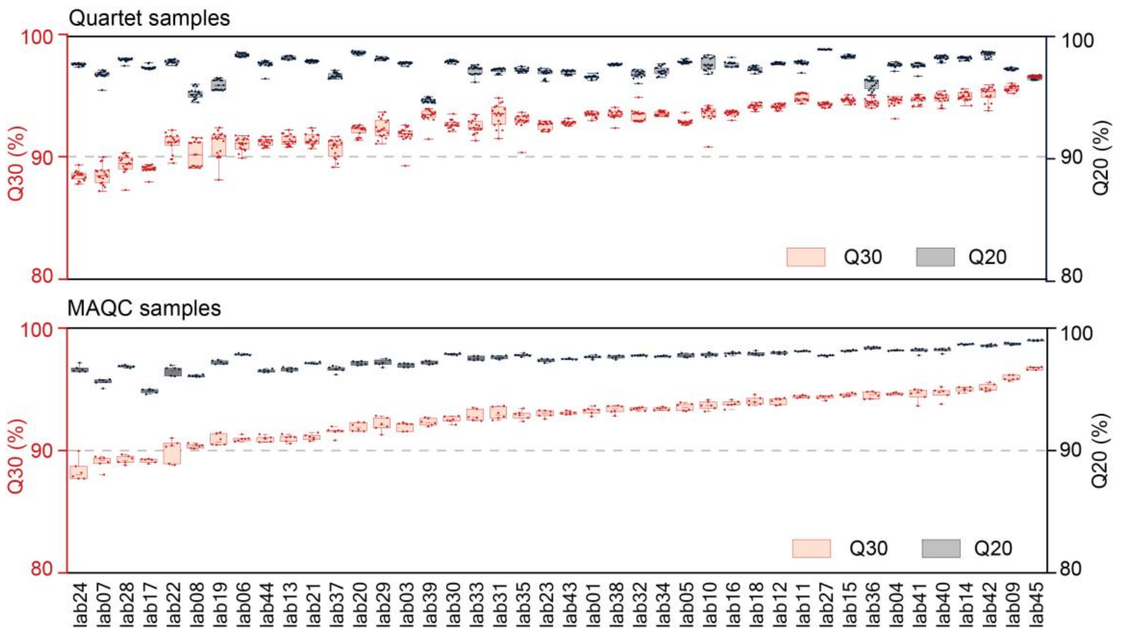

Supplementary Figure 11. Quality values of sequencing reads of RNA-seq data. Q30 for the Quartet (up) and MAQC samples (down) was calculated as the percentage of bases with a quality score of 30 or higher, indicating the base call accuracy is 99.9%. Q20 was calculated as the percentage of bases with a quality score of 20 or higher, indicating the base call accuracy is 99%. Box plots present Q30 and Q20 values for 18 Quartet (up) and 6 MAQC (down) samples, and data are presented as median values (center lines) and the upper and lower quartiles (box limits).

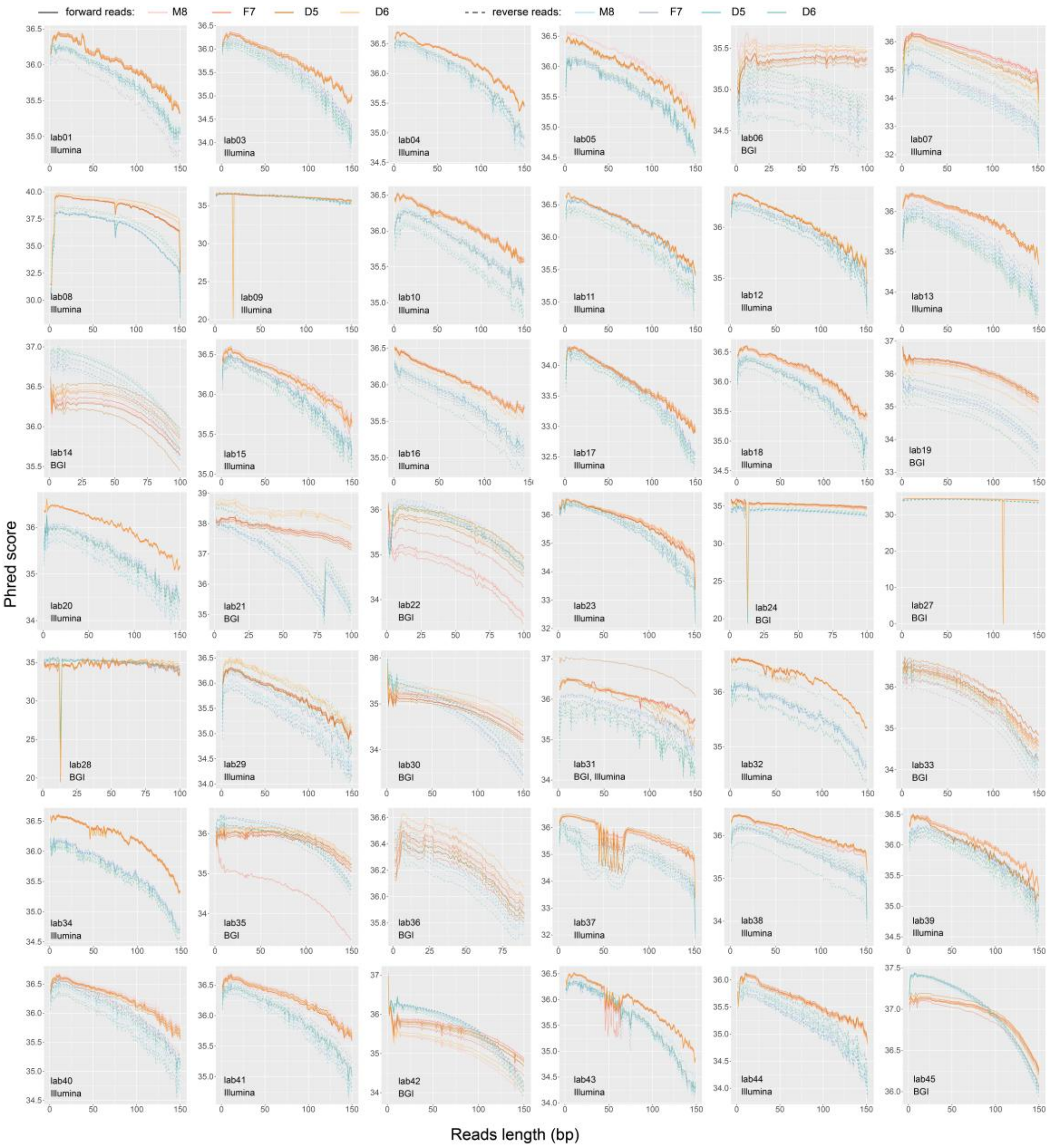

Supplementary Figure 12. Base quality values of representative sequencing read for the Quartet samples. The forward reads refer to the reads firstly extended from the Reads 1 adapter, and the reverse reads refer to the reads secondly extended from the Reads 2 adapter in the sequencing reaction. The base quality distribution in the first 1–10 bases was biased in most laboratories. The quality of reverse reads was generally lower than that of forward reads in most laboratories. Different colors represent different samples, with squares and circles representing forward and reverse reads, respectively.

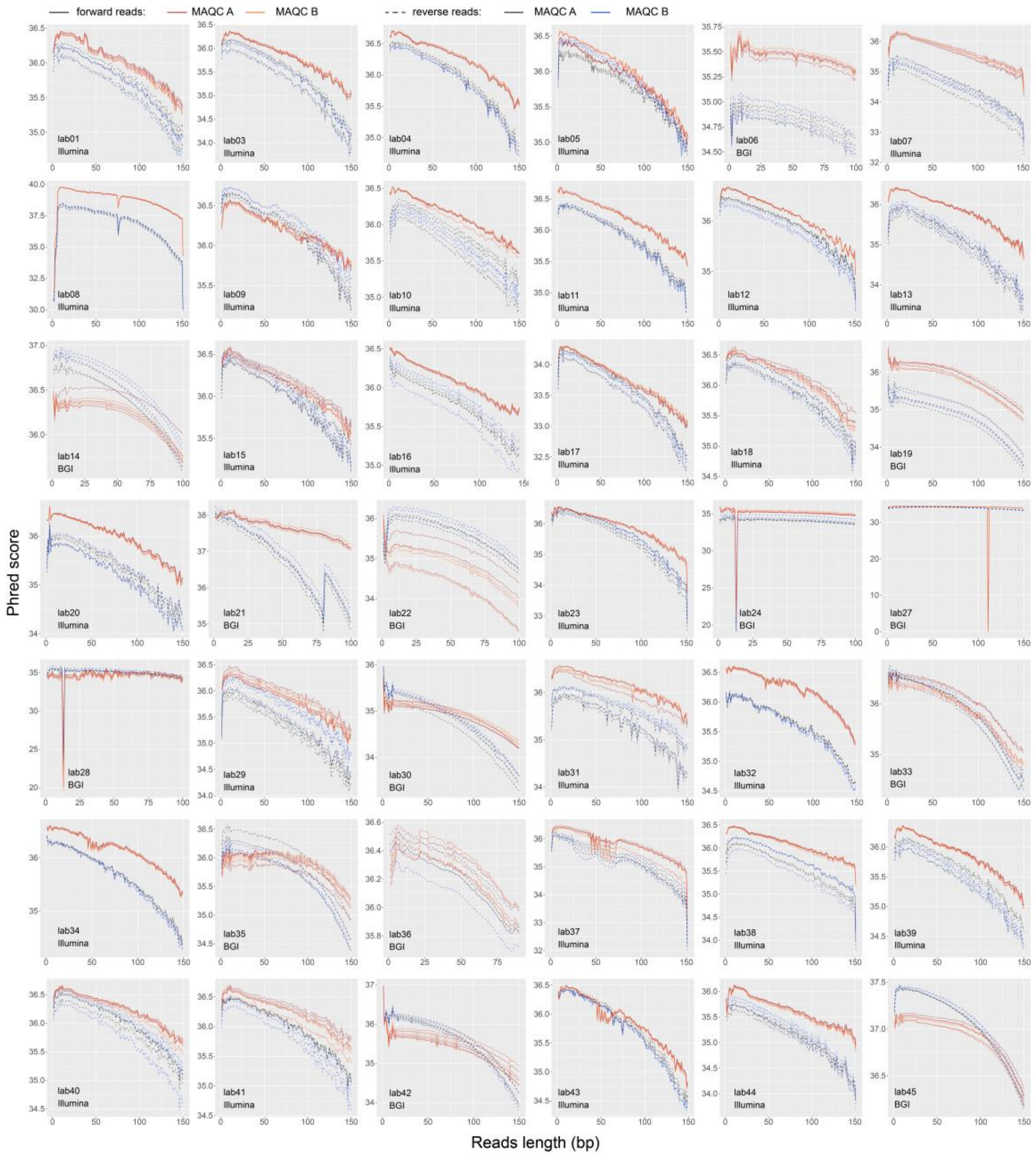

Supplementary Figure 13. Base quality values of representative sequencing read for the MAQC samples. Different colors represent different samples, with squares and circles representing forward and reverse reads, respectively.

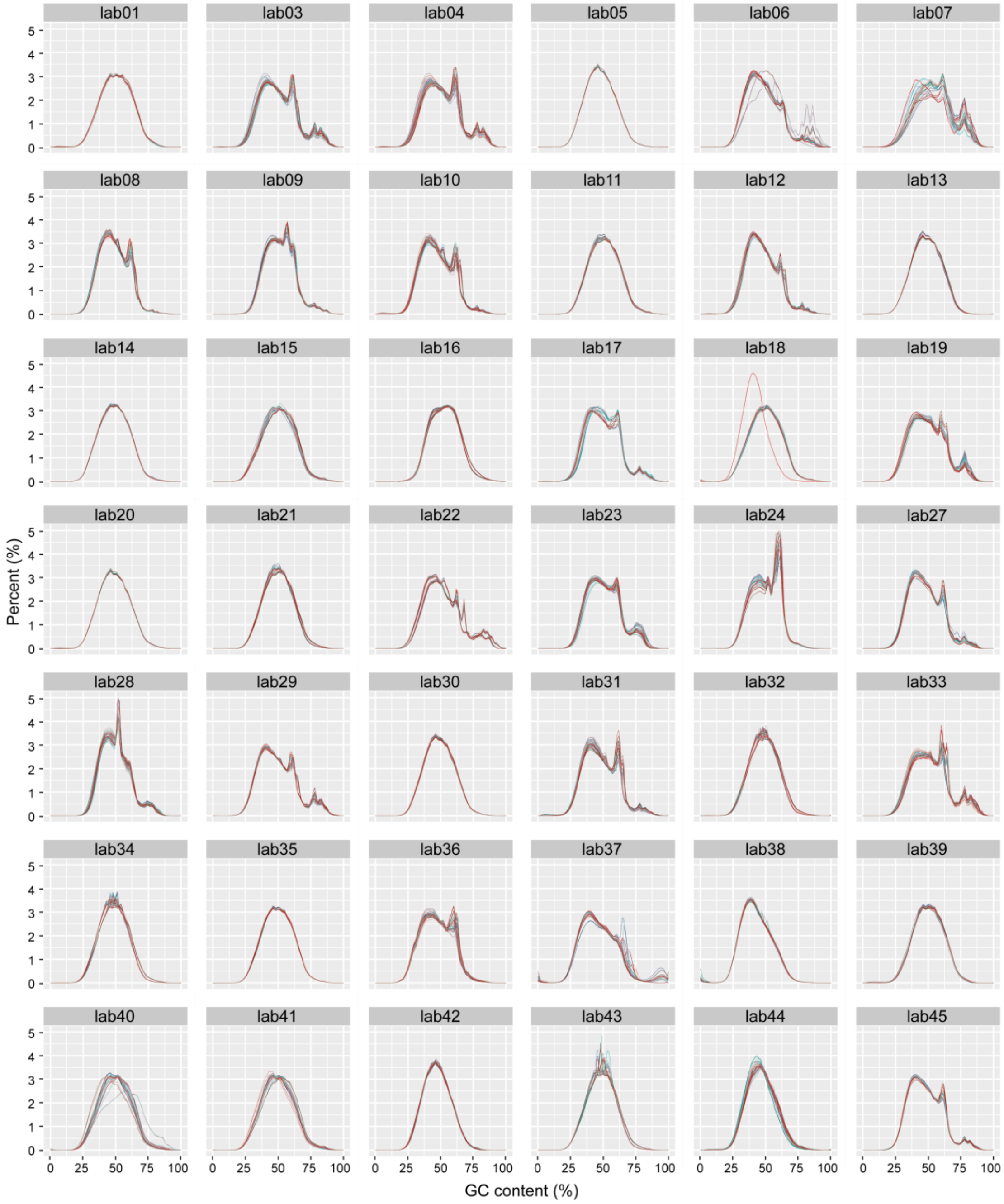

Supplementary Figure 14. GC distribution of RNA-seq data for Quartet samples.

Three replicates of M8, F7, D5, D6, T1, and T2 were included.

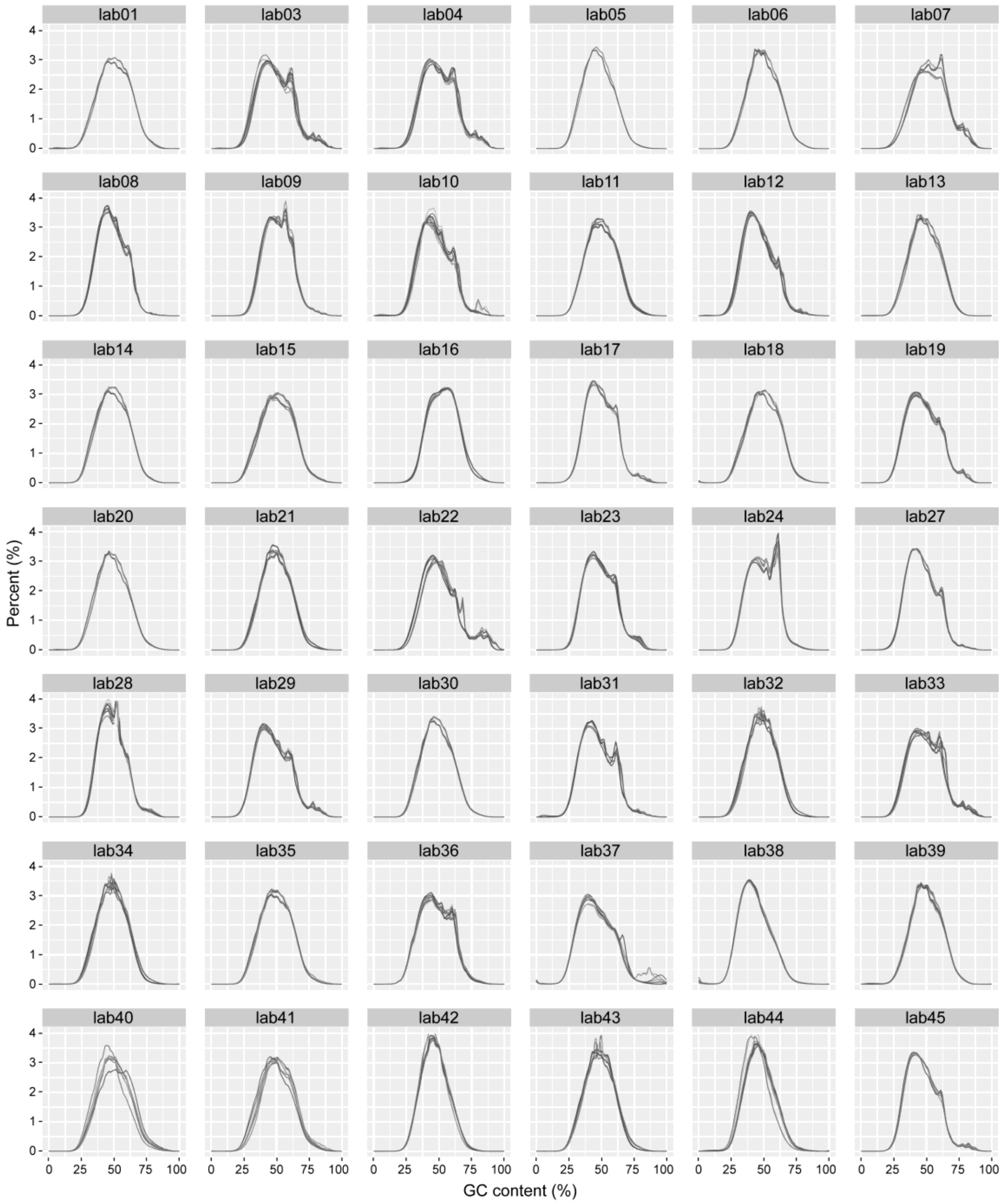

Supplementary Figure 15. GC distribution of RNA-seq data for MAQC samples.

Three replicates of MAQC A and B were included.

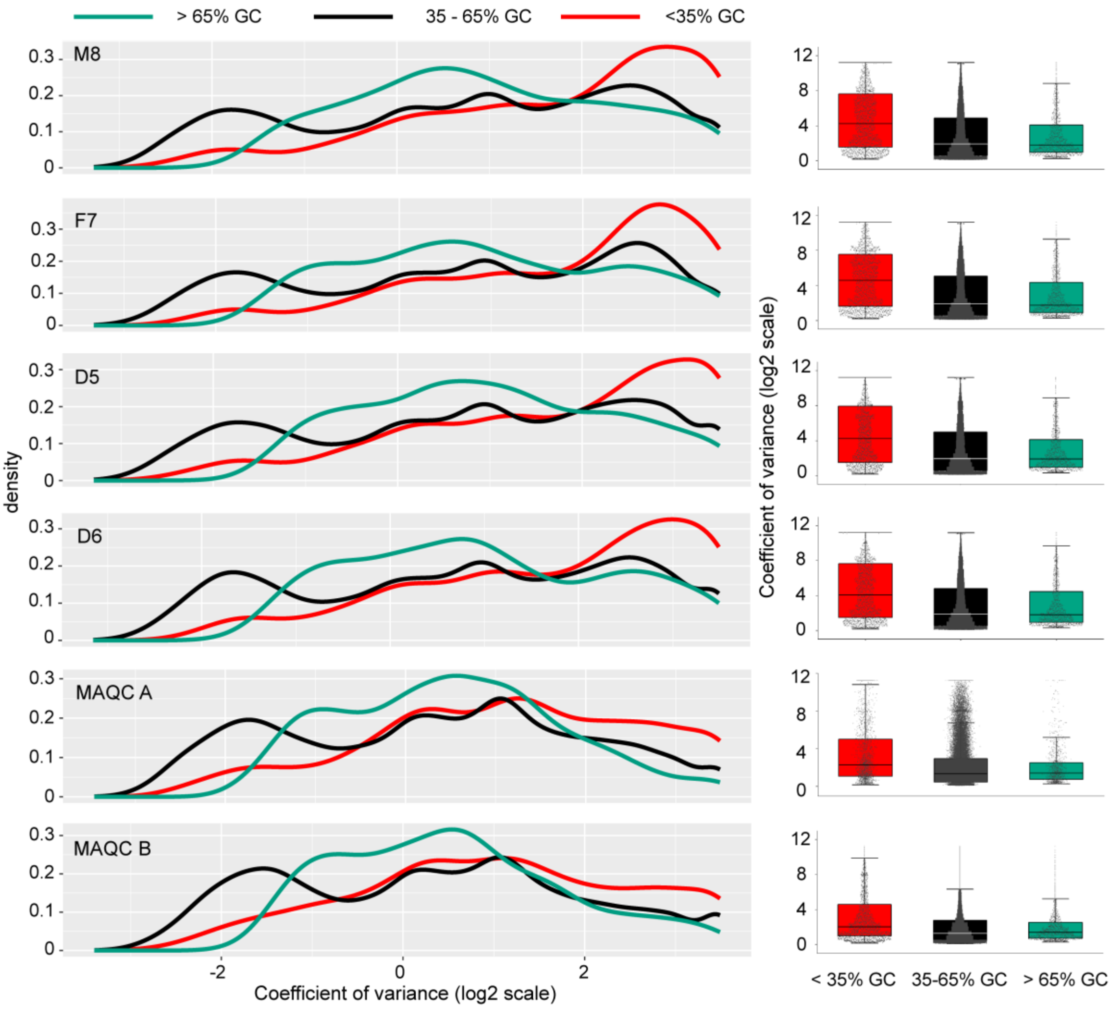

Supplementary Figure 16. Impact of GC content on inter-laboratory agreement of gene expression. Low GC content is associated with high inter-laboratory variations of gene expression measurement. Box plots present coefficient of variation (CV) values of expression levels across 42 laboratories for genes with GC contents of <35% (n = 2,118), 35-65% (n = 56,161), and >65% (n = 1,185), and data are presented as median values (center lines) and the upper and lower quartiles (box limits).

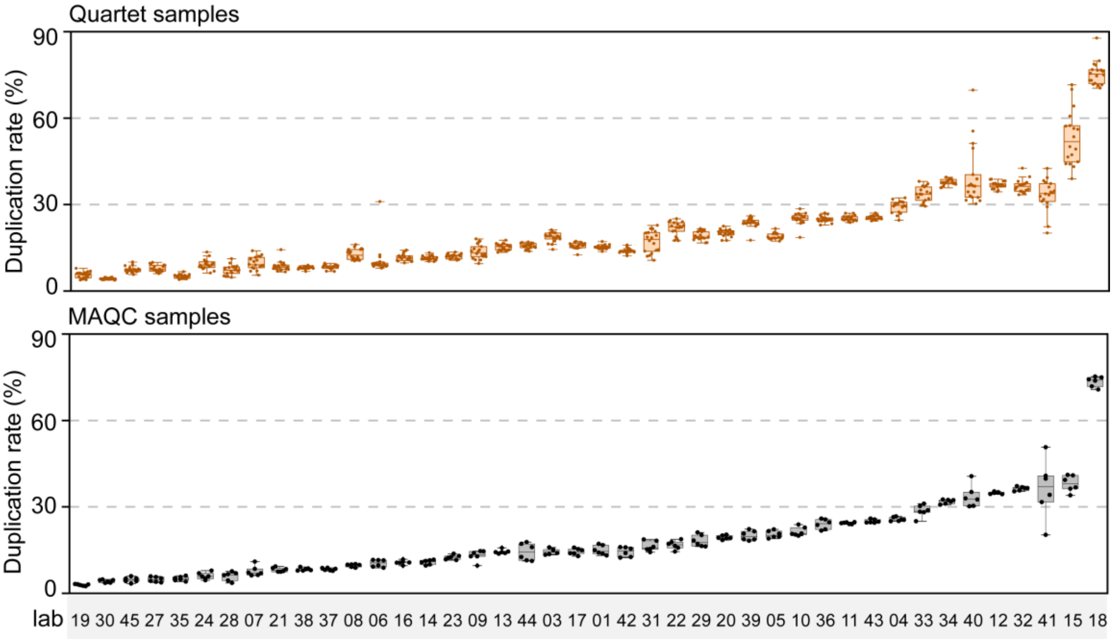

Supplementary Figure 17. The duplication rate of RNA-seq data. The duplication rate of sequencing reads in Quartet (up) and MAQC (down) samples was calculated using fastp (v.0.23.2)[1]. Previous studies have suggested that the duplication rate is typically below 30%[2-4]. Box plots present duplication rates for 18 Quartet (up) and 6 MAQC (down) samples, and data are presented as median values (center lines) and the upper and lower quartiles (box limits).

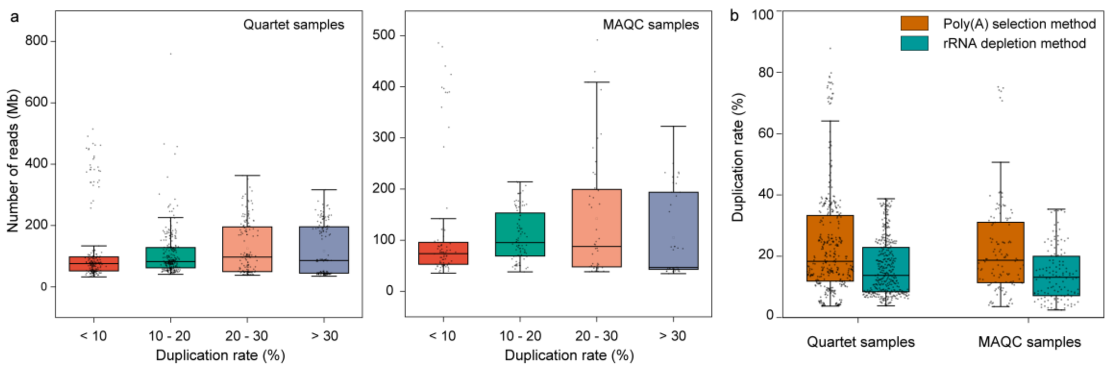

Supplementary Figure 18. The correlation between duplication rates and sequencing depth and the mRNA enrichment methods. The duplication rate of sequencing reads was estimated based on FASTQ files using fastp (v.0.23.2)[1]. **(a)** The RNA-seq data for all Quartet (left) and MAQC (right) from 42 laboratories were categorized into four subgroups according to duplication rates: <10% (n = 197 and n = 76 for the Quartet and MAQC samples), 10%–20% (n = 197 and n = 76 for the Quartet and MAQC samples), 10%–20% (n = 148 and n = 44 for the Quartet and MAQC samples), and >30% (n = 147 and n = 43 for the Quartet and MAQC samples). The Quartet and MAQC samples with higher duplication rates showed increased sequencing depth. **(b)** For the Quartet (n = 378) and MAQC samples (n = 126) for laboratories using different mRNA enrichment methods, samples using the Poly(A) selection method demonstrated higher duplication rates compared to samples using the rRNA depletion method. Data in box plots are presented as median values (center lines) and the upper and lower quartiles (box limits).

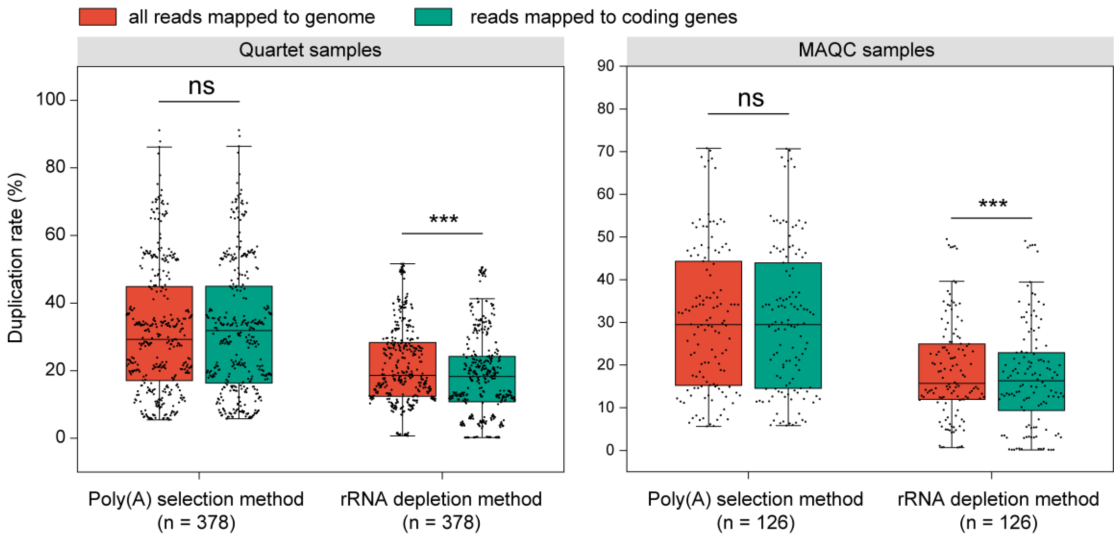

Supplementary Figure 19. The duplication rate of reads mapped to coding genes. The duplication rate of reads mapped to coding genes and all reads mapped to the genome was estimated based on BAM files using the Picard CollectInsertSizeMetrics function (v.3.1.1)[5]. For both Quartet (n = 378) and MAQC (n = 126) samples for laboratories using different mRNA enrichment methods, the Poly(A) selection method exhibited higher duplication rates of reads mapped to coding genes compared to those of reads mapped to the genome. Data in box plots are presented as median values (center lines) and the upper and lower quartiles (box limits). The significance was tested using paired t-test. *** indicates a two-sided *p-value* < 0.001. ns, not significant.

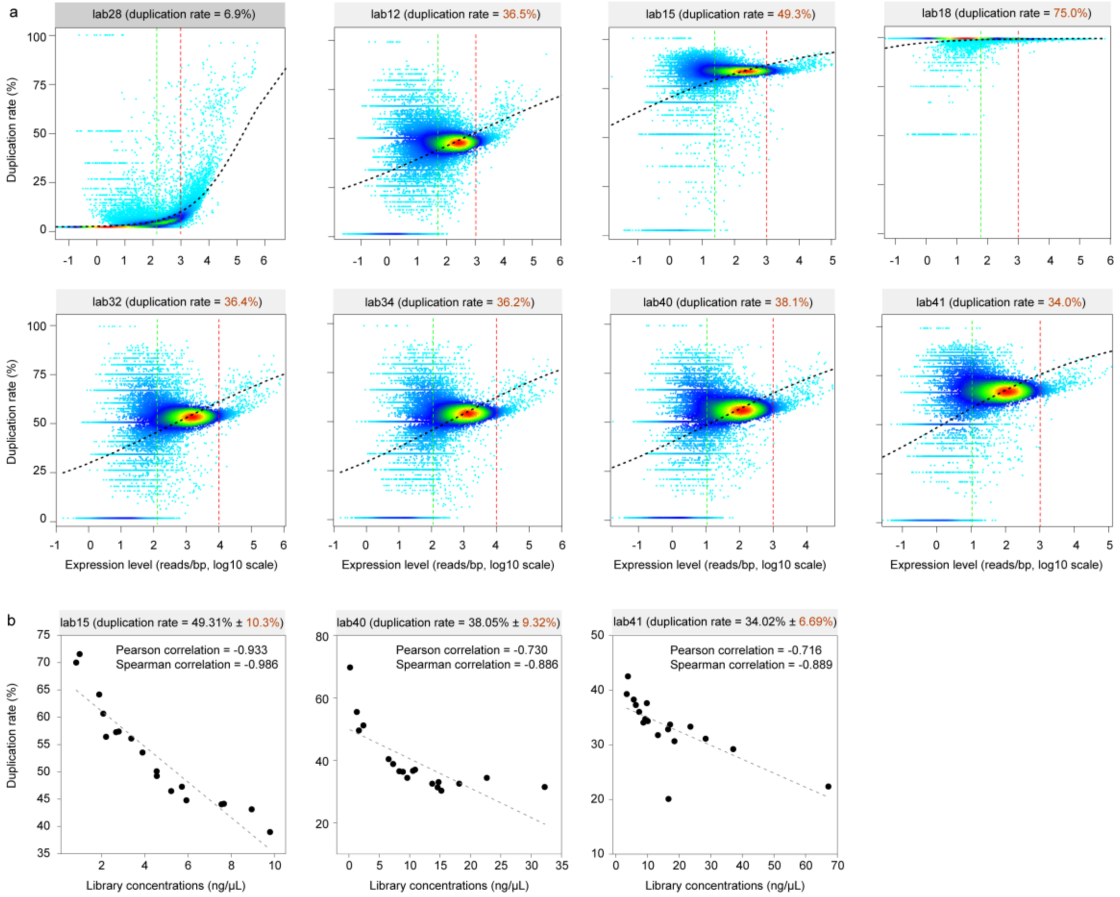

Supplementary Figure 20. Distribution of abnormal duplication rates across laboratories and samples. (a) The dupRadar (v.3.18) tool was used to examine the sources of duplicated reads[6]. RNA-seq data from seven laboratories with high duplication rates (>30%) were included, and data from lab28 with a low duplication rate were utilized for comparison. The figure depicts the distribution of read duplication rates across different expression levels for the Quartet M8 sample. The red dashed line represents the cutoff of one read per base pair for high expression levels, while the green dashed line represents the cutoff of 0.5 FPKM for low expression levels. **(b)** The correlation between duplication rates and library concentrations in the three laboratories shows high standard deviations in duplication rates for 24 samples.

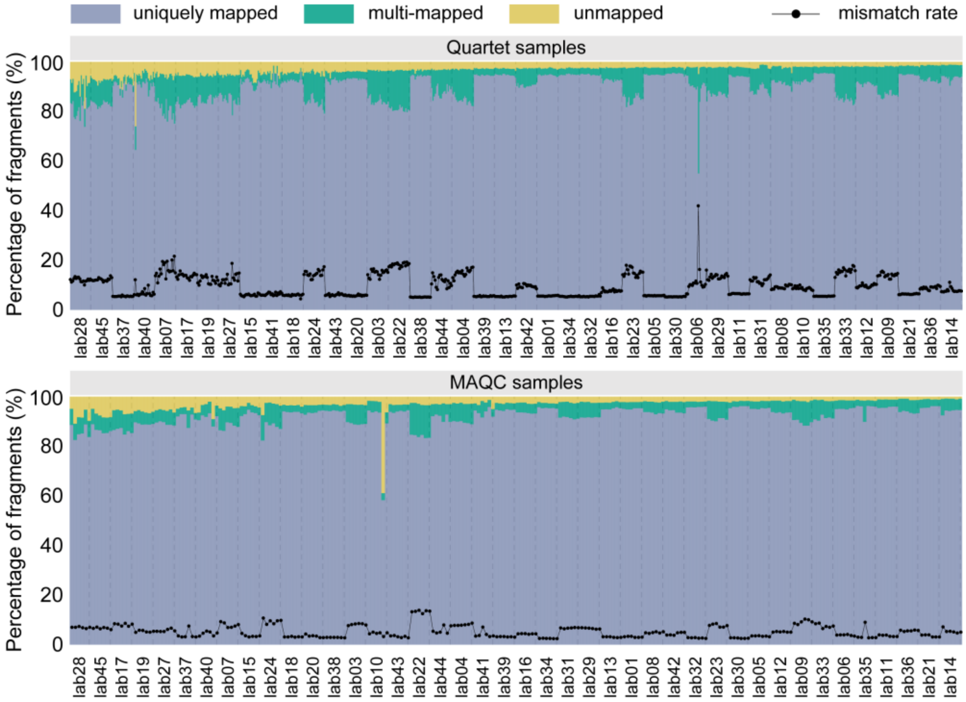

Supplementary Figure 21. The mapping statistic for all RNA-seq data. Raw data of Quartet (up) and MAQC (down) samples was mapped to reference genome using STAR[7]. The black dots indicate the mismatch rate.

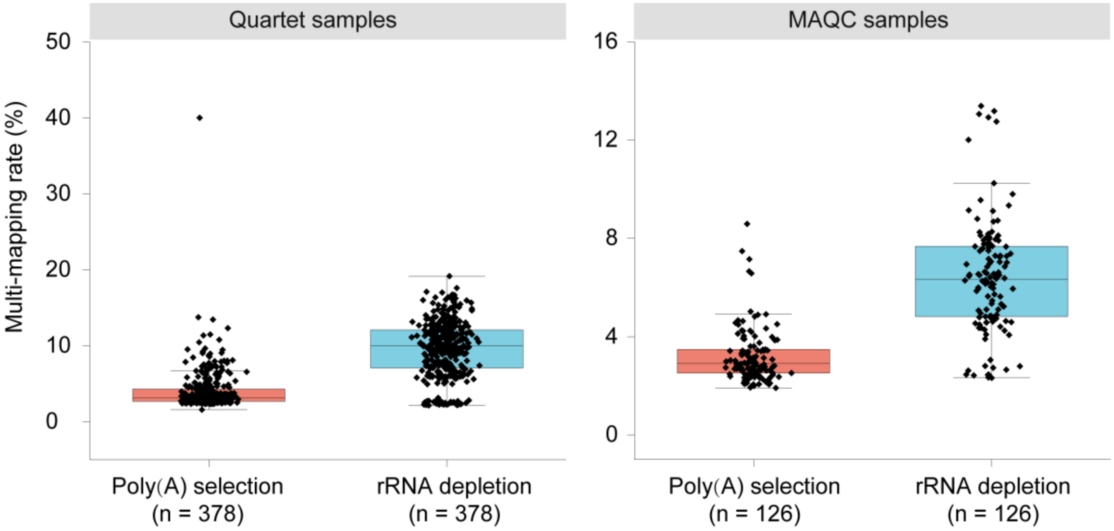

Supplementary Figure 22. The influence of mRNA enrichment methods on multi-mapping rate. Raw data of Quartet (left) and MAQC (right) samples was mapped to reference genome using STAR[7]. Box plots present multi-mapping rates for Quartet (n = 378) and MAQC (n = 126) samples for laboratories using different mRNA enrichment methods, and data are presented as median values (center lines) and the upper and lower quartiles (box limits). The rRNA depletion methods corresponding to a high multi-mapping rate.

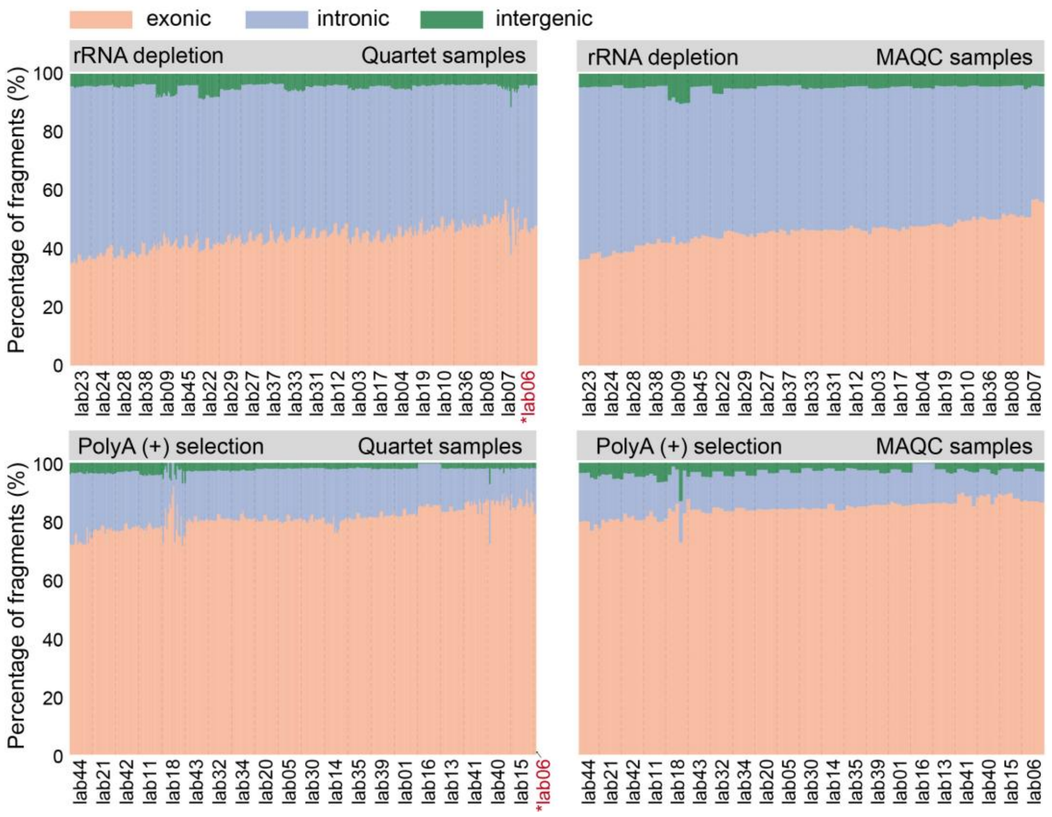

Supplementary Figure 23. Percentage of mapped reads in exonic, intronic, and intergenic regions. For both the Quartet and MAQC samples, the Poly(A) selection protocol is consistently associated with a higher percentage of reads in the exonic regions. For lab06, the libraries for the MAQC samples and two Quartet M8 replicates (R1 and R2) were prepared using the Poly(A) selection method, while the remaining Quartet samples (one M8 replicate and F7, D5, and D6), as well as the mixed samples T1 and T2, were all prepared using the rRNA depletion method. The two subgroups of samples were sequenced in two different runs on the same sequencing instrument. * indicates that the Quartet samples were processed using two different mRNA enrichment methods in lab06.

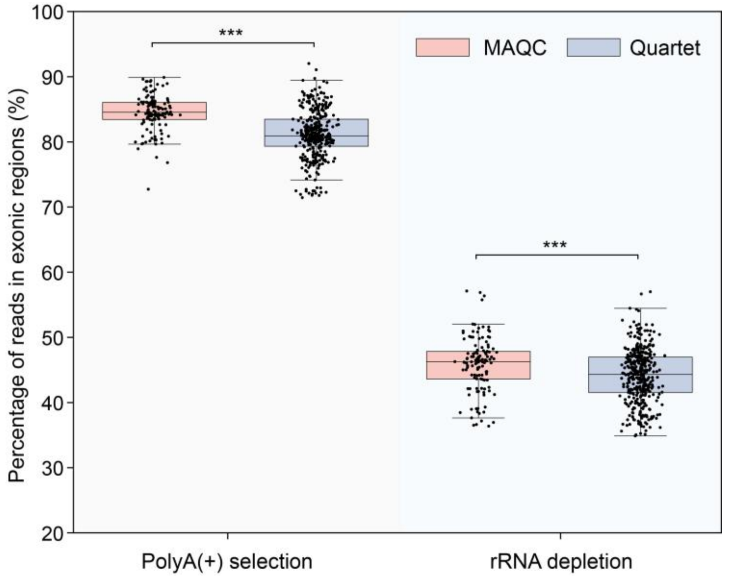

Supplementary Figure 24. Percentage of the exonic reads. Box plots present percentage of reads mapped to the exonic regions for Quartet (n = 378) and MAQC (n = 126) samples, and data are presented as median values (center lines) and the upper and lower quartiles (box limits). There are significant higher number of reads mapped to the exonic regions in the MAQC samples than the Quartet samples for both the Poly(A) selection method and the rRNA depletion method. This may be attributed to more highly expressed genes in the MAQC samples. *** indicates a two-sided *p-value* < 0.001. The significance was tested using unpaired t-test.

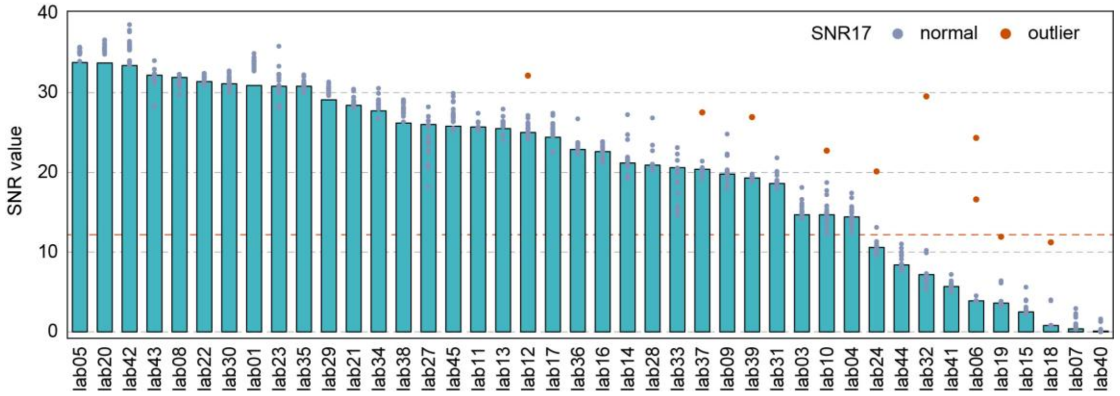

Supplementary Figure 25. SNR after applying the fixed data analysis pipeline. The expression data of 42 laboratories were obtained using the Ensembl-STAR-StringTie pipeline to eliminate the effects of different bioinformatics workflows used by the laboratories. Laboratories are ordered by SNR values. Dots represent SNR values based on any 17 of the 18 samples (12 Quartet and 6 mixed samples) in each laboratory. A dot in dark red represents the SNR17 value that increases over six decibels compared to its standard SNR (18-sample SNR), when one sample in this laboratory is excluded, while a dot in cyan represents the SNR17 value that decreases or increases less than six decibels compared to its standard SNR. The red dashed line represents the cutoff of SNR at 12.

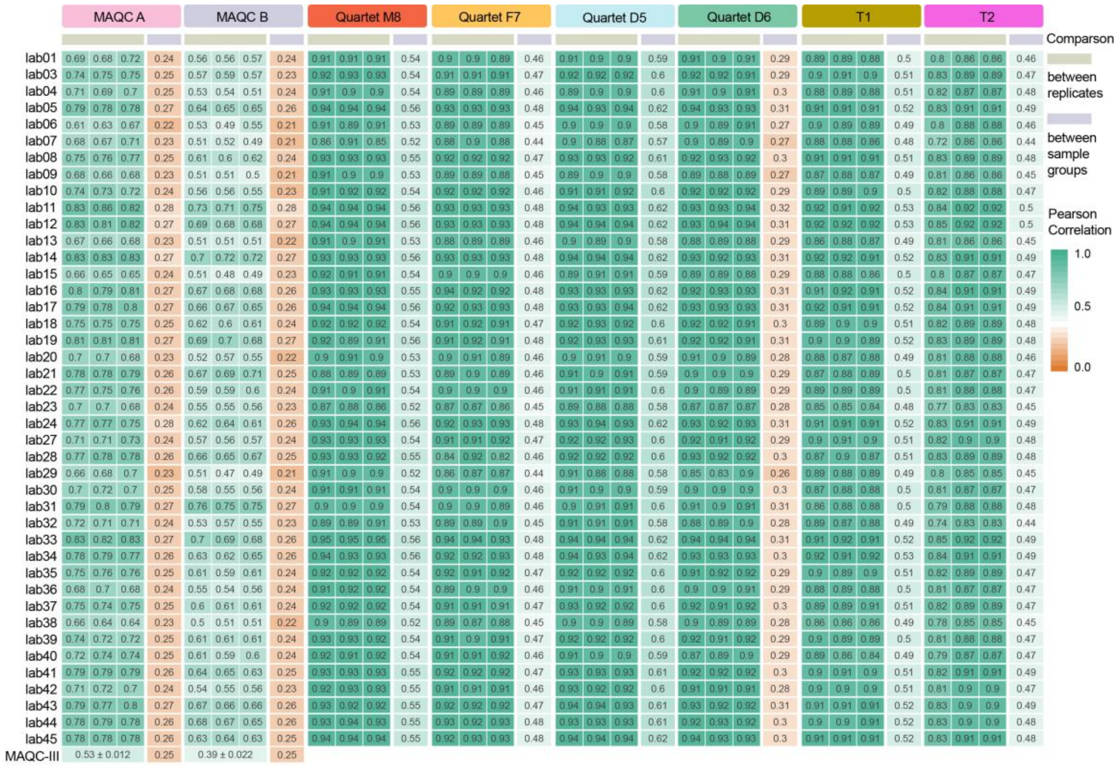

Supplementary Figure 26. SNP-based sample-identity check across laboratories. The Pearson correlation in variant allele frequencies of single nucleotide polymorphisms (SNPs) were calculated between any two out of three replicates and between sample groups using NGSCheckMate (v. 1.0.1)[8]. Four batches of RNA-seq data for MAQC samples A and B from the MAQC-III study were obtained from GEO (series accession number: GSE47792).

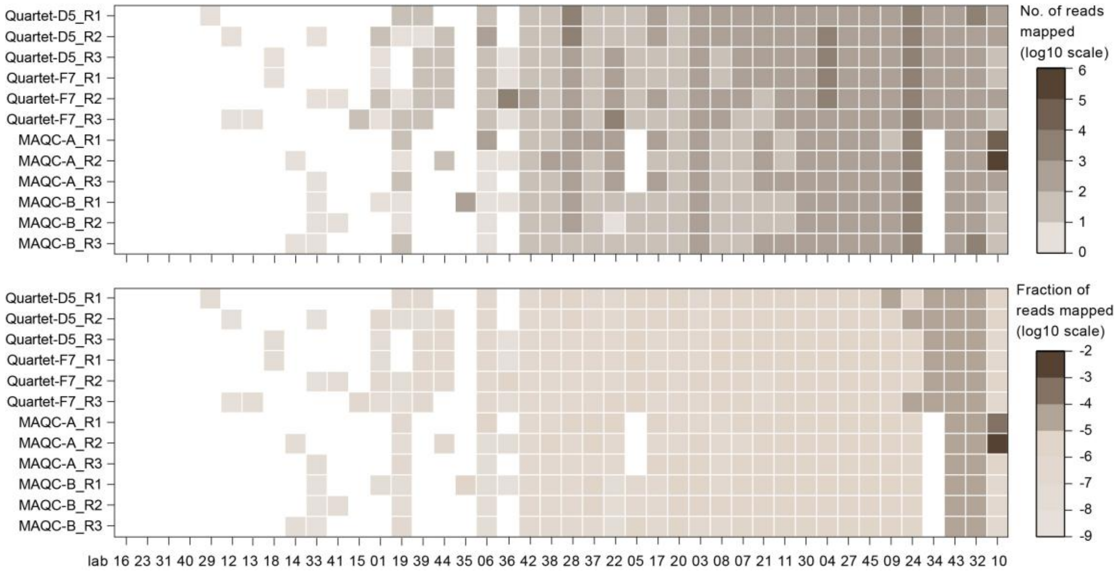

Supplementary Figure 27. Cross-contamination assessment based on ERCC controls. In replicates of MAQC A, B, D5, and F7 samples, reads aligned to the ERCC genes should not be present, offering a valuable opportunity to assess potential cross-contamination.

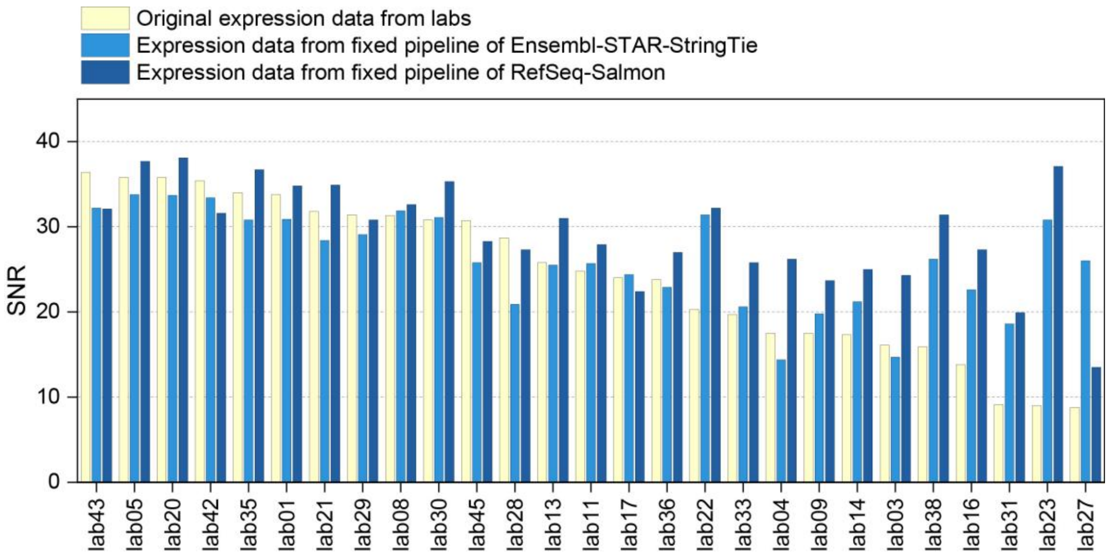

Supplementary Figure 28. Data quality (SNR values) after applying fixed analysis pipelines. The two gene quantification pipelines (Ensembl-STAR-StringTie and RefSeq-Salmon) were applied for RNA-seq data from 26 laboratories. SNR based on Quartet and mixing samples was compared across expression data submitted from laboratories and from fixed quantification pipelines. The variations in SNR values across laboratories significantly decreased after using fixed pipelines, indicating efficient elimination of bioinformatics noises. More than half of the laboratories demonstrated an increased SNR after applying the uniformed analysis pipeline, especially for those with initially lower SNR values, indicating the failure of bioinformatics pipelines in these laboratories. However, some laboratories experienced a decrease in SNR values after applying the uniformed analysis pipeline. This observation may be related to the different number of genes considered, as the uniformed analysis pipeline encompassed all genes with non-zero read counts, whereas real laboratories included a smaller gene set due to their gene type preferences and the selection of gene annotation and analysis tools. SNR, signal-to-noise ratio.

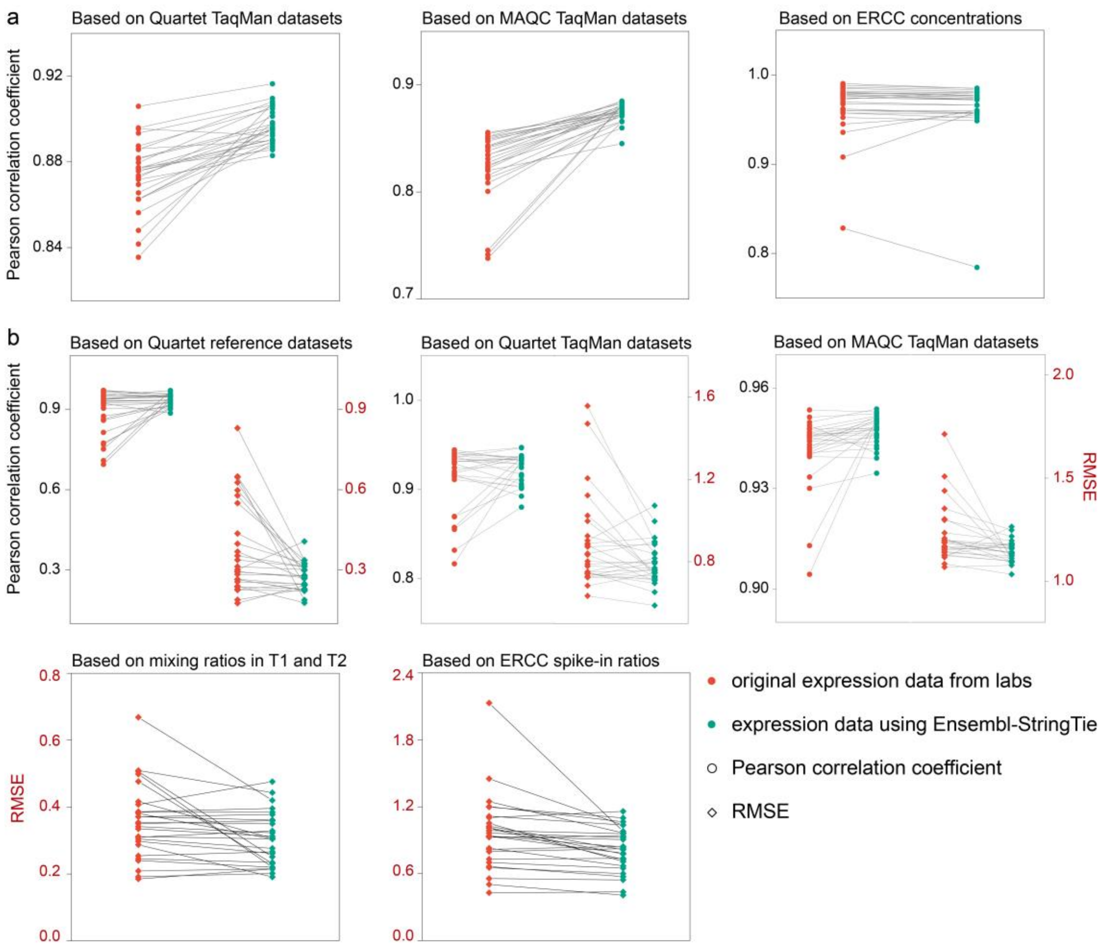

Supplementary Figure 29. The accuracy of absolute and relative expression after applying the fixed analysis pipeline (Ensembl-STAR-StringTie). RNA-seq data from 26 laboratories that passed QC metrics were included. The accuracy between expression data submitted from laboratories and those obtained using Ensembl annotation, STAR for alignment, and StringTie for gene quantification, was compared. **(a)** The accuracy of absolute gene expression was assessed based on the TaqMan datasets for Quartet and MAQC samples, and ERCC concentrations. **(b)** The accuracy of relative gene expression was assessed based on the Quartet reference datasets, the TaqMan datasets for Quartet and MAQC samples, and built-in truth (ERCC spike-in ratios and mixing ratios in T1 and T2). After applying the analysis pipeline, the variations in accuracy metrics decreased, including Pearson correlation coefficients and RMSE. Some laboratories demonstrated improved accuracy when using the fixed pipeline. The red dots represent the metric calculated from the data submitted by laboratories, whereas the cyan dots indicate the metric after applying the fixed analysis pipeline. The circles indicate the Pearson correlation coefficient between laboratories and reference datasets, and the diamonds indicate the Root Mean Square Error (RMSE).

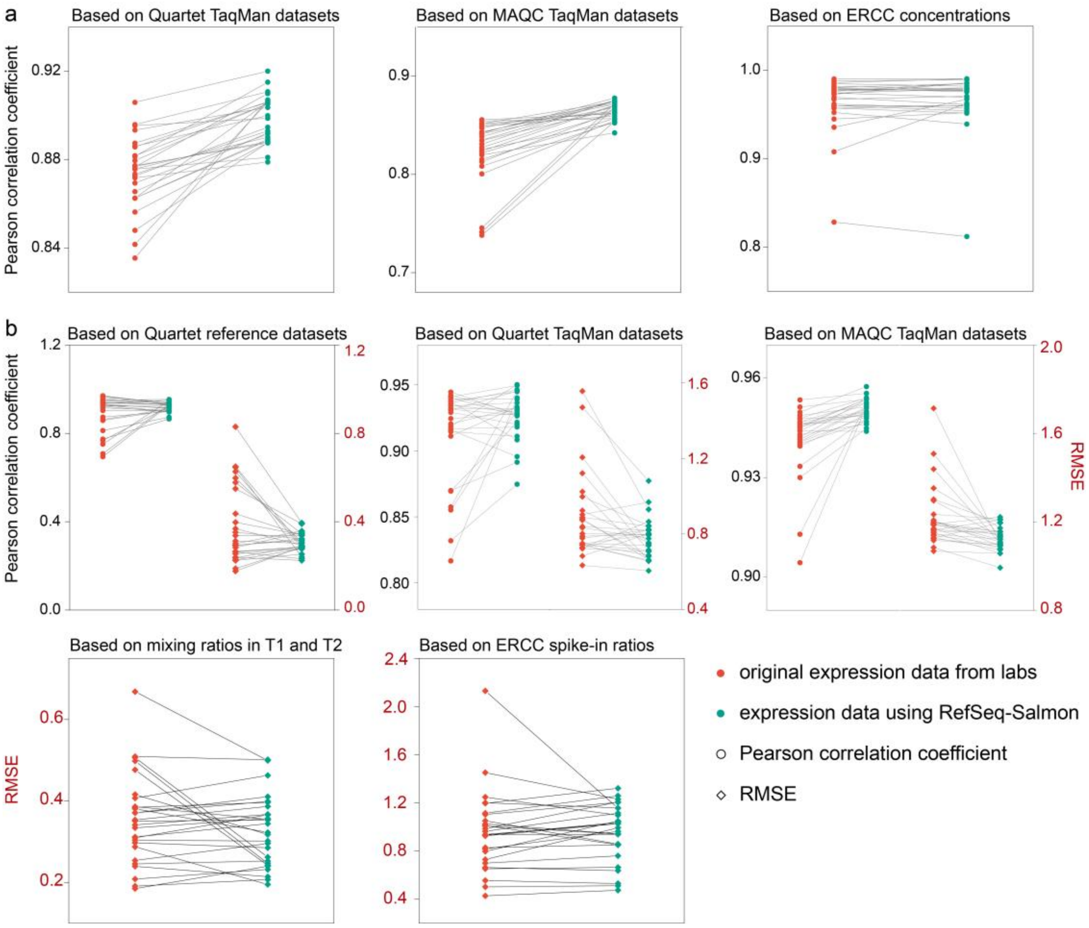

Supplementary Figure 30. The accuracy of absolute and relative expression after applying the fixed analysis pipeline (RefSeq-Salmon). RNA-seq data from 26 laboratories that passed QC metrics were included. The accuracy between expression data submitted from laboratories and those obtained using RefSeq annotation and Salmon for gene quantification, was compared. **(a)** The accuracy of absolute gene expression was assessed based on the TaqMan datasets for Quartet and MAQC samples, and ERCC concentrations. **(b)** The accuracy of relative gene expression was assessed based on the Quartet reference datasets, the TaqMan datasets for Quartet and MAQC samples, and built-in truth (ERCC spike-in ratios and mixing ratios in T1 and T2). Applying RefSeq-Salmon across laboratories also led to decreased variations in accuracy, indicating that the variations introduced from different bioinformatics pipelines were effectively mitigated.

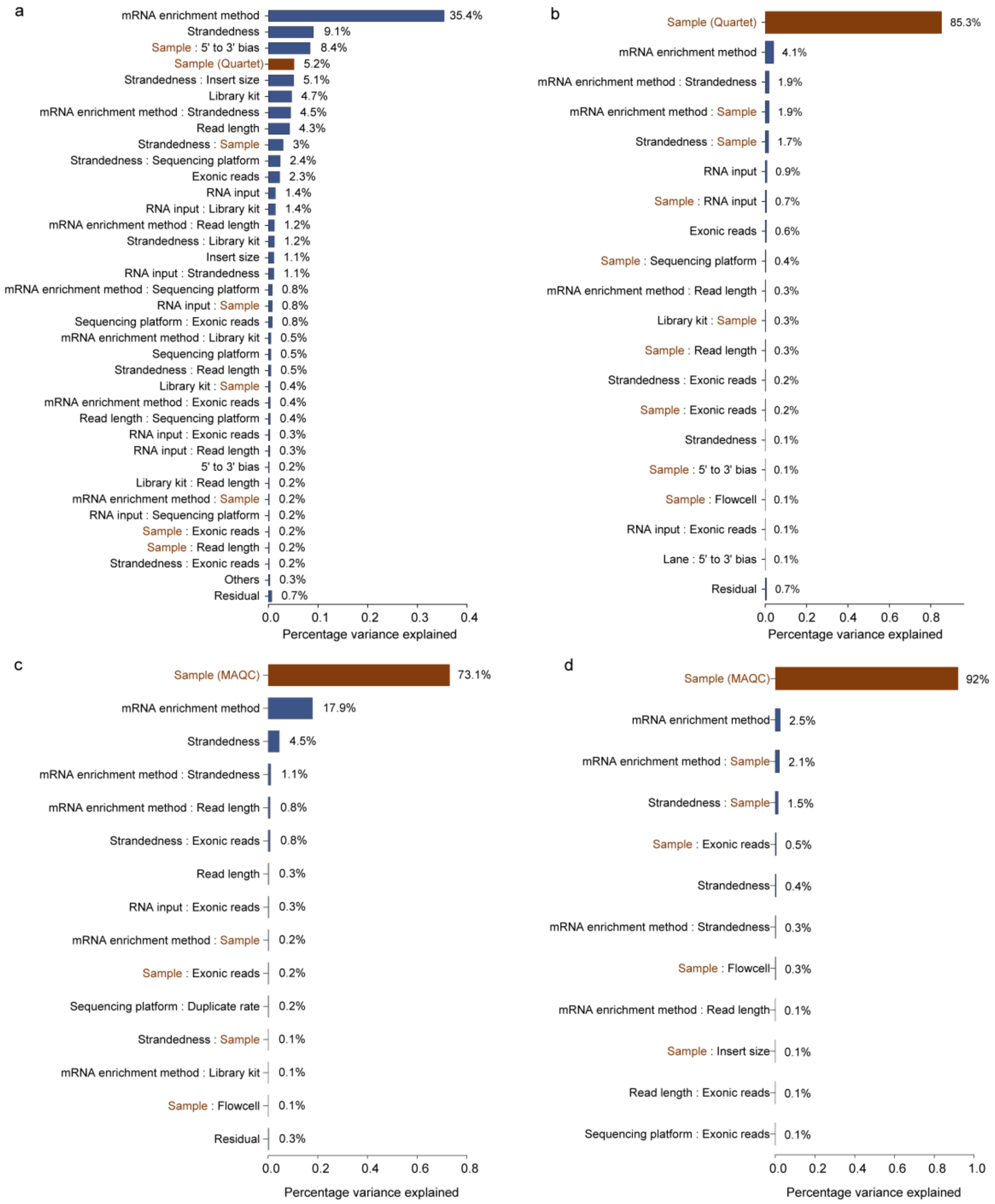

Supplementary Figure 31. PVCA of variations in absolute and relative based on different pipeline (RefSeq-Salmon). Principal variance component analysis quantifies the proportions of variance explained by each experimental factor in the **(a)** absolute expression levels and **(b)** relative expression levels for the Quartet samples. The proportions of variance explained by each experimental factor in the **(c)** absolute expression levels and **(d)** relative expression levels for the MAQC samples.

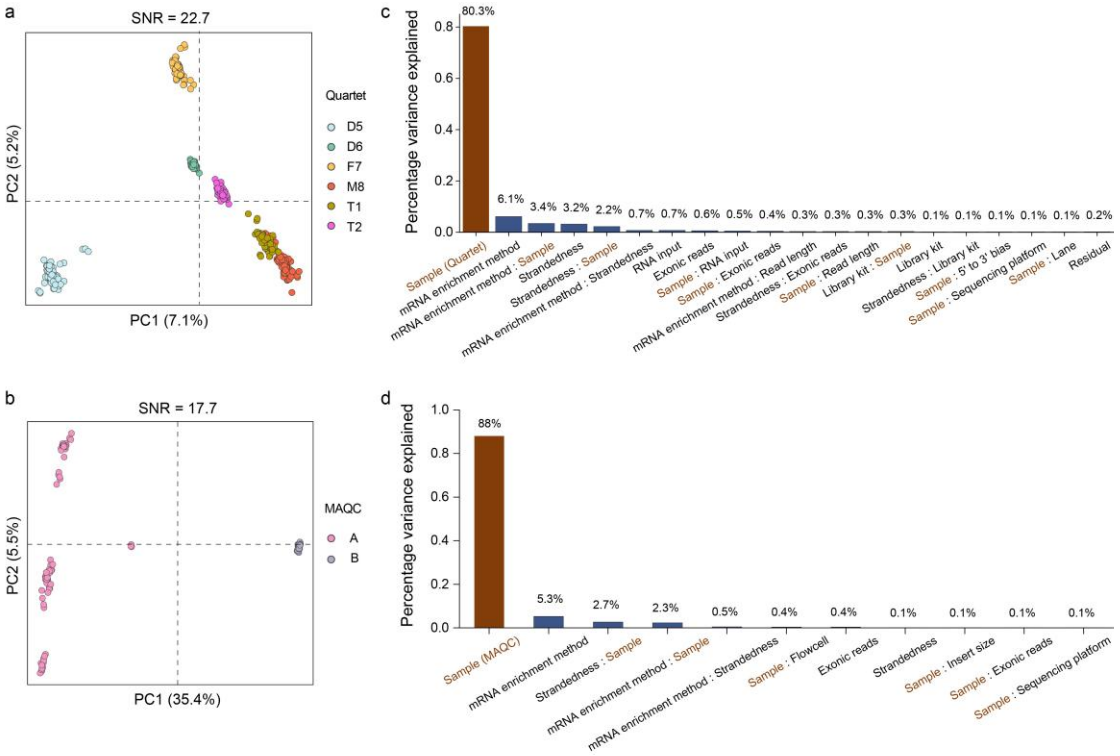

Supplementary Figure 32. Calculation of relative expression could eliminate the variations from experimental processes. Scatterplots of PCA on RNA-seq data of 26 laboratories that passed QC metrics for **(a)** the Quartet samples and **(b)** the MAQC samples at relative expression levels. Principal variance component analysis quantifies the proportion of variance explained by each experimental factor for **(c)** the Quartet samples and **(d)** the MAQC samples in relative expression levels. The red bars represent biological differences between samples. When calculating relative expression, the relative contribution of each experimental factor to total variations decreased compared to the biological difference across the Quartet (up) or MAQC (down) samples. SNR, signal-to-noise ratio. "Residual" represents unexplained variations.

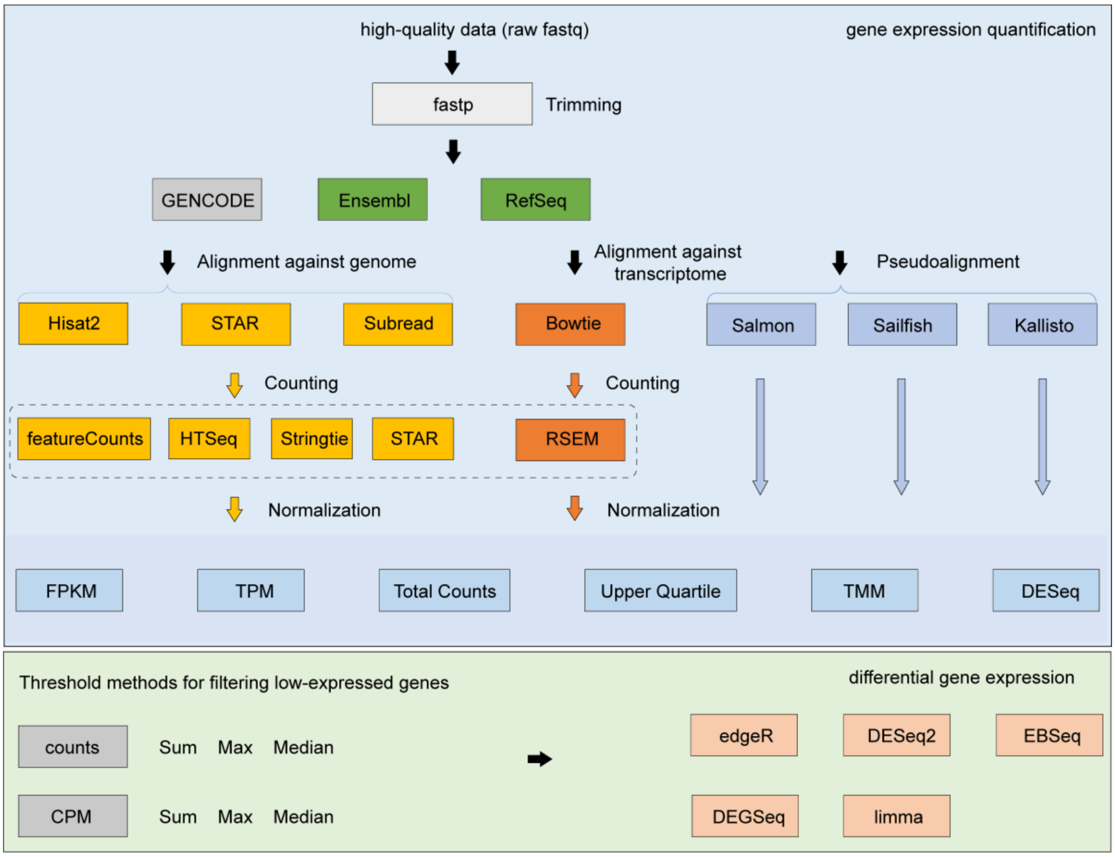

Supplementary Figure 33. The benchmark analysis workflow. Upper panel represents the raw gene expression quantification workflow. Every box contains the algorithms or methods used for the RNA-seq analysis at trimming, alignment, counting, and normalization. The lower panel represents the threshold methods evaluated for filtering low-expressed genes and the algorithms used for the differential gene analysis.

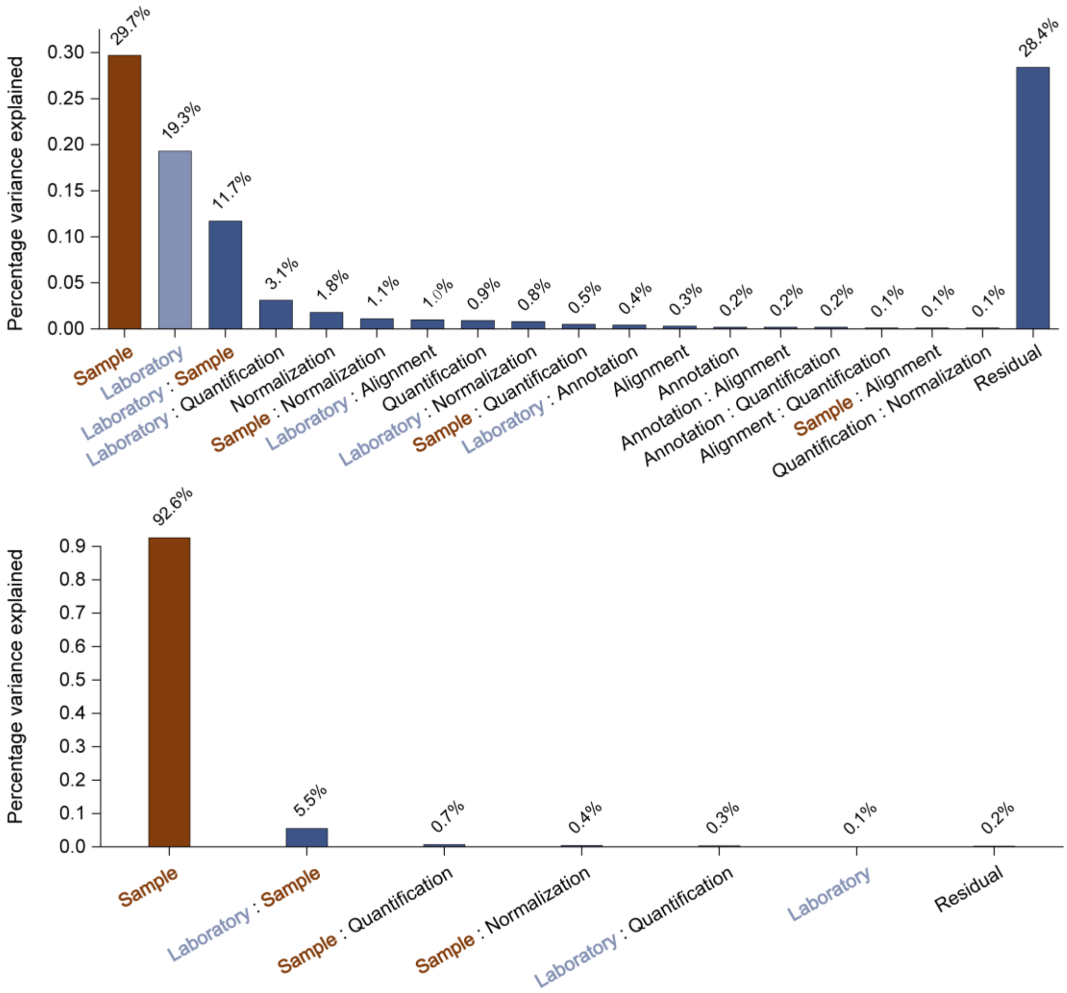

Supplementary Figure 34. Calculating relative expression could correct the influences of different bioinformatics tools. When calculating relative expression, the relative contribution of each bioinformatics step to total variations decreased compared to the biological difference across the Quartet (up) or MAQC (down) samples. For Quartet samples, there still 28.4% of variations from bioinformatics process that cannot be eliminated, implying the inherent performance difference in various analysis tools. The red bars represent biological differences between samples, while the light blue bars represent differences among benchmark datasets from 13 different laboratories.

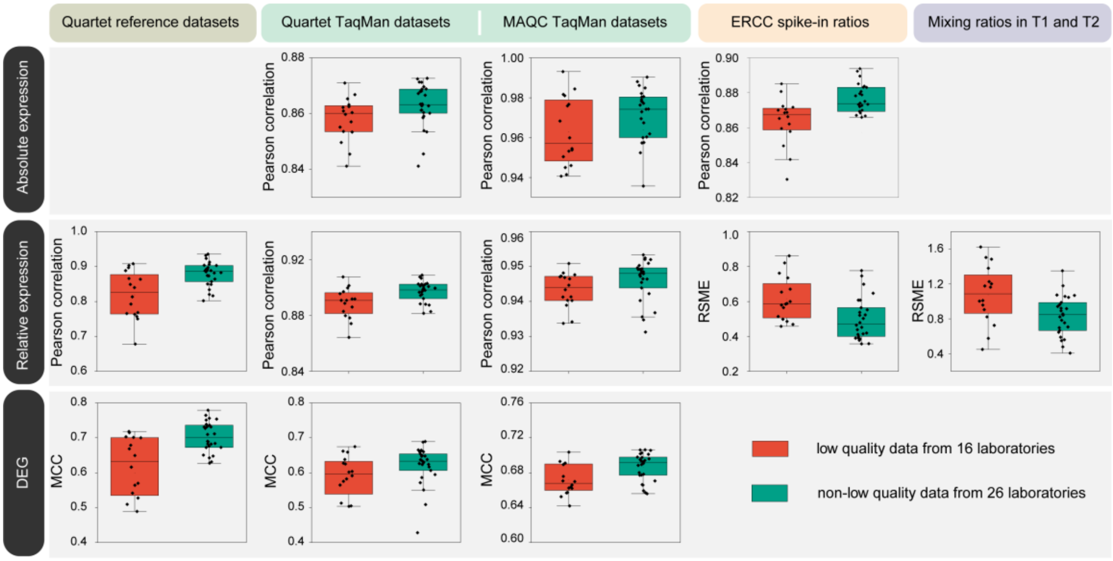

Supplementary Figure 35. Low accuracy for low-quality RNA-seq data. Based on four types of ground truth, RNA-seq data from 16 laboratories flagged as low quality exhibited low accuracy for absolute, relative gene expression, and DEG measurements, compared to the remaining RNA-seq data from 26 laboratories. Box plots present accuracy metrics for 16 low-quality laboratories and 26 non-low-quality laboratories, and data are presented as median values (center lines) and the upper and lower quartiles (box limits).

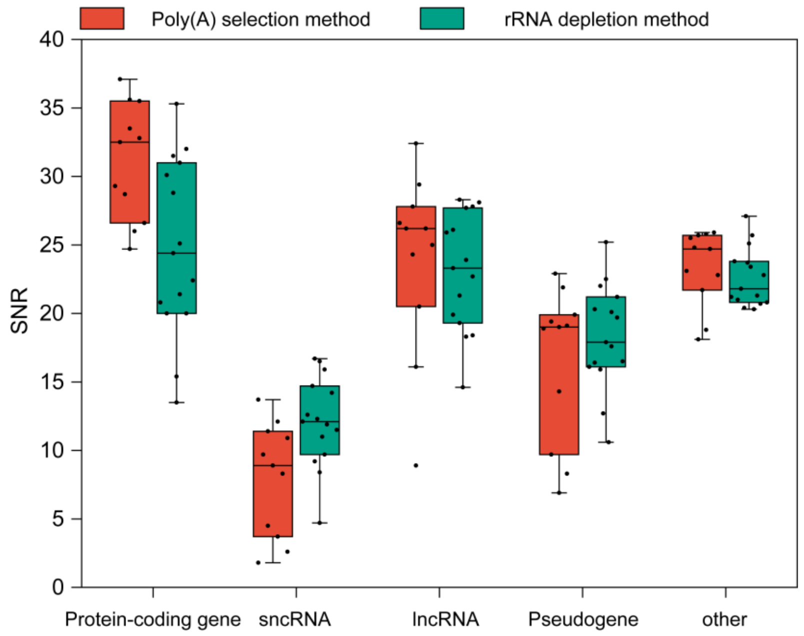

Supplementary Figure 36. Comparisons of SNR between two mRNA enrichment methods for different gene types. Using RNA-seq data from 26 laboratories passing quality metrics, the SNR values were calculated for five gene types, including protein-coding genes, small non-coding RNA (sncRNA), large non-coding RNA (sncRNA), pseudogenes, and others (Immunoglobulin/T-cell receptor gene segments). The box plots present SNR values for the two mRNA enrichment methods (Poly(A) selection (n = 11) and rRNA depletion (n = 15)) was compared, and data are presented as median values (center lines) and the upper and lower quartiles (box limits). The significance of the difference was tested using unpaired t-test. ** indicates a two-sided *p-value* < 0.01. ns, not significant.

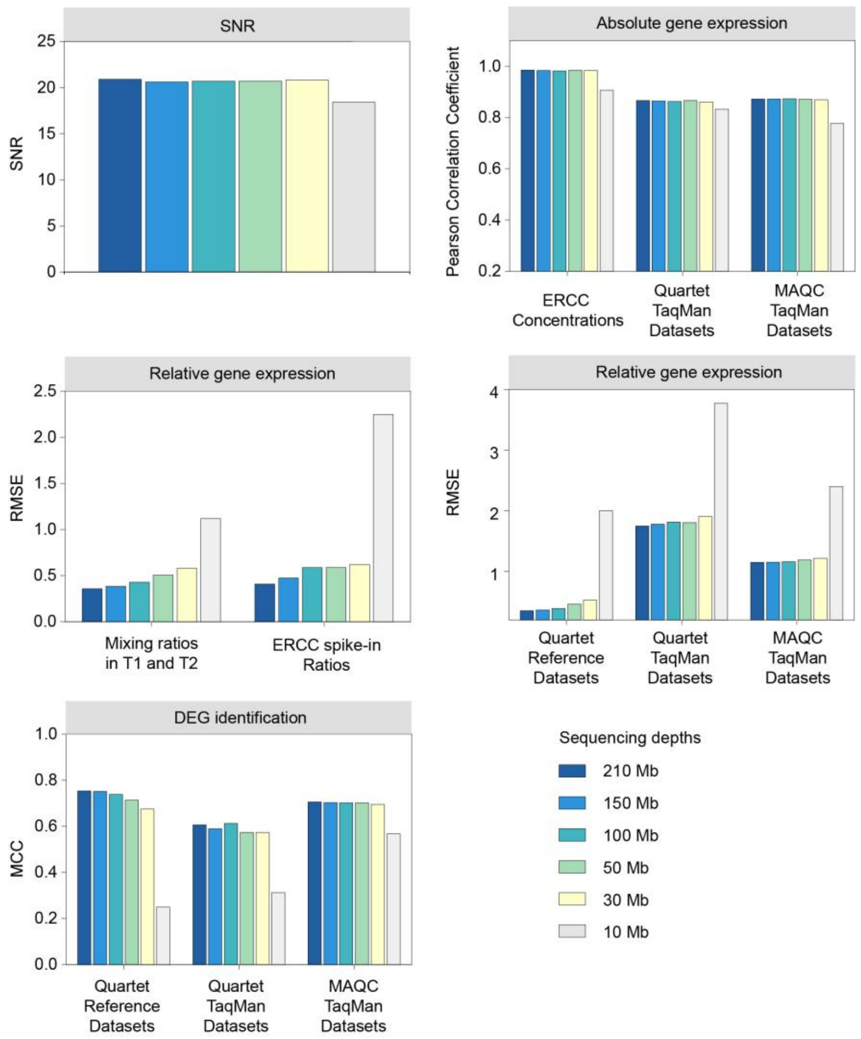

Supplementary Figure 37. The influence of read depths on RNA-seq performance. RNA-seq from lab28 was down-sampled into different depths (the number of fragments), including 150 Mb, 100 Mb, 100 Mb, 50 Mb, 30 Mb, and 10 Mb using seqtk (v.1.4)[9]. Then, genes were quantified using Ensembl annotation, STAR for alignment, and StringTie for gene quantification. Based on the four types of ground truths, the RNA-seq performance was assessed in multiple aspects: SNR, absolute and relative gene expression, and differentially expressed genes. MCC, Matthews Correlation Coefficient. RMSE, Root Mean Square Error.

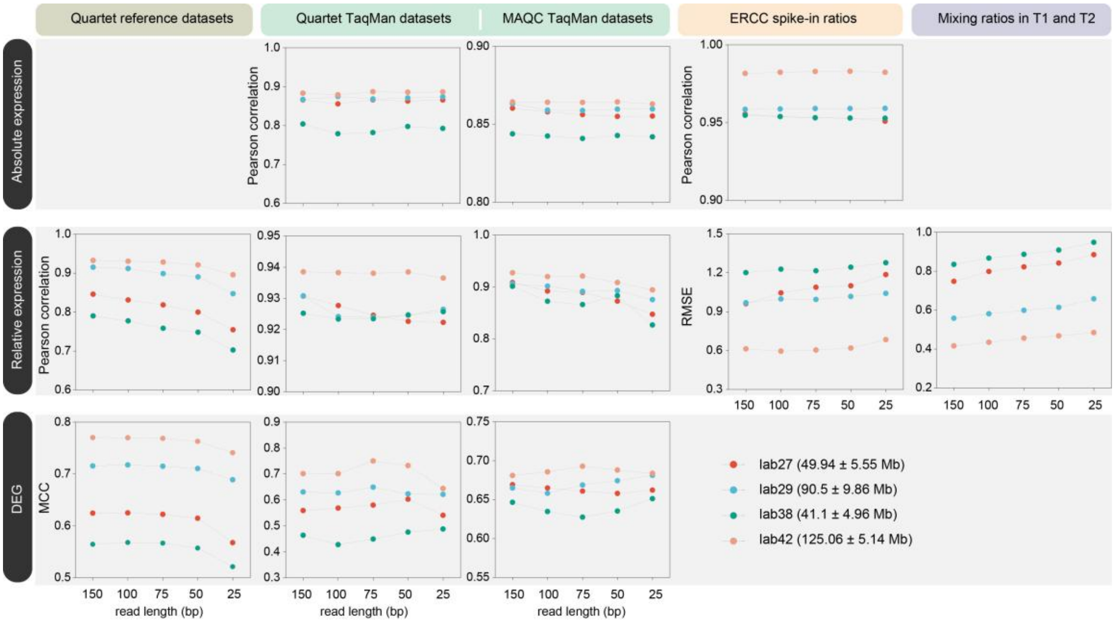

Supplementary Figure 38. The impact of read length on the RNA-seq performance. RNA-seq data with read length of 150 bp from four laboratories were shortened into 100 bp, 75 bp, 50 bp, and 25 bp using Cutadapt (v.4.8)[10]. Based on four types of ground truth, the accuracy of absolute, relative gene expression, and identification of differentially expressed genes (DEGs) for different read lengths were examined.

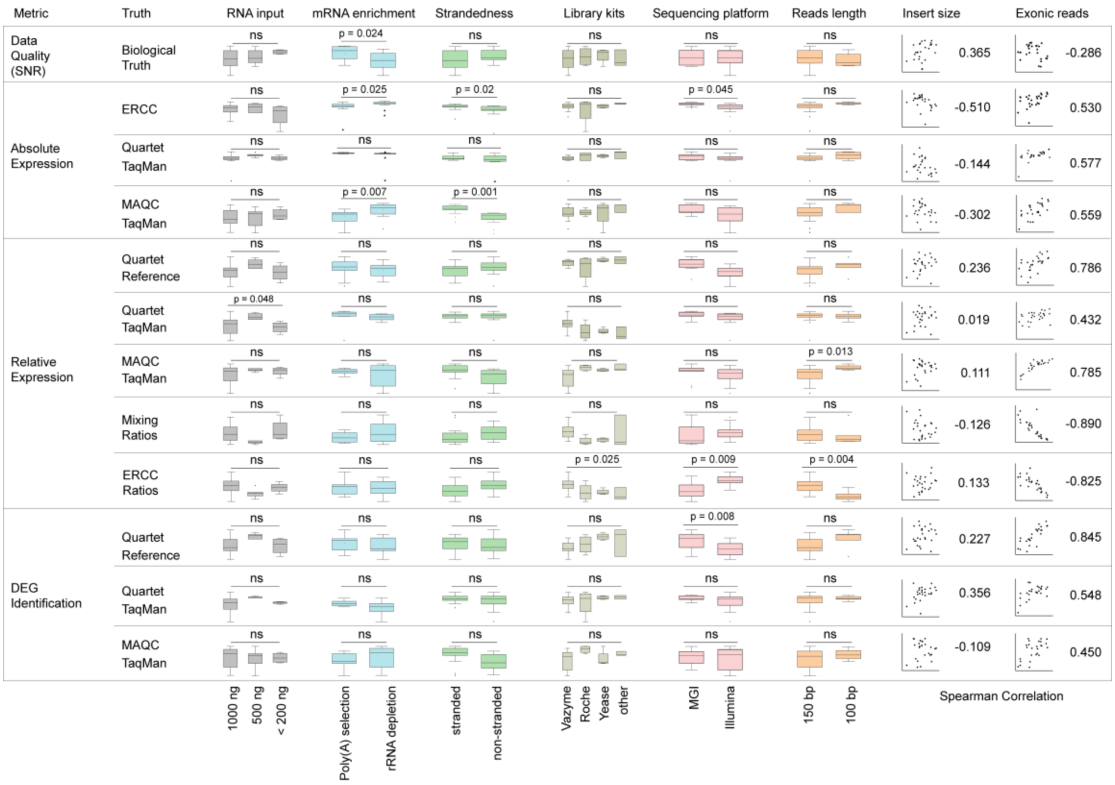

Supplementary Figure 39. The influence of experimental factors under different performance metrics. Performance metrics are divided into data quality, accuracy of absolute and relative expression and differential gene expression. The impact of the exonic reads and insert size is evaluated by Spearman correlation analyzes. Significance testing was conducted based on normal distribution assumptions using one-way analysis of variance (ANOVA) and paired t-tests, or, in cases where normal distribution was not observed, independent samples were subjected to Kruskal-Wallis test and Mann-Whitney U test. A two-sided *p-value* < 0.05 demonstrated statistically significant difference. ns, not significant.

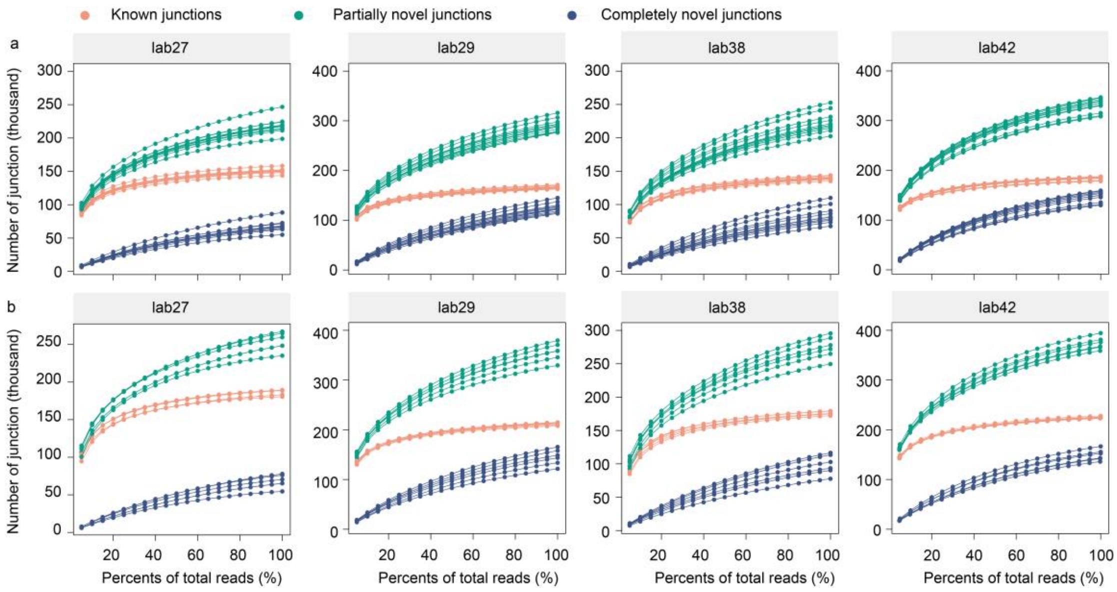

Supplementary Figure 40. The number of junctions detected with different sequencing depth. We resampled 5% to 95% of total alignments from BAM files of **(a)** Quartet and **(b)** MAQC samples from four laboratories, and then detected splice junctions from each subset and compares them to reference gene model using RSeQC (v.5.0.1)[11]. Even at low reads depths, such as in lab27 and lab38 with average mapped reads of 49.8 Mb and 41.1 Mb, respectively, known junctions could still be reliably detected. At moderate or high reads depths, such as lab29 and lab42 with average mapped reads of 125.6 Mb and 90.5 Mb, respectively, detection of unknown junctions still did not reach saturation.

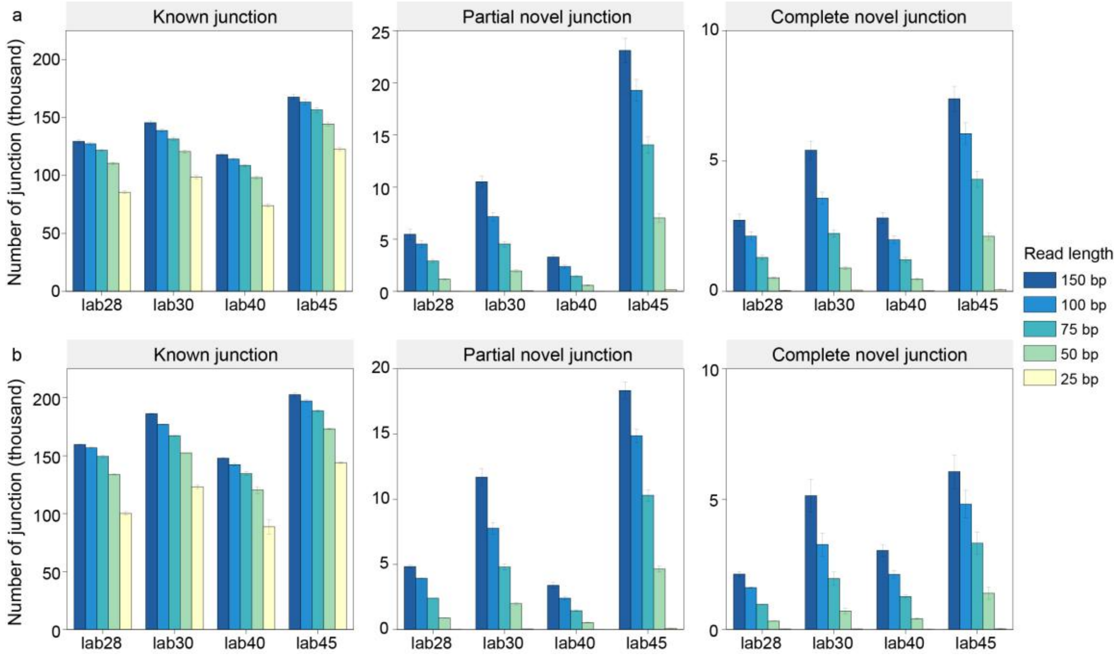

Supplementary Figure 41. The number of junctions detected with different read lengths. RNA-seq data with read length of 150 bp from four laboratories were shortened into 100 bp, 75 bp, 50 bp, and 25 bp using Cutadapt (v.4.8)[10]. The junctions were extracted from BAM files using RSeQC (v.5.0.1)[11], and could been classified into three types: known junctions, partially novel junctions, and completely novel junctions. In both **(a)** Quartet and **(b)** MAQC samples, longer read length led to the detection of more junctions. Error bars represent standard deviation of all Quartet or MAQC samples.

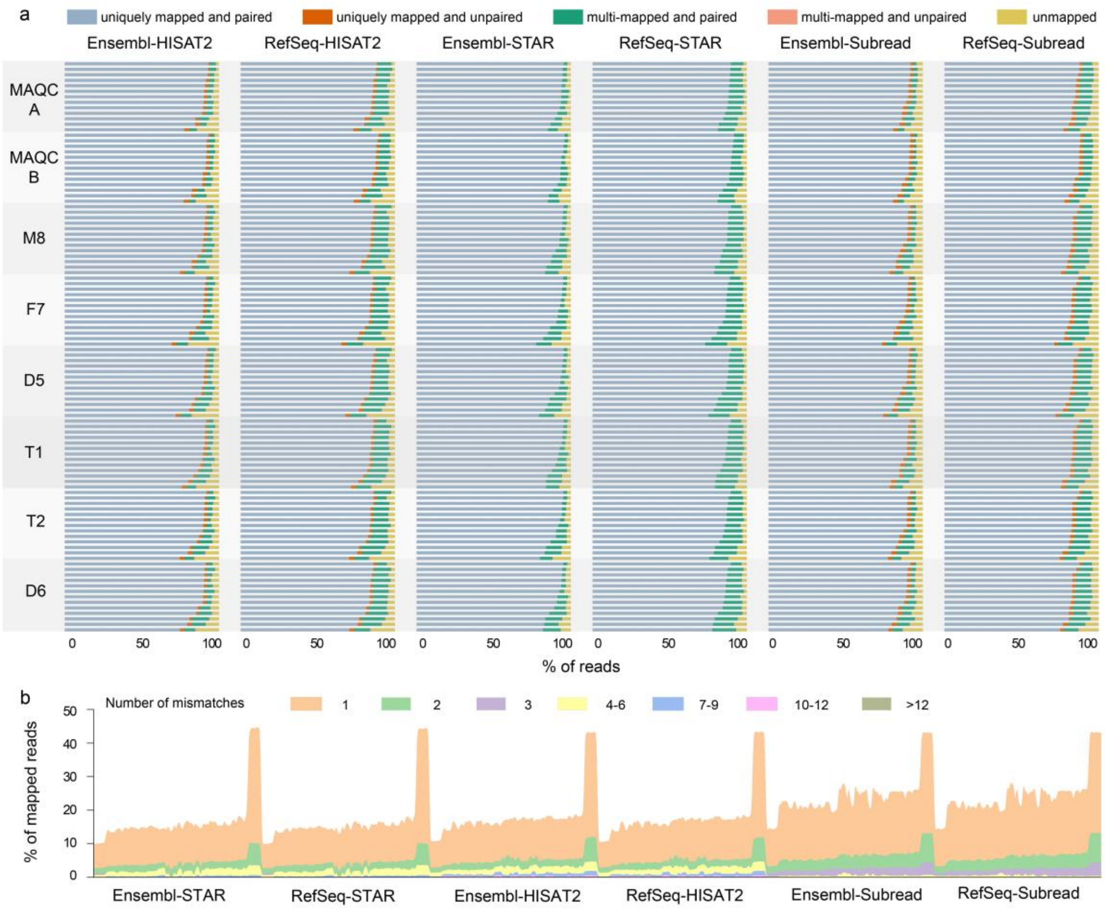

Supplementary Figure 42. Performance of different alignment schemes. (a) Distribution of mapping status of sequenced reads for six combinations of annotation and alignment tools. The 13 benchmark datasets corresponding to each sample are arranged in descending order based on the uniquely mapping rate. (b) Distribution of the number of reads with mismatch bases.

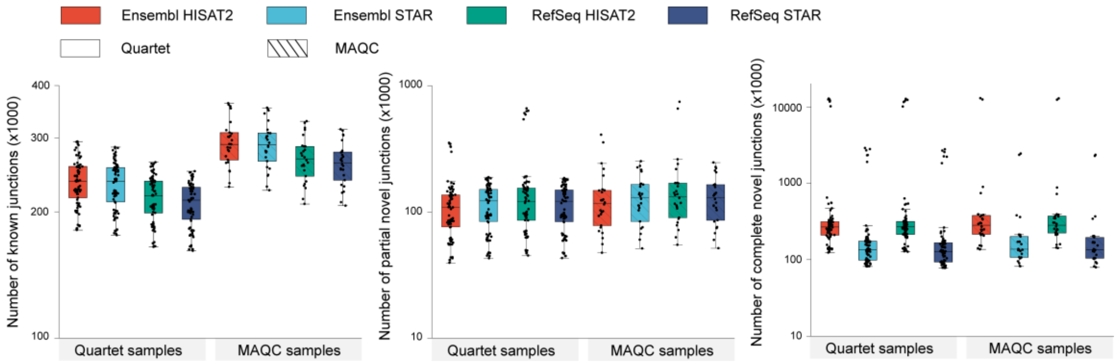

Supplementary Figure 43. Comparison of junctions detected by four alignment schemes. All the junctions supported by at least one reads in one of three replicates were included. Box plots present the number of junctions detected from 13 benchmark datasets for the Quartet (n = 78) and MAQC (n = 26) samples, and data are presented as median values (center lines) and the upper and lower quartiles (box limits).

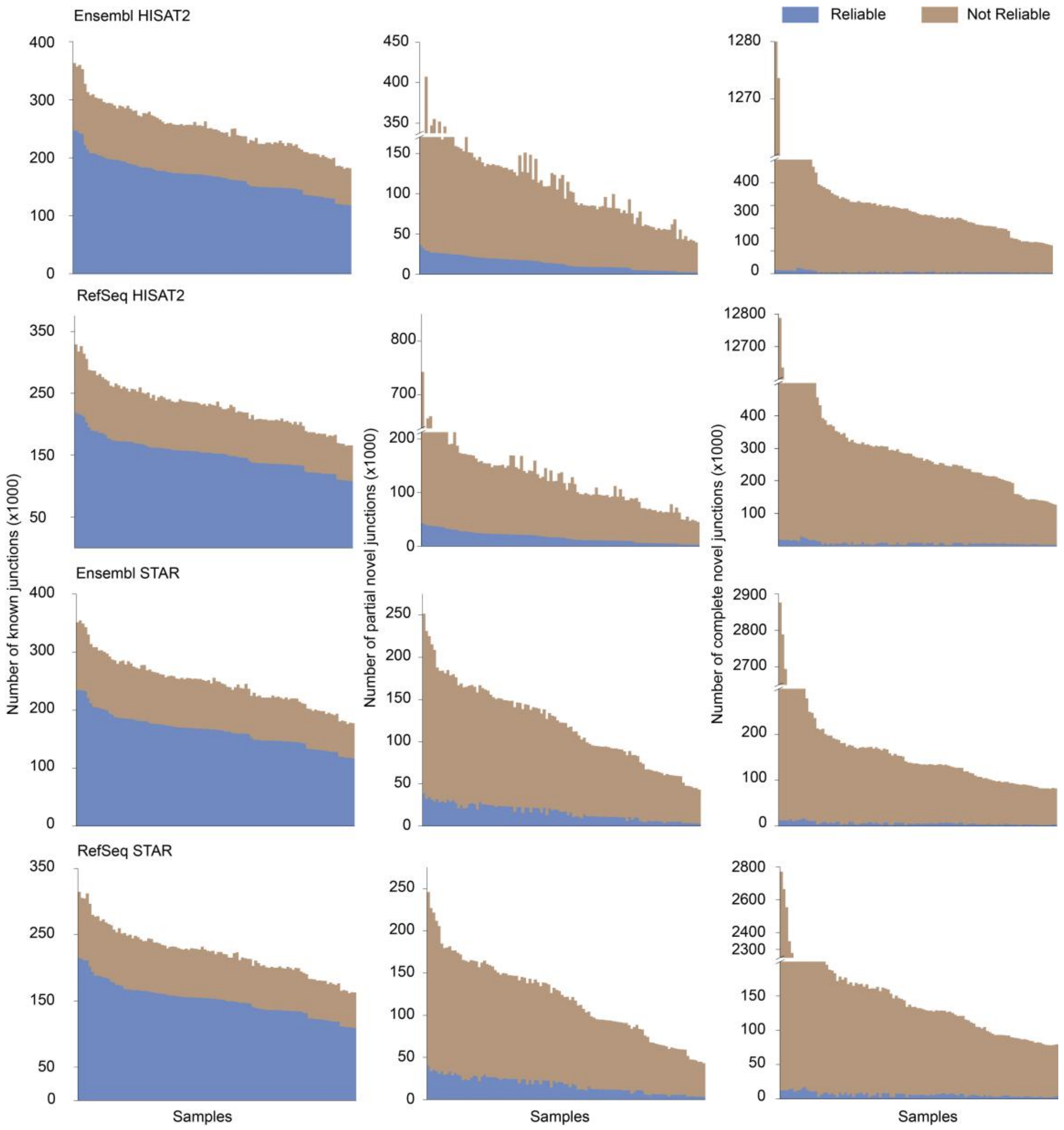

Supplementary Figure 44. The proportion of reliable and unreliable junctions detected by the four alignment schemes. Reliable junctions were defined as those supporting by at least one reads for all three replicates, while unreliable junctions were defined as those lacking read support in at least one of the three replicates. Most of the known junctions are reliable, while most of the novel junctions are unreliable. The x-axis represents the 13 benchmark datasets for eight sample groups (n=104).

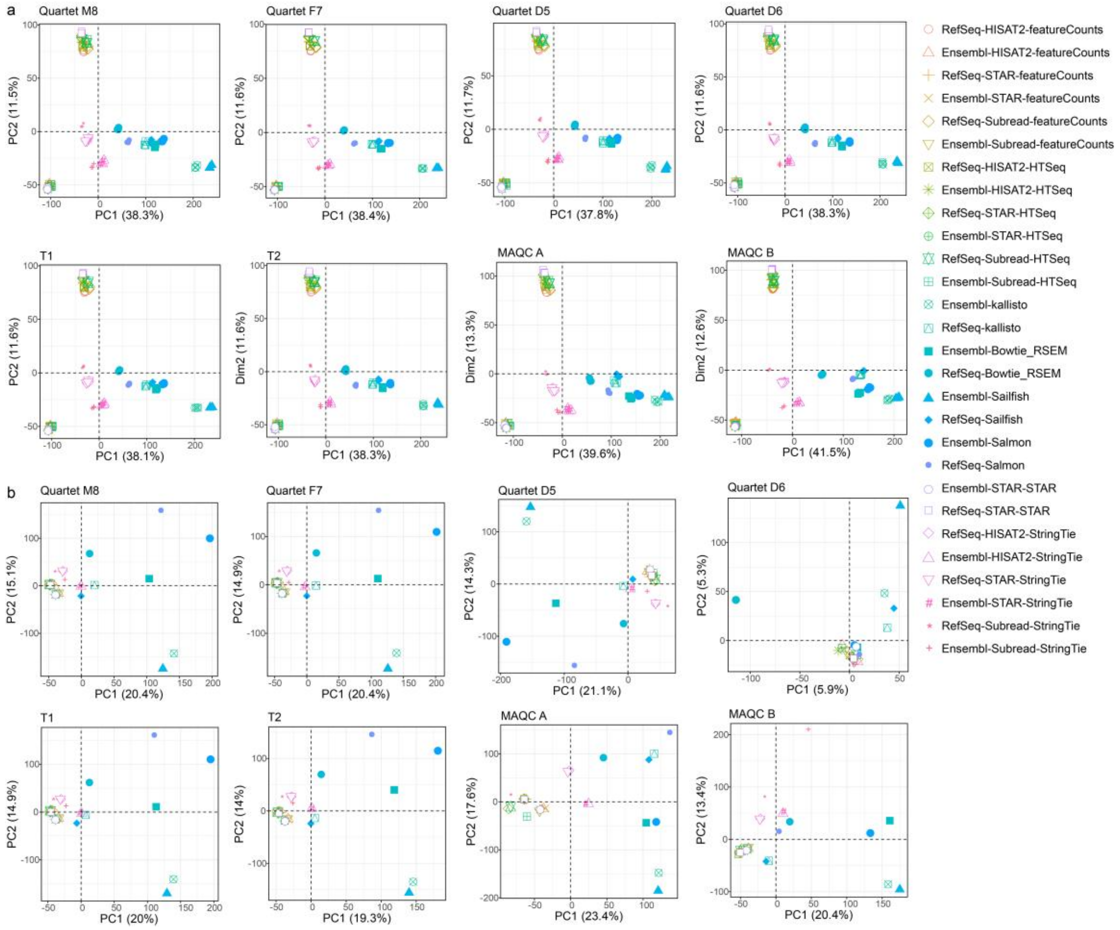

Supplementary Figure 45. Scatterplots of PCA on RNA-seq data from the 28 quantification pipelines. Clustering patterns of all quantification pipelines at **(a)** absolute and **(b)** relative expression levels. The RNA-seq data from lab01 were included for PCA analyzes, and identical shapes and colors represent replicates in the PCA plots.

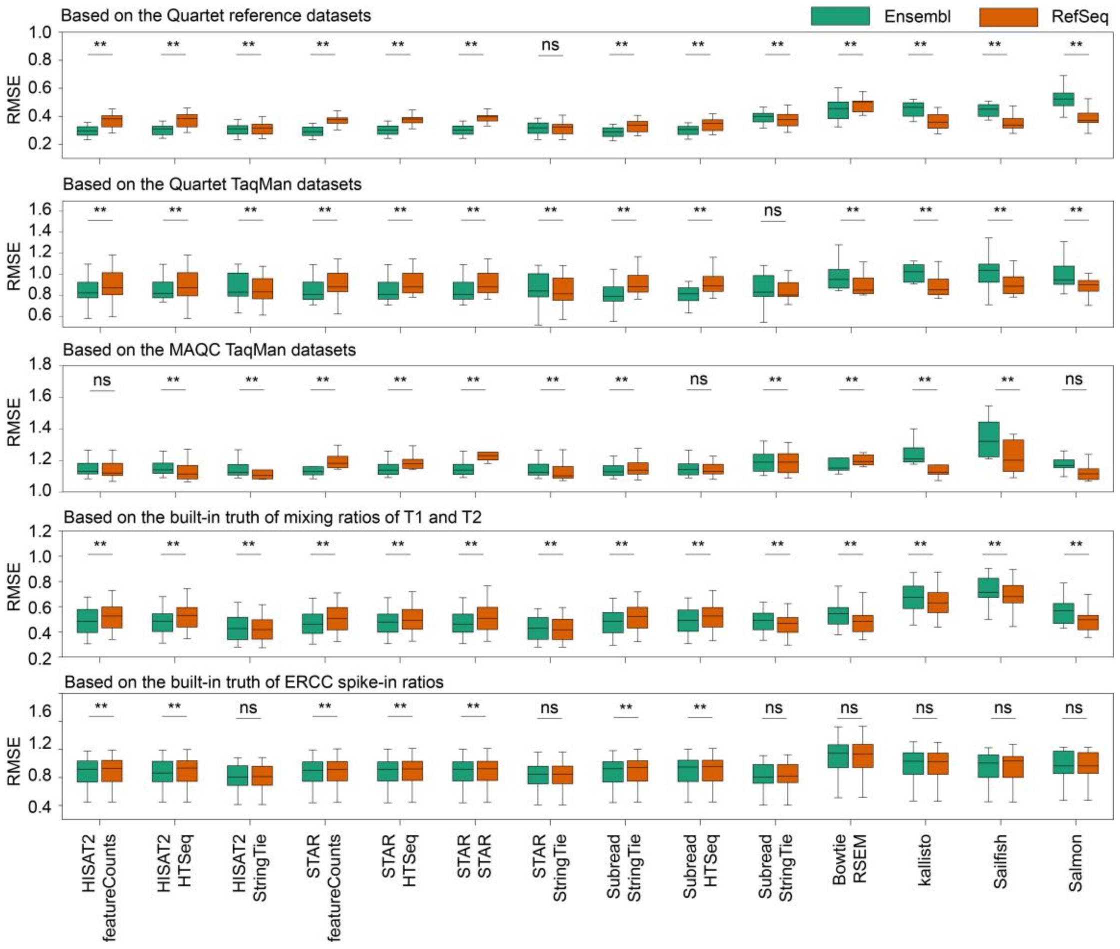

Supplementary Figure 46. The impact of gene annotation on accuracy of relative expression. All 28 quantification pipelines are evaluated based on the four types of ground truths, including the Quartet reference datasets, TaqMan datasets for Quartet and MAQC samples, built-in truth of mixing ratios in T1 and T2, and built-in truth of ERCC spike-in ratios. The impact of gene annotations was analyzed under the same conditions of alignment and quantification tools. Box plots present RMSE values for 13 benchmark datasets, and data are presented as median values (center lines) and the upper and lower quartiles (box limits). Significance testing was conducted using paired t-tests. * represents two-sided *p-value* < 0.05. ns, not significant. RMSE, Root Mean Square Error.

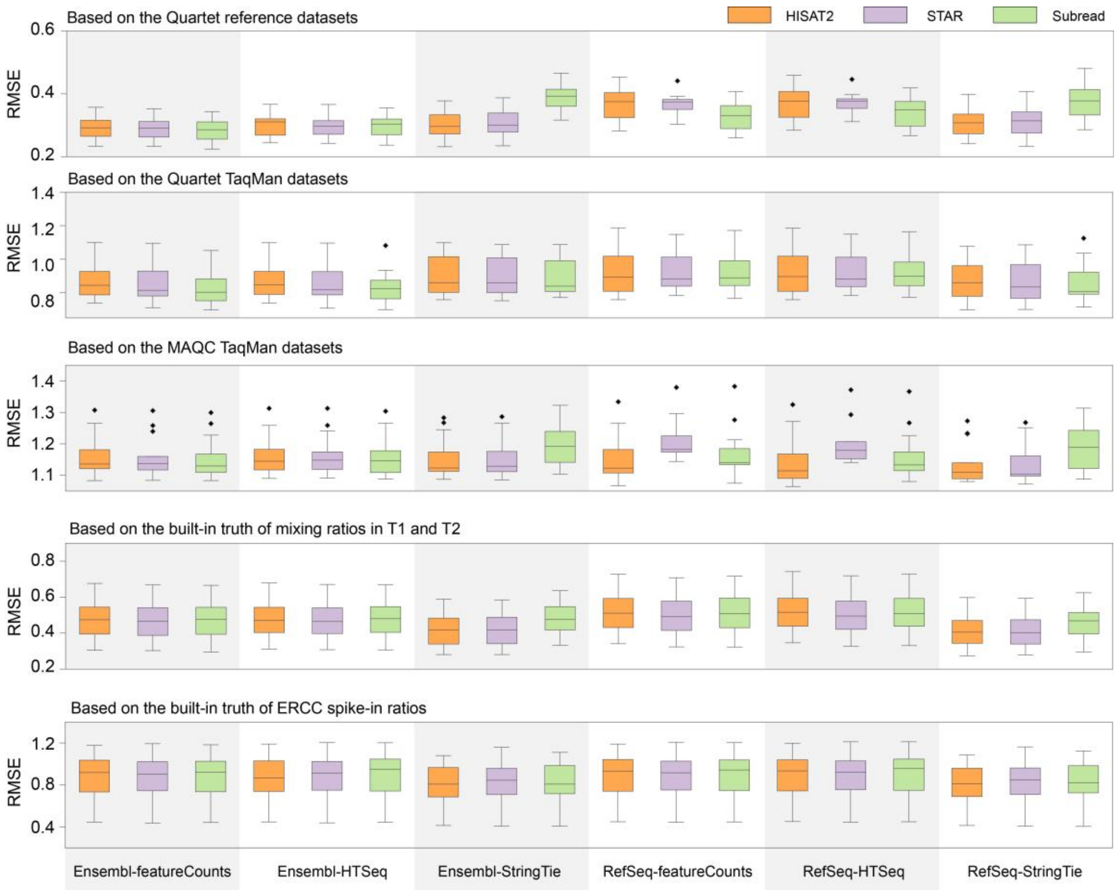

Supplementary Figure 47. The impact of alignment tools on the accuracy of relative expression. All 28 quantification pipelines involving sequence alignment are evaluated based on the four types of ground truths, including the Quartet reference datasets, the TaqMan datasets for Quartet and MAQC samples, built-in truth of mixing ratios in T1 and T2, and built-in truth of ERCC spike-in ratios. The impact of alignment tools was analyzed under the same conditions of gene annotation and quantification tools. Box plots present RMSE values for 13 benchmark datasets, and data are presented as median values (center lines) and the upper and lower quartiles (box limits). RMSE, Root Mean Square Error.

Ground truth	Overall Rank	Rank	mean ± sd	Rank	mean ± sd	Rank	mean ± sd	Rank	mean ± sd	Rank	mean ± sd
		Quartet Reference datasets		Quartet TaqMan datasets		MAQC TaqMan datasets		Mixing ratios in T1 and T2		ERCC spike-in Ratios	
RefSeq_HISAT2_StringTie	1	9	0.314 ± 0.041	10	0.883 ± 0.106	2	1.137 ± 0.066	2	0.421 ± 0.084	2	0.825 ± 0.184
RefSeq_STAR_StringTie	2	10	0.315 ± 0.044	8	0.881 ± 0.113	3	1.139 ± 0.065	1	0.42 ± 0.085	4	0.84 ± 0.193
Ensembl_Subread_featureCounts	3	1	0.289 ± 0.032	1	0.834 ± 0.087	8	1.159 ± 0.067	10	0.488 ± 0.097	9	0.901 ± 0.201
Ensembl_STAR_featureCounts	4	2	0.294 ± 0.032	5	0.873 ± 0.101	9	1.163 ± 0.066	6	0.484 ± 0.099	8	0.898 ± 0.203
Ensembl_HISAT2_StringTie	5	8	0.308 ± 0.041	14	0.905 ± 0.111	5	1.157 ± 0.066	3	0.428 ± 0.087	1	0.823 ± 0.183
Ensembl_STAR_StringTie	6	11	0.316 ± 0.042	13	0.905 ± 0.109	6	1.159 ± 0.066	4	0.43 ± 0.085	3	0.839 ± 0.192
Ensembl_STAR_HTSeq	7	5	0.302 ± 0.033	3	0.873 ± 0.1	13	1.169 ± 0.066	7	0.485 ± 0.098	11	0.902 ± 0.204
Ensembl_HISAT2_featureCounts	8	3	0.296 ± 0.033	7	0.877 ± 0.1	12	1.167 ± 0.068	12	0.49 ± 0.101	10	0.902 ± 0.2
Ensembl_STAR_STAR	9	6	0.302 ± 0.033	4	0.873 ± 0.1	14	1.169 ± 0.066	8	0.485 ± 0.098	12	0.902 ± 0.204
Ensembl_HISAT2_HTSeq	10	7	0.304 ± 0.034	6	0.877 ± 0.099	15	1.17 ± 0.067	13	0.491 ± 0.1	7	0.897 ± 0.202
Ensembl_Subread_HTSeq	11	4	0.3 ± 0.033	2	0.849 ± 0.094	11	1.167 ± 0.066	14	0.494 ± 0.096	18	0.912 ± 0.205
RefSeq_Subread_StringTie	12	20	0.38 ± 0.048	9	0.882 ± 0.112	19	1.19 ± 0.072	5	0.463 ± 0.081	6	0.849 ± 0.192
Ensembl_Subread_StringTie	13	23	0.393 ± 0.039	12	0.892 ± 0.099	21	1.207 ± 0.068	9	0.488 ± 0.081	5	0.844 ± 0.191
RefSeq_Salmon	14	22	0.388 ± 0.058	17	0.932 ± 0.103	1	1.123 ± 0.048	15	0.495 ± 0.09	22	0.976 ± 0.219
RefSeq_HISAT2_HTSeq	15	19	0.377 ± 0.044	19	0.935 ± 0.124	4	1.151 ± 0.083	22	0.534 ± 0.107	16	0.91 ± 0.204
RefSeq_HISAT2_featureCounts	16	18	0.373 ± 0.044	21	0.936 ± 0.122	7	1.159 ± 0.079	21	0.531 ± 0.106	17	0.911 ± 0.202
RefSeq_Subread_featureCounts	17	12	0.333 ± 0.041	18	0.933 ± 0.097	16	1.173 ± 0.08	19	0.526 ± 0.103	19	0.914 ± 0.204
RefSeq_Subread_HTSeq	18	14	0.343 ± 0.041	20	0.936 ± 0.092	10	1.166 ± 0.079	20	0.529 ± 0.104	20	0.926 ± 0.207
RefSeq_STAR_featureCounts	19	16	0.365 ± 0.036	24	0.947 ± 0.107	23	1.214 ± 0.067	16	0.514 ± 0.104	13	0.907 ± 0.205
RefSeq_STAR_HTSeq	20	17	0.369 ± 0.035	22	0.946 ± 0.109	22	1.207 ± 0.069	18	0.516 ± 0.105	14	0.91 ± 0.206
RefSeq_kallisto	21	15	0.359 ± 0.048	11	0.892 ± 0.099	17	1.175 ± 0.094	25	0.657 ± 0.106	26	1.008 ± 0.217
RefSeq_STAR_STAR	22	21	0.385 ± 0.033	23	0.946 ± 0.109	26	1.255 ± 0.064	17	0.514 ± 0.105	15	0.91 ± 0.206
RefSeq_Sailfish	23	13	0.341 ± 0.043	15	0.916 ± 0.102	24	1.223 ± 0.1	27	0.708 ± 0.104	24	0.983 ± 0.225
RefSeq_Bowtie_RSEM	24	27	0.494 ± 0.064	16	0.92 ± 0.128	25	1.223 ± 0.068	11	0.489 ± 0.114	27	1.09 ± 0.276
Ensembl_Salmon	25	28	0.524 ± 0.073	26	1.025 ± 0.122	18	1.185 ± 0.071	24	0.575 ± 0.102	23	0.979 ± 0.22
Ensembl_Bowtie_RSEM	26	25	0.447 ± 0.078	25	1.003 ± 0.137	20	1.192 ± 0.079	23	0.55 ± 0.119	28	1.09 ± 0.275
Ensembl_Sailfish	27	24	0.445 ± 0.046	28	1.046 ± 0.125	28	1.342 ± 0.118	28	0.736 ± 0.103	21	0.976 ± 0.221
Ensembl_kallisto	28	26	0.459 ± 0.046	27	1.046 ± 0.112	27	1.269 ± 0.109	26	0.694 ± 0.106	25	0.999 ± 0.216

RMSE: high – low

Supplementary Figure 48. The performance ranking of 28 quantification pipelines at relative expression levels. The four types of ground truth, arranged from left to right, include the Quartet reference datasets, the TaqMan datasets for the Quartet and MAQC samples, the built-in truth regarding the recovery of mixed ratios in T1 and T2, and the built-in truth of ERCC spike-in ratios. The average Root Mean Square Error (RMSE) between relative gene expression and the ground truth was assessed across 13 benchmark datasets. Rankings were determined in ascending order based on RMSE, and the final ranking was calculated as the average of rankings derived from the five truths.

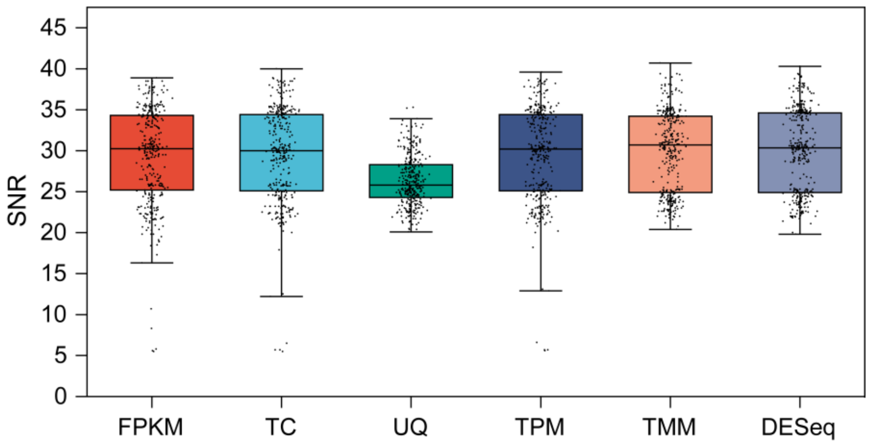

Supplementary Figure 49. Comparison of the SNR of RNA-seq data from different normalization methods. Read counts from 28 quantification pipelines were normalized using six normalization methods. Box plots present SNR values from 28 quantification pipelines for 13 benchmark datasets, and data are presented as median values (center lines) and the upper and lower quartiles (box limits).

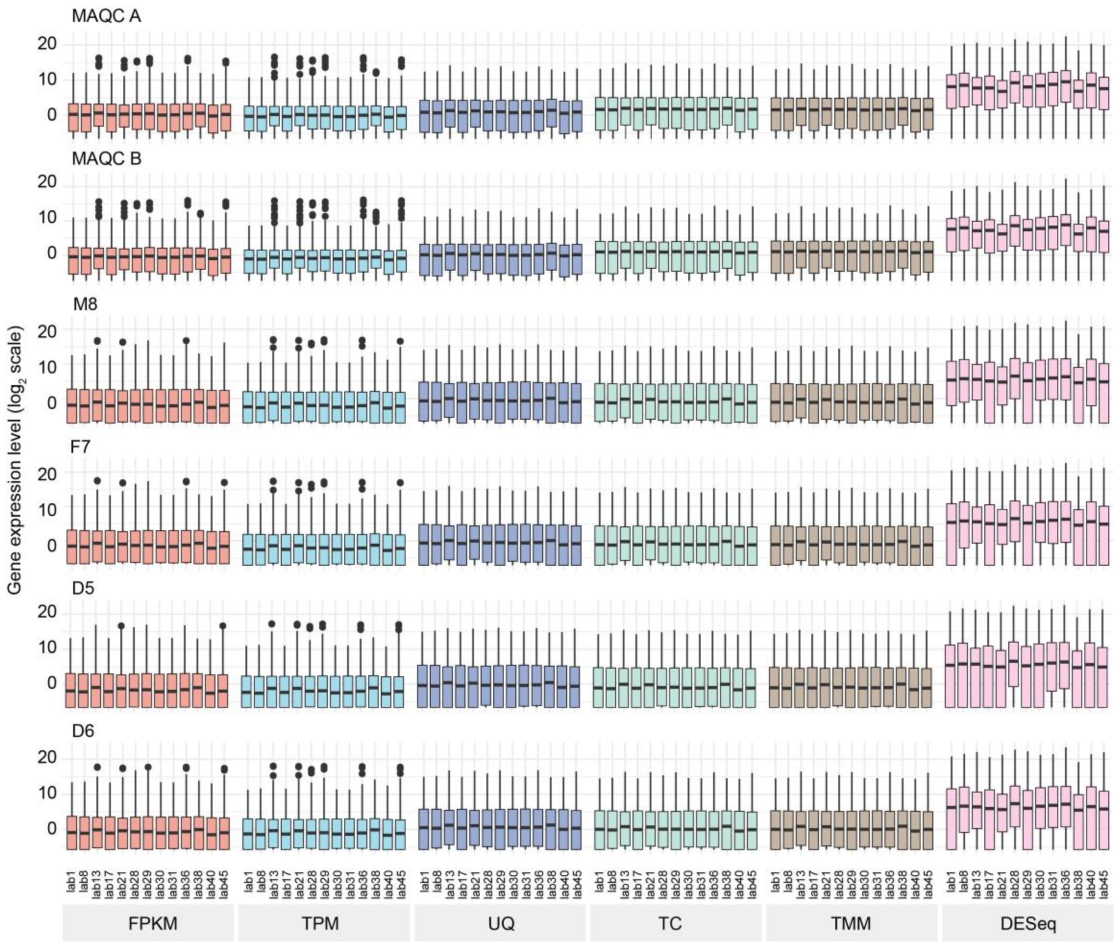

Supplementary Figure 50. Distribution of gene expression using different normalization methods. The gene expression was quantified using the Ensembl-STAR-StringTie pipeline. Box plots present gene expression values for 62,710 genes, and data are presented as median values (center lines) and the upper and lower quartiles (box limits).

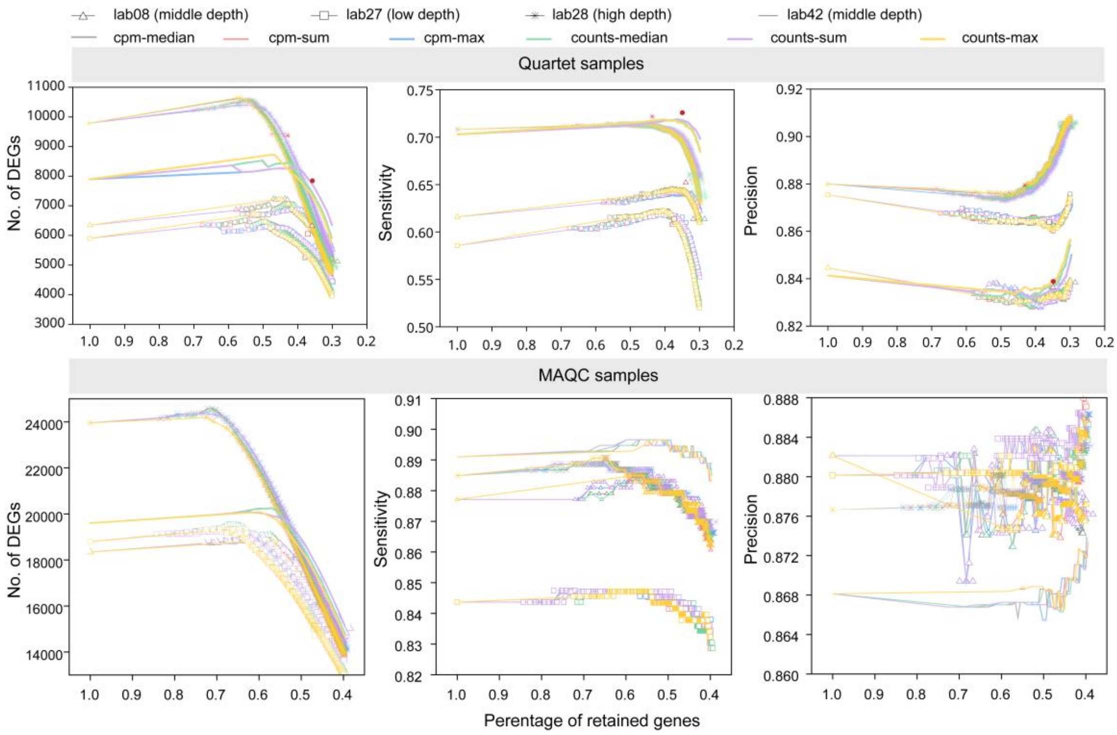

Supplementary Figure 51. Quantitative assessment of low-expression gene filtering methods. RNA-seq data representing high, medium, and low sequencing depth from four laboratories were employed for validation of different filtering conditions. Using the Ensembl-STAR-StringTie-edgeR pipeline, we calculated the number of DEGs, the true positive rate (sensitivity), and the precision as a function of filtering threshold for different filtering methods in Quartet samples (upper panel) and MAQC samples (lower panel). The red dots with different shapes represent the automated thresholds by edgeR.

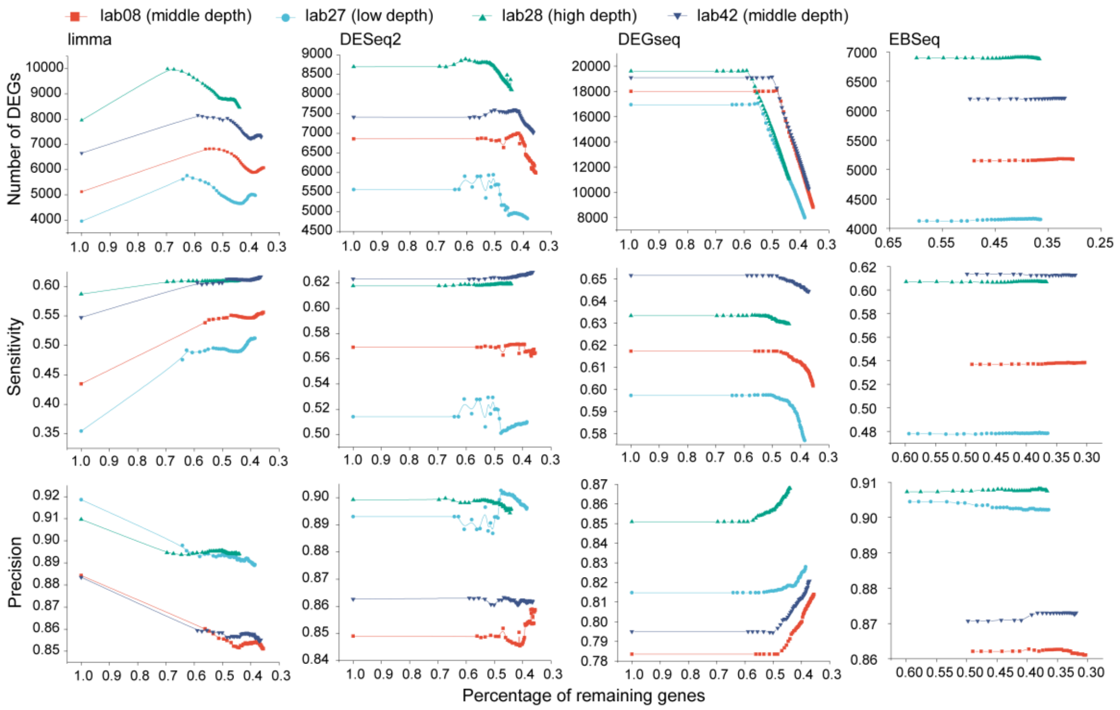

Supplementary Figure 52. Quantitative assessment of low-expression gene filtering for different differential analysis tools. For other tools including limma[12], DESeq2[13], DEGseq[14], and EBSeq[15], we filtered low-expression genes based on the sum of reads counts, and calculated the number of DEGs, the true positive rate (sensitivity), and the precision as a function of filtering threshold.

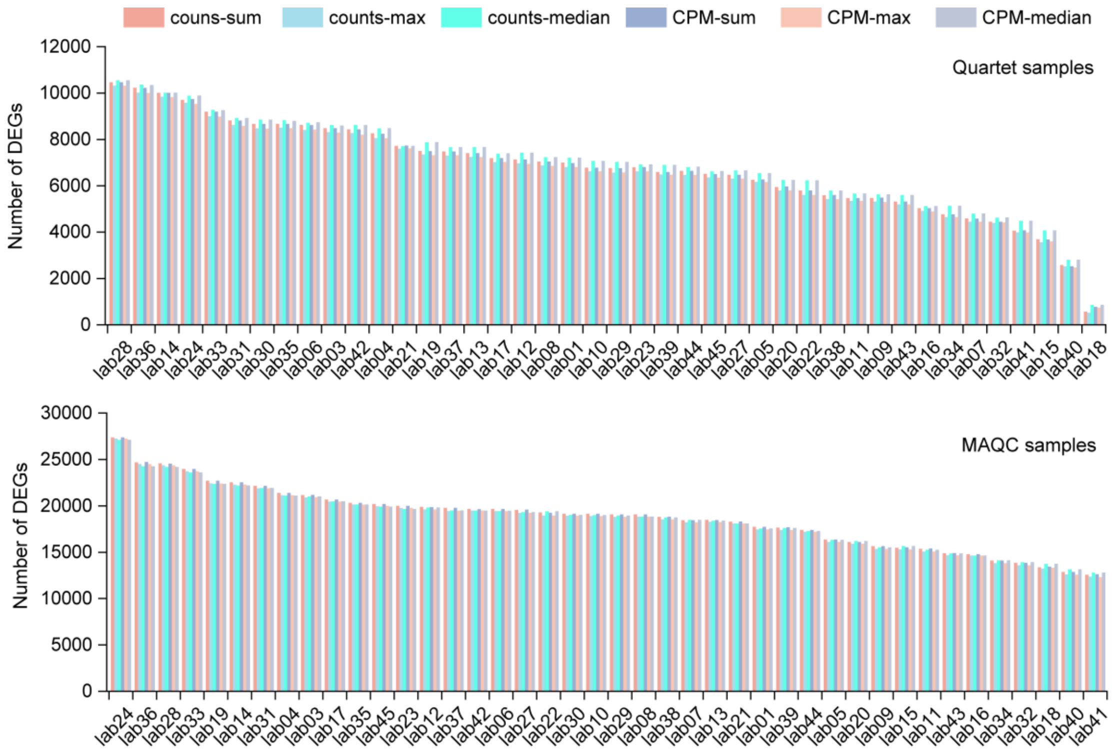

Supplementary Figure 53. Comparison of the maximal number of DEGs using different filtering methods. The maximal number of DEGs for Quartet (up) and MAQC (down) samples were calculated using edgeR after applying a series of filtering thresholds. DEGs, differentially expressed genes.

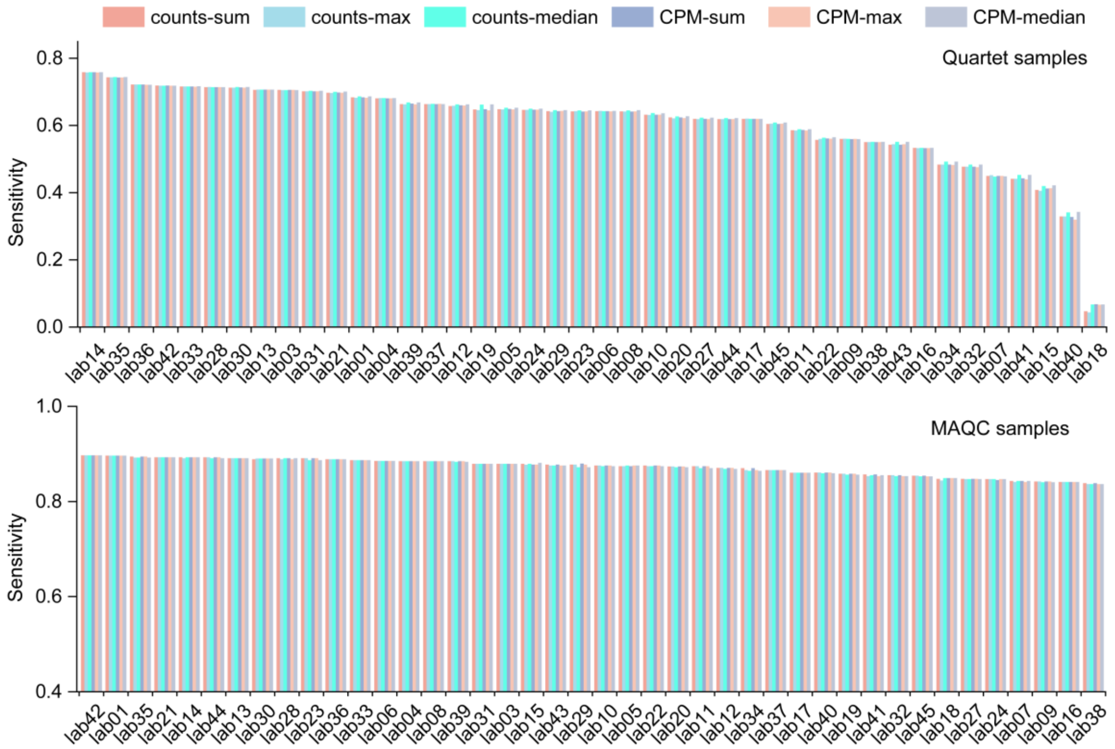

Supplementary Figure 54. Comparison of the maximal sensitivity using different filtering methods. The differential expression profiles for Quartet (up) and MAQC (down) samples at different threshold values were compared to the Quartet reference datasets and MAQC TaqMan datasets, respectively, to calculate the maximal true positive rate. The edgeR was used for differential expression analysis. CPM, counts per million.

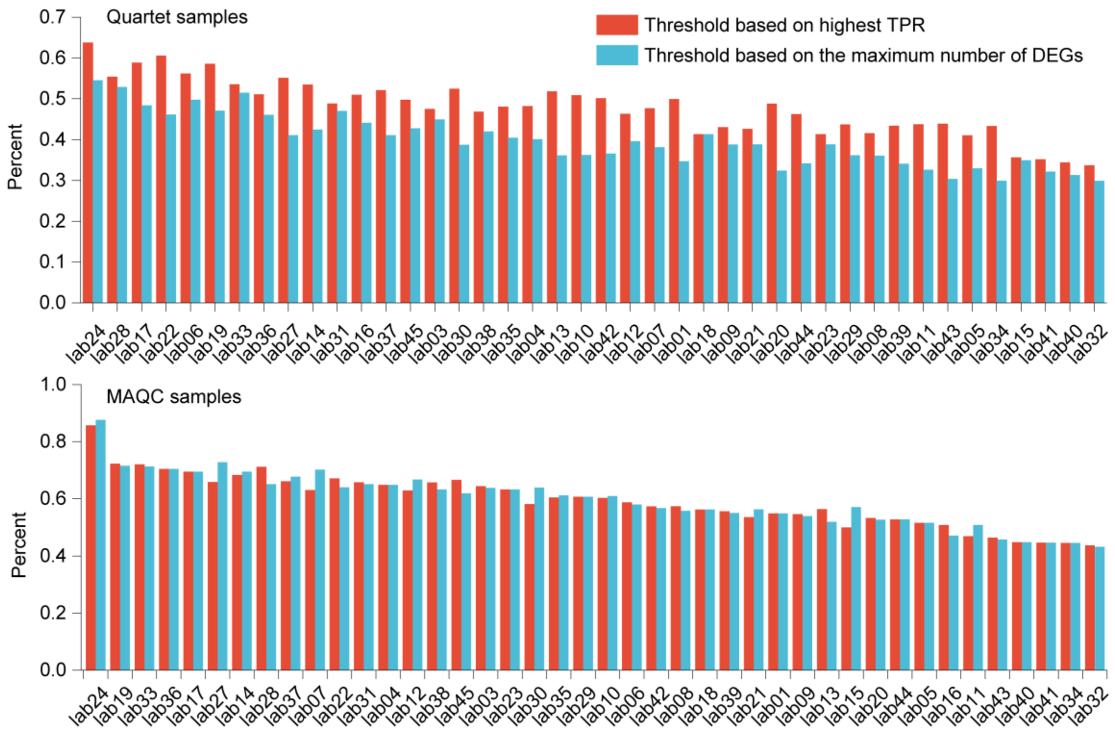

Supplementary Figure 55. Comparison of the optimal threshold values determined by maximum number of DEGs and highest TPR. For all RNA-seq from laboratories, the threshold values corresponding to the highest TPR and the the maximum number of DEGs were calculated and compared for Quartet (up) and MAQC (down) samples. TPR, true positive rate.

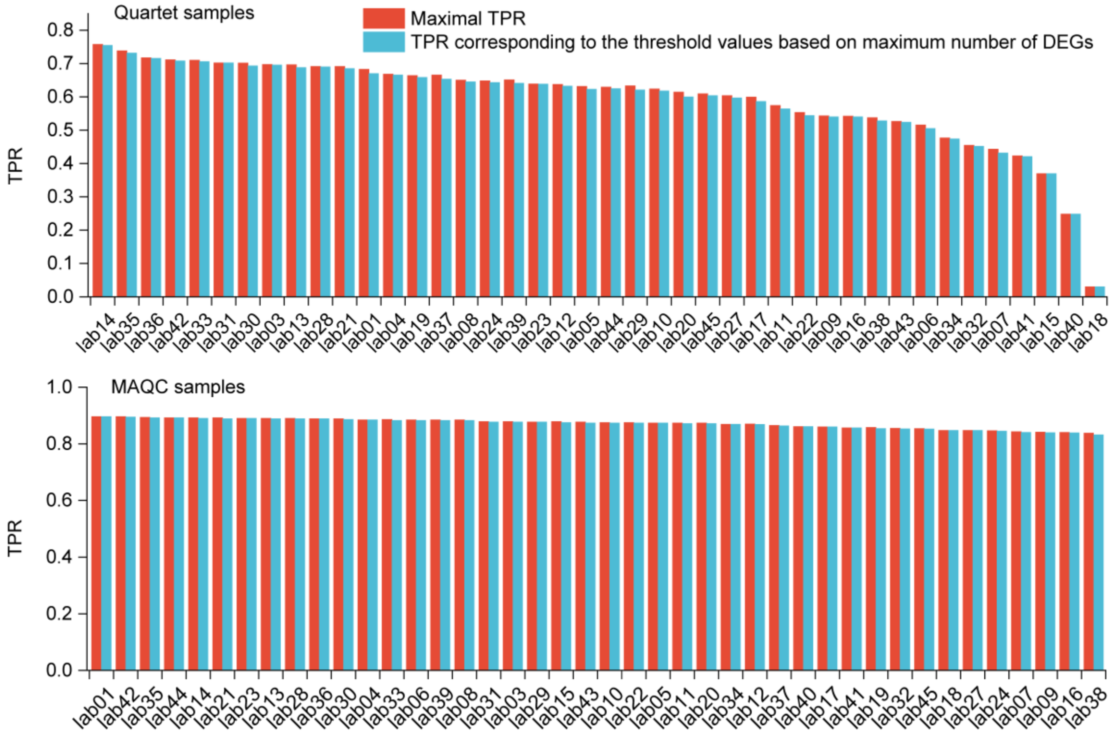

Supplementary Figure 56. Comparison of the TPR corresponding to thresholds determined by maximum total number of DEGs and highest TPR. The differential expression profiles for Quartet (up) and MAQC (down) samples at different thresholds were compared to the Quartet reference datasets and MAQC TaqMan datasets, respectively, to calculate the TPR. TPR, true positive rate.

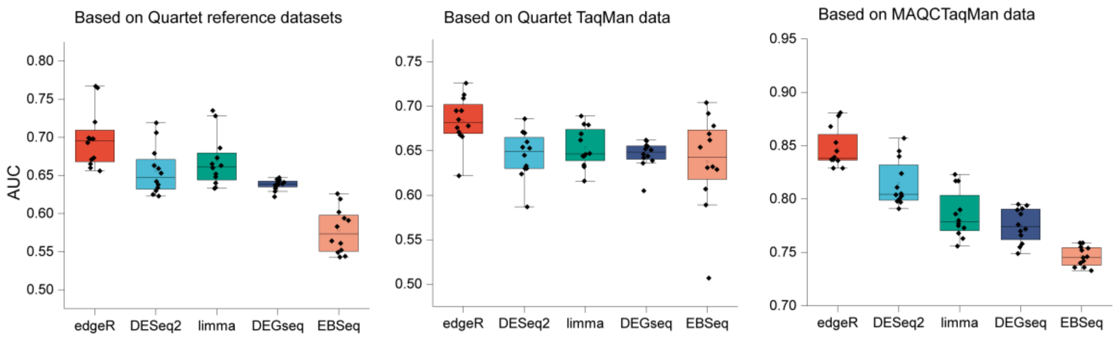

Supplementary Figure 57. The assessment of five differential analysis tools using AUC values. The box plots display the AUC values of each differential analysis tool for 13 benchmarked datasets, and data are presented as median values (center lines) and the upper and lower quartiles (box limits). AUC, The area under the receiver operating characteristic curve.

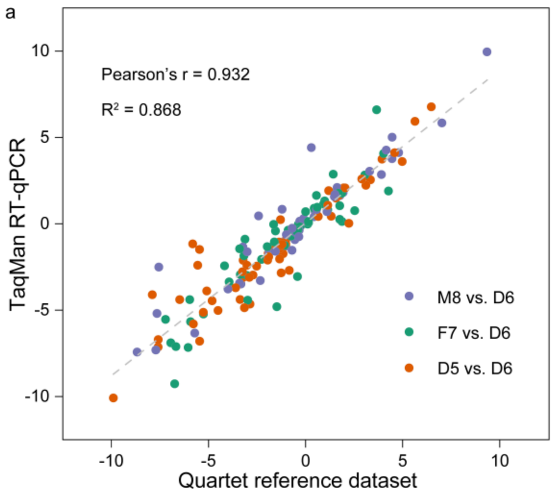

b

TaqMan RT-qPCR	Quartet reference datasets: NonDEG	Upregulate	Downregulate
NonDEG	37	8	10
Upregulate	2	41	0
Downregulate	2	0	84

Supplementary Figure 58. Validation of ratio-based Quartet reference dataset. (a) Scatter plots of log_2 fold changes (FC) of gene expression between Quartet reference datasets and TaqMan RT-qPCR. **(b)** Comparison of differentially expressed genes (DEGs) between Quartet reference datasets and TaqMan RT-qPCR. A total of 88% of genes with consistent classification results.

2. Supplementary Tables

Supplementary Table 1. Best practice recommendations.

Key considerations	Recommendations
1. RNA-seq performance in real-world laboratory setting	
RNA-seq performance	RNA-seq still presents challenges in two aspects:<br>• detecting subtle differential expression.<br>• real-world scenarios characterized by a lack of quality control and significantly diverse workflows.
2. The sources of variations among laboratories	
Experimental process	**The magnitude of the variation**<br>• Significantly exceeding the biological differences among Quartet samples but remaining smaller than those between MAQC samples
	**Sources of variation**<br>• mRNA enrichment method, strandedness, library kits, reads length, and exonic reads.
Bioinformatics process	**The magnitude of the variation**<br>• Comparable to variations from the experimental process and exceeding the biological differences between the Quartet samples.
	**Sources of variation**<br>• Primarily normalization, followed by quantification, alignment, annotation.
3. Best practices for experimental and bioinformatics design and quality control of RNA-seq	
Experimental design	**General principles**<br>• The quality of experimental execution is more important than the choices of experimental protocols.<br>• The choice of experimental protocols should be determined by the research goals (sample types and quality, and gene types of interest).
	**Impact of specific experimental factors**<br>• Data quality (SNR): Poly(A) selection method is higher for protein-coding genes, while the rRNA depletion method is higher for non-coding genes.

	• Absolute expression: rRNA depletion method and stranded library are more accurate. Extremely low sequencing depth influences accuracy.<br>• Relative expression and DEG identification: longer read length, higher sequencing depth (especially exonic reads) are associated with higher accuracy.<br>Factors to be validated: Sequencing platform, library kit, RNA input, insert size.
Bioinformatics design	**Normalization**<br>• TMM or DESeq
	**Gene annotation**<br>• For quantification purpose, choose Ensembl annotation when using exon-level quantification tools, RefSeq when using transcript-level quantification tools.<br>• For DEG analysis purpose, Ensembl consistently exhibits higher or comparable accuracy.
	**Alignment**<br>• The combination of Ensembl annotation and STAR exhibits high alignment rates.<br>• Alignment tools have impacts on the RNA-seq performance, but their impacts are less than other bioinformatics steps.<br>• The selection of alignment tools should consider factors such as the genome complexity of samples, and characteristics of sequencing data[16-18].
	**Quantification**<br>• For gene-level analysis, genome-alignment quantification tools, such as featureCounts, HTSeq, and StringTie, are more accurate than transcriptome-alignment or alignment-free quantification tools. Combination of Salmon and RefSeq outperforms other transcriptome-alignment and alignment-free quantification pipelines.<br>**Filtering of low-expression genes**<br>• The filtering thresholds vary with different samples and analysis pipelines.<br>• If benchmark datasets are available, balancing TPR and precision is feasible; otherwise, maximizing the number of DEGs is an efficient approach.
	**Differential analysis tools**<br>• edgeR and DESeq2.

Quality control	**Reference materials**<br>• Reference materials with small biological differences are required to ensure the quality of detecting clinically relevant subtle differential expression.<br>• Reference materials with subtle differential expression allow for more precise assessment of RNA-seq quality, and are more sensitive in uncovering issues within the RNA-seq system.
	**Basic quality metrics**<br>• Sequencing QC: sequencing depth, base quality scores, duplication rate, GC content, insert size[19].<br>• Alignment QC: gene coverage bias, gene mapping rate, reads mapped to the exonic regions[19].<br>• Sample or library QC: sample swaps or mislabeling, cross-contamination
	**RNA-seq performance assessment framework**<br>• Expression data quality: PCA-based SNR.<br>• Accuracy of gene expression: RMSE or CC.<br>• Accuracy of DEG identification: penalized MCC.

SNR, Signal-to-Noise Ratio; RMSE, Root Mean Square Error; CC, Correlation Coefficient; MCC, Matthews Correlation Coefficient; QC, Quality Control.

3. Supplementary Notes

3.1 The comprehensive performance assessment framework for RNA-seq data

The comprehensive performance assessment framework involved multifaceted properties of transcriptome data, including data quality, gene expression, and differential gene expression (**Fig. 1b**).

First, the quality of expression data was quantified using the PCA-based signal-to-noise ratio (SNR) values, which measures the laboratory's ability to distinguish biological signals between different samples from the technical noise of replicates. PCA-based SNR values were highly effective in discriminating data quality across laboratories, especially when working with Quartet samples characterized by subtle biological differences.

Second, the gene expression was examined by calculating the accuracy and reproducibility of absolute and relative expression. Our assessment extended to cover 92 ERCC genes, hundreds of genes in the TaqMan datasets, tens of thousands of genes in Quartet reference datasets, and all genes through examining the mixing ratios recovery. The inclusion of a broader set of gene significantly enhanced the assessment precision.

Third, the number of differentially expressed genes (DEGs) were compared across laboratories, and the accuracy of DEG calls were evaluated based on the Quartet reference datasets and the TaqMan datasets for Quartet and MAQC samples.

This comprehensive performance assessment framework addressed several challenges (**Supplementary Figure 59**). (i) the study addressed challenges related to gene annotations from different sources and versions used in real-world laboratories, which typically hindered comparisons across laboratories and reference datasets[20]. We matched gene IDs of different types and versions to generate an intersection gene set, facilitating comparisons of transcriptome data. (ii) the data quality, differential analysis tools, and filtering parameters all influence the differential gene expression list, which may lead to certain genes present in reference datasets being missed, raising the question of how to categorize these genes in performance metric calculation. We excluded genes unreported by laboratories due to the choice of different gene annotations and defined the remaining unreported genes labeled as DEGs in the reference dataset as false negatives for calculating the MCC metric. Altogether, this performance assessment framework facilitates the integration and comparison of real-world transcriptome data. Moving forward, with the emergence of omics reference

datasets like Quartet, there is a growing need for standardized benchmarking tools available for open access, providing a one-stop solution for comprehensive RNA-seq performance assessment[21].

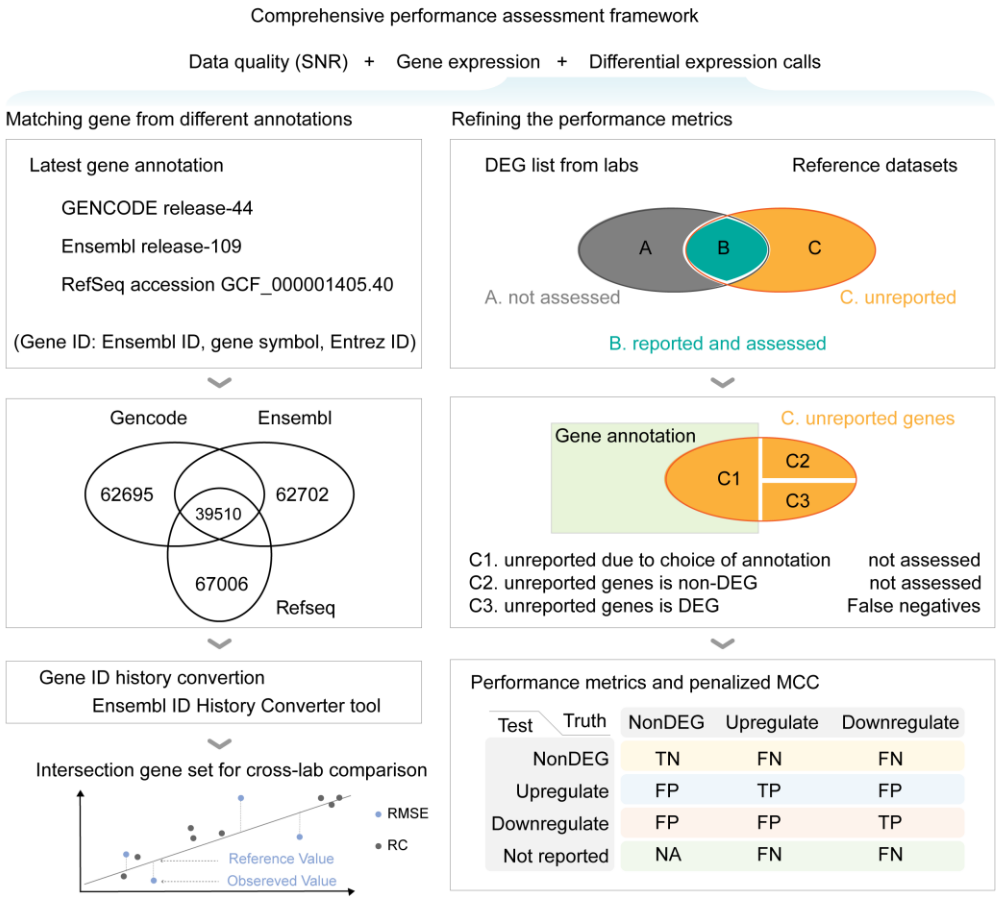

Supplementary Figure 59. The performance assessment framework for RNA-seq data.

3.2 RNA-seq assessment based on ERCC spike-in controls.

The reads mapped to the ERCC genes should account for approximately 1% of the total exonic reads, providing an assessment of library quality. Laboratories demonstrated significant variations regarding the percentage of reads mapped to ERCC **(Supplementary Fig. 60)**. The rRNA depletion protocol correlated to a higher fraction of reads aligned to ERCC genes, while the Poly(A) selection protocol exhibited a lower mapping ratio but better recovered the relative ratio among the four subgroups of ERCC sequences **(Supplementary Fig. 61)**.

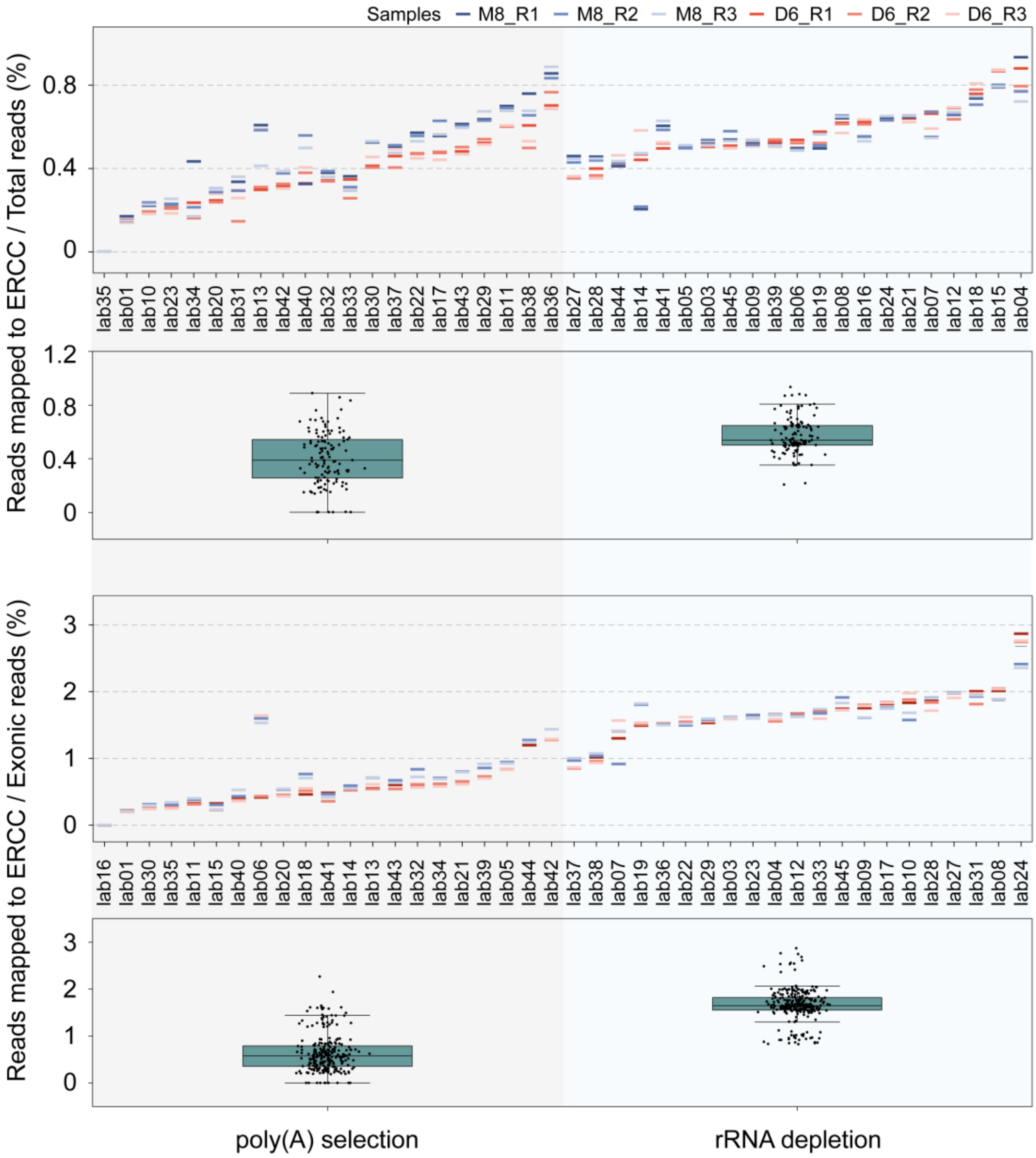

Supplementary Figure 60. Percentage of reads mapped to ERCC genes. The box plots present the ratios between reads mapped to ERCC genes and total reads or exonic reads in the Quartet M8 and D6 samples (n = 126) for laboratories using different

mRNA enrichment methods, and data are presented as median values (center lines) and the upper and lower quartiles (box limits). The rRNA depletion protocol correlated to a higher fraction of reads mapped to ERCC genes.

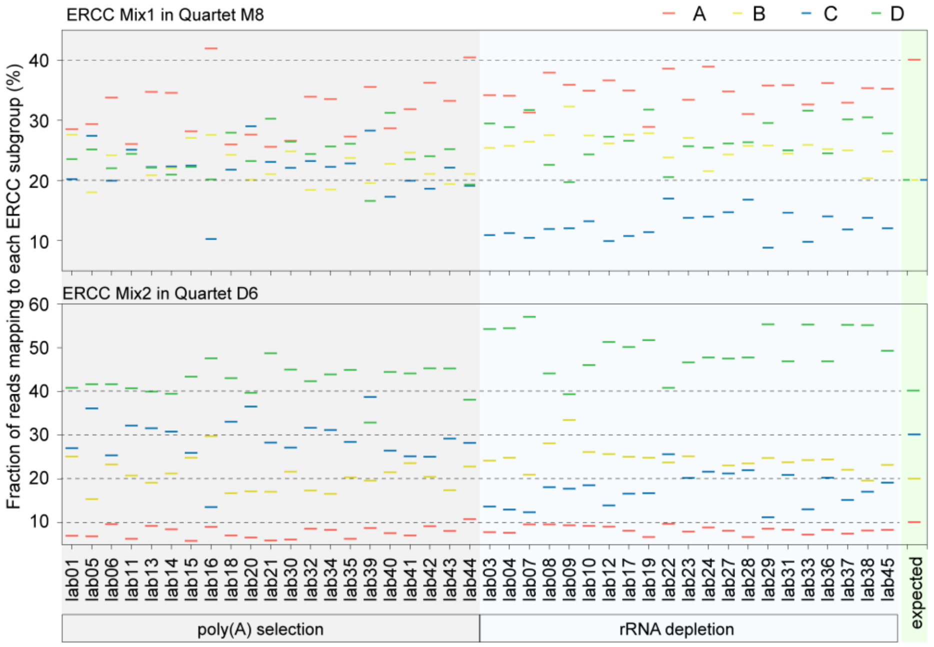

Supplementary Figure 61. Percentage of reads mapped to four subgroups of ERCC genes. Both ERCC Mix 1 and Mix 2 contain genes from four subgroups labeled as A, B, C, and D, each with 23 genes. The expected ratios of A, B, C, and D, are 2:1:1:1 and 1:2:3:4 in Mix 1 and Mix 2, respectively[22]. The Poly(A) selection protocol associated with higher accuracy of recovering the intrinsic ratios.

3.3 The number of detected genes in Quartet and MAQC samples.

Based on gene count data from 45 laboratories, each employing their own analysis pipeline, we compared the number of detected or expressed genes across all laboratories after applying different thresholds. Three types of thresholds previously used by Yu et al.[23] and the MAQC-III Consortium[24] were incorporated, including with at least one read in any one technical replicate, with at least 10 reads in any one technical replicate, and with at least 3 reads in at least two out of three replicates. When using different threshold, the MAQC samples consistently exhibited a higher number of detected genes across laboratories, compared to the Quartet samples and the mixed samples. The substantial variations in the number of detected genes among laboratories were mainly associated with specific gene types of interest and sequencing depth **(Supplementary Fig. 62)**. Noticeably, the relatively lower number of detected genes in some laboratories despite with high depth could be attributed to low read alignment rates, high duplication rates. Furthermore, the choice of alignment tools and gene annotations can also impact the number of detected genes as mentioned in previous studies[17, 25]. Similarly, increased sequencing depth contributed to the detection of more ERCC genes **(Supplementary Fig. 63)**.

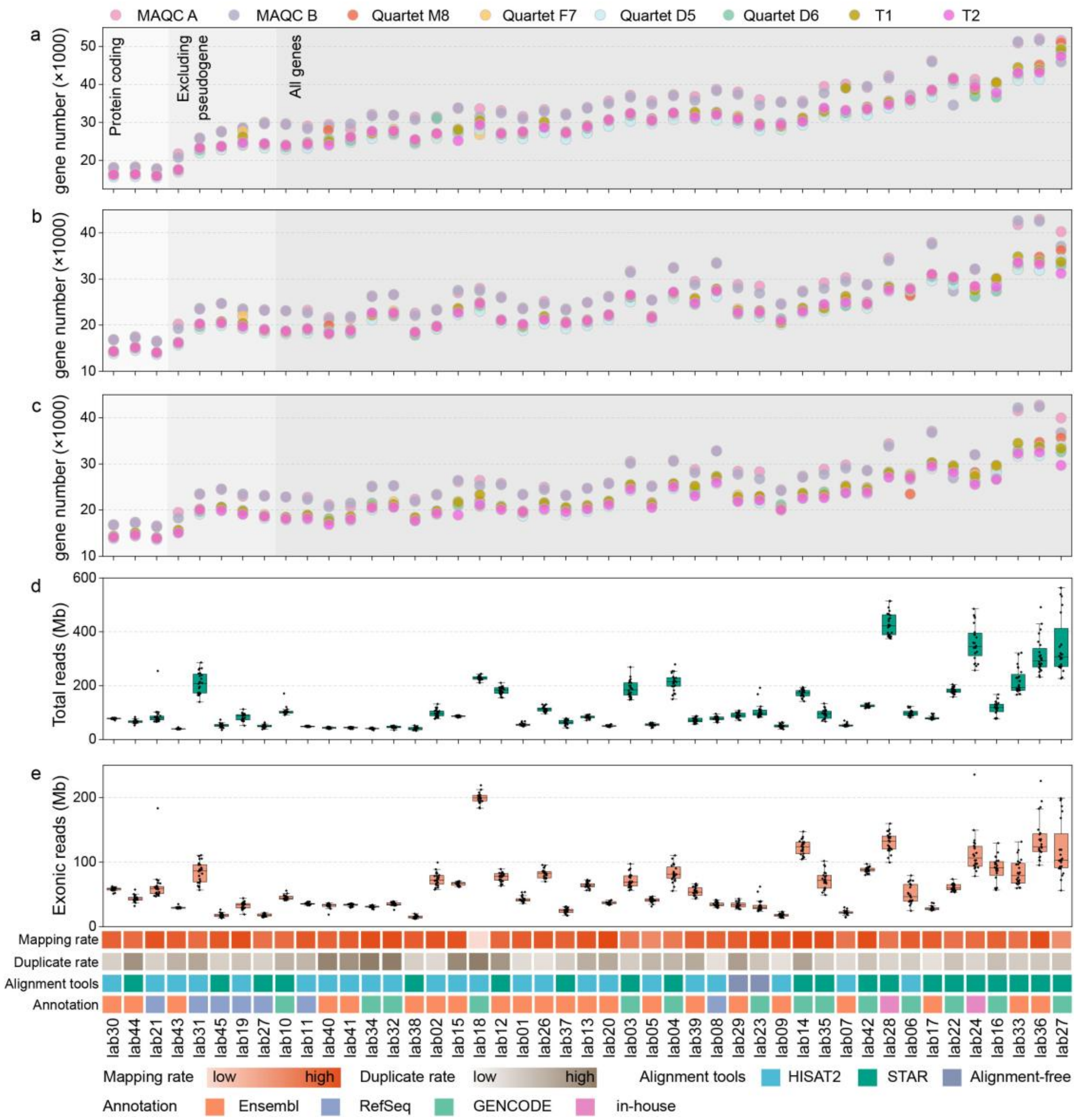

Supplementary Figure 62. The number of genes detected by all laboratories. Genes are considered detectable (expressed) in a sample group if there is **(a)** at least one read in any one technical replicate, **(b)** at least 10 reads in any one technical replicate, and **(c)** more than three reads in at least two out of the three replicates. Three laboratories exclusively focused on protein-coding genes for downstream analysis, five laboratories excluded pseudogenes from their analysis, while the remaining 37 laboratories analyzed genes of all biotypes. **(d)** The number of sequencing reads across laboratories. **(e)** The number of sequencing reads mapped to the exonic regions across laboratories. Higher sequencing depth generally leads to the detection of more genes, although exceptions exist, such as when alignment rates are low or duplication rates are high.

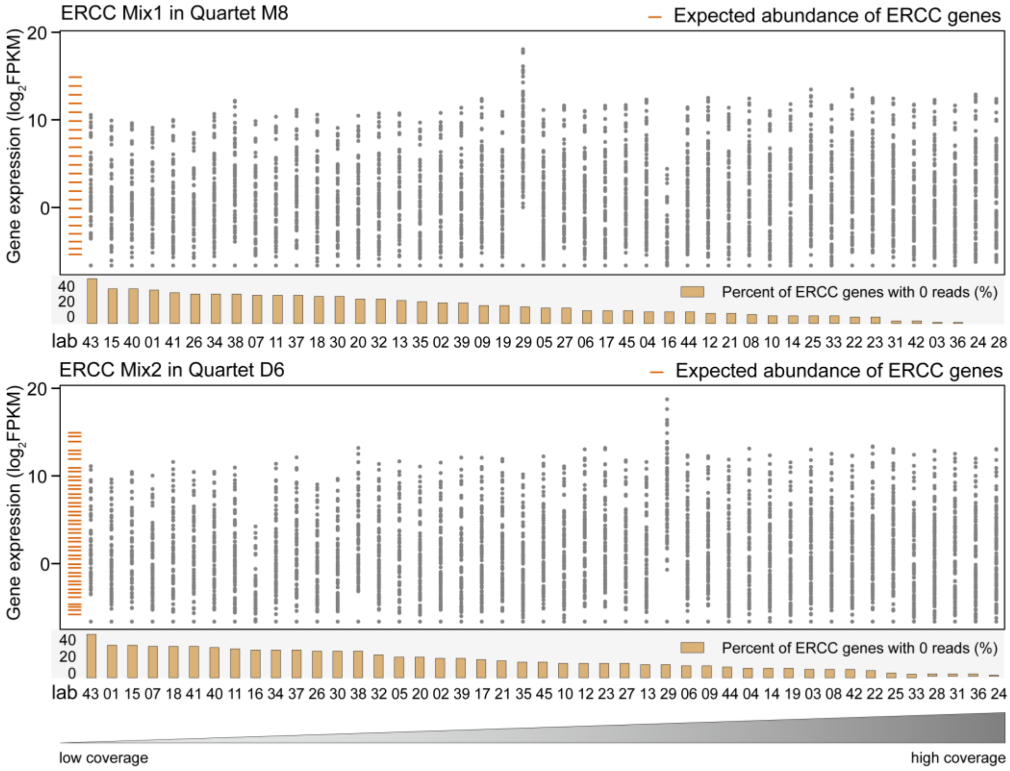

Supplementary Figure 63. The number of ERCC genes detected by 42 laboratories. With the increase of sequencing depth, more ERCC genes were supported by at least one reads.

Reference

1. Chen, S., Zhou, Y., Chen, Y. & Gu, J. fastp: an ultra-fast all-in-one FASTQ preprocessor. *Bioinformatics* **34**, i884-i890 (2018).
2. Parekh, S., Ziegenhain, C., Vieth, B., Enard, W. & Hellmann, I. The impact of amplification on differential expression analyses by RNA-seq. *Sci Rep* **6**, 25533 (2016).
3. Li, S. et al. Multi-platform assessment of transcriptome profiling using RNA-seq in the ABRF next-generation sequencing study. *Nat Biotechnol* **32**, 915-925 (2014).
4. Bansal, V. A computational method for estimating the PCR duplication rate in DNA and RNA-seq experiments. *BMC Bioinformatics* **18**, 43 (2017).
5. Picard toolkit. *Broad Institute, GitHub repository* (2019).
6. Sayols, S., Scherzinger, D. & Klein, H. dupRadar: a Bioconductor package for the assessment of PCR artifacts in RNA-Seq data. *BMC Bioinformatics* **17**, 428 (2016).
7. Dobin, A. et al. STAR: ultrafast universal RNA-seq aligner. *Bioinformatics* **29**, 15-21 (2013).
8. Lee, S. et al. NGSCheckMate: software for validating sample identity in next-generation sequencing studies within and across data types. *Nucleic Acids Res* **45**, e103 (2017).
9. Seqtk, https://github.com/lh3/seqtk.git (2023).
10. Martin, M. Cutadapt removes adapter sequences from high-throughput sequencing reads. *EMBnet.journal; Vol 17, No 1: Next Generation Sequencing Data AnalysisDO - 10.14806/ej.17.1.200* (2011).
11. Wang, L., Wang, S. & Li, W. RSeQC: quality control of RNA-seq experiments. *Bioinformatics* **28**, 2184-2185 (2012).
12. Ritchie, M.E. et al. limma powers differential expression analyses for RNA-sequencing and m icroarray studies. *Nucleic Acids Res* **43**, e47.
13. Love, M.I., Huber, W. & Anders, S. Moderated estimation of fold change and dispersion for RNA-seq data wi th DESeq2. *Genome biology* **15**, 550.
14. Wang, L., Feng, Z., Wang, X., Wang, X. & Zhang, X. DEGseq: an R package for identifying differentially expressed genes fr om RNA-seq data. *Bioinformatics (Oxford, England)* **26**, 136-138.
15. Leng, N. et al. EBSeq-HMM: a Bayesian approach for identifying gene-expression changes in ordered RNA-seq experiments. *Bioinformatics (Oxford, England)* **31**, 2614-2622.
16. Yang, C., Wu, P.Y., Tong, L., Phan, J.H. & Wang, M.D. The impact of RNA-seq aligners on gene expression estimation. *Acm bcb* **2015**, 462-471 (2015).
17. Szabelska-Beresewicz, A., Zyprych-Walczak, J., Siatkowski, I. & Okoniewski, M. Ambiguous genes due to aligners and their impact on RNA-seq data analysis. *Sci Rep* **13**, 21770 (2023).
18. Baruzzo, G. et al. Simulation-based comprehensive benchmarking of RNA-seq aligners. *Nat Methods* **14**, 135-139 (2017).
19. t Hoen, P.A. et al. Reproducibility of high-throughput mRNA and small RNA sequencing across laboratories. *Nat Biotechnol* **31**, 1015-1022 (2013).
20. Breidenbach, J.D., Begue Iii, E.F., Kennedy, D.J. & Haller, S.T. GeneToList: A Web Application to Assist with Gene Identifiers for the Non-Bioinformatics-Savvy Scientist. *Biology (Basel)* **11** (2022).
21. Krusche, P. et al. Best practices for benchmarking germline small-variant calls in human genomes. *Nat Biotechnol* **37**, 555-560 (2019).
22. Munro, S.A. et al. Assessing technical performance in differential gene expression experiments with external spike-in RNA control ratio mixtures. *Nature Communications* **5**, 5125 (2014).

23. Yu, Y. et al. Quartet RNA reference materials improve the quality of transcriptomic data through ratio-based profiling. *Nat Biotechnol*, 10.1038/s41587-41023-01867-41589 (2023).
24. A comprehensive assessment of RNA-seq accuracy, reproducibility and information content by the Sequencing Quality Control Consortium. *Nat Biotechnol* **32**, 903-914 (2014).
25. Chisanga, D., Liao, Y. & Shi, W. Impact of gene annotation choice on the quantification of RNA-seq data. *BMC Bioinformatics* **23**, 107 (2022).
