# Supplementary material for: A real-world multi-center RNA-seq benchmarking study using the Quartet and MAQC reference materials
Source: Nat Commun. 2024 Jul 22;15:6167. doi: 10.1038/s41467-024-50420-y (PMC11263697; doi:10.1038/s41467-024-50420-y)
Supplement: Supplementary file 3 — Description of Additional Supplementary Files [file 41467_2024_50420_MOESM3_ESM.pdf]

Description of Additional Supplementary Files

Supplementary Data 1: Detailed RNA-seq workflows for all laboratories.

Supplementary Data 2: The potential reason of low MCC for certain laboratories.

Supplementary Data 3: Basic quality control for sequencing data.

Supplementary Data 4: Variables in experimental process used in principal variance component analysis.

Supplementary Data 5: Performance ranking of differential gene expressions analysis tools.

Supplementary Data 6: Sequences of primers and TaqMan probes for RT-qPCR assays.

Supplementary Data 7: TaqMan datasets for the Quartet samples.

Supplementary Data 8: RNA-seq tools, versions, and command line used in the benchmark study.
