# Supplementary material for: A real-world multi-center RNA-seq benchmarking study using the Quartet and MAQC reference materials
Source: Nat Commun. 2024 Jul 22;15:6167. doi: 10.1038/s41467-024-50420-y (PMC11263697; doi:10.1038/s41467-024-50420-y)
Supplement: Supplementary file 12 — Reporting Summary [file 41467_2024_50420_MOESM12_ESM.pdf]

nature portfolio

Corresponding author(s): Leming Shi, Yuanting Zheng, Jinming Li, and Rui Zhang

Last updated by author(s): May 25, 2024

Reporting Summary

Nature Portfolio wishes to improve the reproducibility of the work that we publish. This form provides structure for consistency and transparency in reporting. For further information on Nature Portfolio policies, see our Editorial Policies and the Editorial Policy Checklist.

Statistics

For all statistical analyses, confirm that the following items are present in the figure legend, table legend, main text, or Methods section.

n/a	Confirmed	
☐	☒	The exact sample size (*n*) for each experimental group/condition, given as a discrete number and unit of measurement
☐	☒	A statement on whether measurements were taken from distinct samples or whether the same sample was measured repeatedly
☐	☒	The statistical test(s) used AND whether they are one- or two-sided<br>*Only common tests should be described solely by name; describe more complex techniques in the Methods section.*
☒	☐	A description of all covariates tested
☐	☒	A description of any assumptions or corrections, such as tests of normality and adjustment for multiple comparisons
☐	☒	A full description of the statistical parameters including central tendency (e.g. means) or other basic estimates (e.g. regression coefficient) AND variation (e.g. standard deviation) or associated estimates of uncertainty (e.g. confidence intervals)
☐	☒	For null hypothesis testing, the test statistic (e.g. *F*, *t*, *r*) with confidence intervals, effect sizes, degrees of freedom and *P* value noted<br>*Give P values as exact values whenever suitable.*
☒	☐	For Bayesian analysis, information on the choice of priors and Markov chain Monte Carlo settings
☒	☐	For hierarchical and complex designs, identification of the appropriate level for tests and full reporting of outcomes
☐	☒	Estimates of effect sizes (e.g. Cohen's *d*, Pearson's *r*), indicating how they were calculated

Our web collection on statistics for biologists contains articles on many of the points above.

Software and code

Policy information about availability of computer code

Data collection	Software: STAR (v.2.7.10b), HISAT2 (v.2.2.1), Subread (v.2.0.3), featureCounts (v.2.0.3), HTSeq (v.2.0.2), StringTie (v.2.2.1), RSEM (v.1.3.1), Bowtie (v.1.3.1), kallisto (v.0.48.0), Salmon (v.1.10.1), Sailfish (v.0.9.0), edgeR (v.3.42.4), limma (v.3.56.2), DESeq2 (v.1.40.2), DEGseq (v.1.54.0), EBSeq (v.1.40.0), RSeQC (v.5.0.1), Picard (3.1.1), Cutadapt (v.4.8), dupRadar (v.3.18), seqtk (v.1.4),FastQC (v.0.12.1), fastp (v.0.23.2), Qualimap (v.2.2.2), MultiQC (v.1.8), NGSCheckMate (v.1.0.1), pvca(v.1.40.0), NOISeq (v.2.44.0). The integrated data analysis pipelines are available on Github at https://github.com/lyaqing/snakemake_rnaseq.
Data analysis	Software: R v.4.3.0 and python v.3.10.10. All custom scripts for data analysis are available on Github at https://github.com/wangduo-ux/Asssessment-of-RNA-seq-performance.git.

For manuscripts utilizing custom algorithms or software that are central to the research but not yet described in published literature, software must be made available to editors and reviewers. We strongly encourage code deposition in a community repository (e.g. GitHub). See the Nature Portfolio guidelines for submitting code & software for further information.

Data

Policy information about availability of data

All manuscripts must include a data availability statement. This statement should provide the following information, where applicable:

- Accession codes, unique identifiers, or web links for publicly available datasets
- A description of any restrictions on data availability
- For clinical datasets or third party data, please ensure that the statement adheres to our policy

The high-quality raw sequence data reported in this paper have been deposited in the Genome Sequence Archive (Genomics, Proteomics & Bioinformatics 2021) in National Genomics Data Center (Nucleic Acids Res 2022), China National Center for Bioinformation / Beijing Institute of Genomics, Chinese Academy of Sciences (GSA-Human: HRA005937) that are publicly accessible at https://ngdc.cncb.ac.cn/gsa-human.

Research involving human participants, their data, or biological material

Policy information about studies with human participants or human data. See also policy information about sex, gender (identity/presentation), and sexual orientation and race, ethnicity and racism.

Reporting on sex and gender	We used RNA reference materials that do not involve gender. The sources of these reference materials have been reported in DOI: 10.1038/nbt.2957 and DOI: 10.1038/s41587-023-01867-9.
Reporting on race, ethnicity, or other socially relevant groupings	We used RNA reference materials that do not involve race and ethnicity.
Population characteristics	We used Quartet RNA reference materials derived from immortalized B-lymphoblastoid cell lines from a Chinese quartet family of parents and monozygotic twin daughters, which has been reported in DOI: 10.1038/s41587-023-01867-9.
Recruitment	We used RNA reference materials directly and did not recruit participants.
Ethics oversight	Our study does not involve ethical considerations

Note that full information on the approval of the study protocol must also be provided in the manuscript.

Field-specific reporting

Please select the one below that is the best fit for your research. If you are not sure, read the appropriate sections before making your selection.

☒ Life sciences ☐ Behavioural & social sciences ☐ Ecological, evolutionary & environmental sciences

For a reference copy of the document with all sections, see nature.com/documents/nr-reporting-summary-flat.pdf

Life sciences study design

All studies must disclose on these points even when the disclosure is negative.

Sample size	As a RNA-seq performance assessment study, we used 24 RNA samples (8 groups including MAQC A, B, Quartet M8, F7, d5, D6, T1, and T2) and generated RNA-seq data across 45 laboratories. In total, 1,080 RNA-seq libraries were prepared, yielding a dataset of over 120 billion reads (15.63 Tb) for the Quartet and MAQC samples.
Data exclusions	All data from 45 laboratories have been included.
Replication	The reference materials were tested in a lab in three replicates for each of 8 types of sample.
Randomization	Aliquots of RNA from the same lot were randomly distributed to each laboratory.
Blinding	To reflect real-world scenarios, samples were blinded for objective assessment of lab proficiency.

Reporting for specific materials, systems and methods

We require information from authors about some types of materials, experimental systems and methods used in many studies. Here, indicate whether each material, system or method listed is relevant to your study. If you are not sure if a list item applies to your research, read the appropriate section before selecting a response.

Materials & experimental systems

n/a	Involved in the study
☒	☐ Antibodies
☒	☐ Eukaryotic cell lines
☒	☐ Palaeontology and archaeology
☒	☐ Animals and other organisms
☒	☐ Clinical data
☒	☐ Dual use research of concern
☒	☐ Plants

Methods

n/a	Involved in the study
☒	☐ ChIP-seq
☒	☐ Flow cytometry
☒	☐ MRI-based neuroimaging

Plants

Seed stocks	*Report on the source of all seed stocks or other plant material used. If applicable, state the seed stock centre and catalogue number. If plant specimens were collected from the field, describe the collection location, date and sampling procedures.*
Novel plant genotypes	*Describe the methods by which all novel plant genotypes were produced. This includes those generated by transgenic approaches, gene editing, chemical/radiation-based mutagenesis and hybridization. For transgenic lines, describe the transformation method, the number of independent lines analyzed and the generation upon which experiments were performed. For gene-edited lines, describe the editor used, the endogenous sequence targeted for editing, the targeting guide RNA sequence (if applicable) and how the editor was applied.*
Authentication	*Describe any authentication procedures for each seed stock used or novel genotype generated. Describe any experiments used to assess the effect of a mutation and, where applicable, how potential secondary effects (e.g. second site T-DNA insertions, mosiacism, off-target gene editing) were examined.*
